# Detection and evaluation of parameters influencing the identification of heterozygous-enriched regions in Holstein cattle based on SNP chip or whole-genome sequence data

**DOI:** 10.1186/s12864-024-10642-2

**Published:** 2024-07-26

**Authors:** Henrique A. Mulim, Victor B. Pedrosa, Luis Fernando Batista Pinto, Francesco Tiezzi, Christian Maltecca, Flavio S. Schenkel, Luiz F. Brito

**Affiliations:** 1 Department of Animal Sciences, Federal University of Bahia, Salvador, Bahia, 40110-909 Brazil; 2 Department of Animal Sciences, Purdue University, West Lafayette, Indiana, 47907 USA; 3 Department of Animal Sciences, State University of Ponta Grossa, Ponta Grossa, Parana, 84010-330 Brazil; 4 Department of Agriculture, Food, Environment and Forestry, University of Florence, 50121 Florence, Italy; 5 Department of Animal Science, North Carolina State University, Raleigh, NC 27607 USA; 6 Centre for Genetic Improvement of Livestock (CGIL), Department of Animal Biosciences, University of Guelph, Ontario, N1G 2W1 Canada

**Keywords:** Balancing selection, Genetic diversity, Heterozygous advantage, Livestock genetics

## Abstract

**Background:**

A heterozygous-enriched region (HER) is a genomic region with high variability generated by factors such as balancing selection, introgression, and admixture processes. In this study, we evaluated the genomic background of HERs and the impact of different parameters (i.e., minimum number of SNPs in a HER, maximum distance between two consecutive SNPs, minimum length of a HER, maximum number of homozygous allowed in a HER) and scenarios [i.e., different SNP panel densities and whole-genome sequence (WGS)] on the detection of HERs. We also compared HERs characterized in Holstein cattle with those identified in Angus, Jersey, and Norwegian Red cattle using WGS data.

**Results:**

The parameters used for the identification of HERs significantly impact their detection. The maximum distance between two consecutive SNPs did not impact HERs detection as the same average of HERs (269.31 ± 787.00) was observed across scenarios. However, the minimum number of markers, maximum homozygous markers allowed inside a HER, and the minimum length size impacted HERs detection. For the minimum length size, the 10 Kb scenario showed the highest average number of HERs (1,364.69 ± 1,483.64). The number of HERs decreased as the minimum number of markers increased (621.31 ± 1,271.83 to 6.08 ± 21.94), and an opposite pattern was observed for the maximum homozygous markers allowed inside a HER (54.47 ± 195.51 to 494.89 ± 1,169.35). Forty-five HER islands located in 23 chromosomes with high Tajima’s D values and differential among the observed and estimated heterozygosity were detected in all evaluated scenarios, indicating their ability to potentially detect regions under balancing selection. In total, 3,440 markers and 28 genes previously related to fertility (e.g., *TP63, ZSCAN23, NEK5, ARHGAP44*), immunity (e.g., *TP63, IGC, ARHGAP44*), residual feed intake (e.g., *MAYO9A*), stress sensitivity (e.g., *SERPINA6*), and milk fat percentage (e.g., *NOL4*) were identified. When comparing HER islands among breeds, there were substantial overlaps between Holstein with Angus (95.3%), Jersey (94.3%), and Norwegian Red cattle (97.1%), indicating conserved HER across taurine breeds.

**Conclusions:**

The detection of HERs varied according to the parameters used, but some HERs were consistently identified across all scenarios. Heterozygous genotypes observed across generations and breeds appear to be conserved in HERs. The results presented could serve as a guide for defining HERs detection parameters and further investigating their biological roles in future studies.

**Supplementary Information:**

The online version contains supplementary material available at 10.1186/s12864-024-10642-2.

## Background

Assessing and developing strategies for maintaining genomic diversity in dairy cattle populations have become important activities in breeding programs due to the faster accumulation of inbreeding per year and reduction in effective population sizes as a result of intensive breeding practices (e.g., artificial insemination) and genomic selection schemes [1–3]. Numerous studies have characterized homozygous regions (e.g., runs of homozygosity – ROH) in several livestock species, including cattle [4–6], swine [7, 8], sheep [9], goats [10], horses [11], and poultry [12]. For instance, ancient and recent genomic inbreeding can be estimated based on ROH [13]. On the other hand, a stretch of heterozygous genotypes, also known as heterozygous-enriched region (HER) or runs of heterozygosity (ROHet), are far less characterized than ROH in livestock species [14] and could indicate genomic regions with high genetic variability and balancing selection.

The first study reporting HERs in livestock was published by Williams et al. [15] in Chillingham cattle. This breed had not been subjected to artificial selection and remained a closed herd for at least 350 years [15]. Despite this particularity, some genomic regions were still found to have high concentrations of heterozygous genotypes, especially regions containing loci influencing fitness and survival traits. Following this first HER characterization, studies on sheep [9], cattle [16], horses [11], and pigs [17] have been published. As such, substantial evidence indicates that genomic regions linked to some essential traits maintain high heterozygosity across generations. Maintaining haplotypic diversity at specific loci might confer a fitness advantage and be subject to balancing selection [15].

Balancing selection describes any selection processes that result in the maintenance of multiple variants of specific loci at intermediate frequencies within a population [18]. Balancing selection implies that heterozygosity is widespread and persists in the population through heterozygote advantage [19]. Balancing selection is not the only process linked to HERs occurrence in a population. Other processes such as introgression (the transfer of genetic variants from one species to another, e.g., hybridization), admixture (mixture of genetic lines or breeds – usually in the same species), and hypervariable regions (portions of the genome with much higher levels of variation than other similar areas due to mutations, recombination rate, and chromosomal rearrangements) [12, 15] contribute to the appearance and maintenance of HERs. A high concentration of heterozygous alleles in specific genomic regions across a large proportion of a population is defined as HER islands and could inform balancing selection pressure suffered by a population at a specific time.

The accurate assessment of polymorphisms in high-variable genomic regions presents additional challenges and can lead to underestimation of the results [19]. One of the main challenges when quantifying HERs in a population is the definition of the parameters to be used in the analyses. For instance, HER detection can be impacted by the density of the SNP panels, distribution of markers throughout the genome, genotyping quality, and consistency of information (error rates and minor allele frequencies) [20]. Aside from these parameters, the correct identification of HERs also depends on factors such as the minimum length size of a HER and the number of homozygous allowed within a HER [21]. Despite the influence of all these factors in the identification of HERs and HER islands, to the best of our knowledge, no studies have evaluated the impact of different parameters used in HER detection. Therefore, the main objectives of this study were to: 1) evaluate the impact of different parameters (i.e., minimum number of SNPs in a HER, maximum distance between two consecutive SNPs, minimum length of a HER, maximum number of homozygous allowed in a HER) and data source scenarios (i.e., SNP panel densities and whole-genome sequence data – WGS) on the detection of HERs; 2) characterize HERs in Holstein (HOL) cattle based on WGS data, followed by functional genomic analyses of the identified HER islands; and, 3) evaluate the overlap of the HERs found in HOL with those from other taurine (Bos taurus taurus) breeds, including Angus (ANG), Jersey (JER), and Norwegian Red cattle (RDC).

## Methods

### Data and quality control

Nine hundred and fifty-nine (959) HOL animals from the 1,000 Bull Genomes Project [22] were used in this study. WGS data for these individuals contained 47,379,463 markers distributed across the 29 autosomes. The quality control (QC) was performed following the criteria proposed by Ferenčaković et al. [23] and Biscarini et al. [14] in HER and ROH studies. In brief, the QC removed SNPs with low call rate (< 0.95), duplicated positions, located on non-autosomal chromosomes, or with unknown positions.

### Identification of Heterozygous-enriched Regions

The detectRUNs package [24] was used to identify HERs, applying the “consecutive approach” in the analyses, which directly scans the genome SNP by SNP, as proposed by Marras et al. [25]. The impact of different sets of parameters in the identification of HERs was evaluated. These parameters included:

- Minimum number of SNPs in a HER: 5, 10, 15, 20, 25, and 30;
- Maximum distance between two consecutive SNPs (GAP, in Kb): 500 Kb, 1,000 Kb, and 2,000 Kb;
- Minimum length of a HER (Kb): 10 Kb, 25 Kb, 50 Kb, 100 Kb, 500 Kb, and 1,000 Kb; and,
- Maximum number of homozygous allowed in a HER: 0, 1, 2, 3, 4, and 5.

The combination of all sets of parameters resulted in 648 analyses. Therefore, the following criteria were employed to optimize time and computing efficiency. A subset of 300 HOL animals was randomly chosen from the 959 available. Furthermore, the impact of the parameters on HERs identification was evaluated on three selected chromosomes with 5,351,067 markers (BTA1: 2,987,435 markers, BTA14: 1,544,553 markers, and BTA25: 819,079 markers). These chromosomes were selected to represent long, medium, and short chromosomes in the cattle genome, respectively, providing an overall view of how the parameters chosen for the analyses impact HER detection across the entire genome. As the number of HERs detected is based on a combination of parameters, the average and standard deviation of the number of HERs detected, corresponding to each parameter, were calculated to assess the impact of each parameter on HERs detection. For instance, the effect of the minimum number of SNPs equal to 5 on HERs detection was measured by the average and standard deviation of the total number of HERs detected across all scenarios with a minimum number of SNPs equal to 5. Further, we evaluated the effect of all the parameters on HERs when using data from SNP chip panels instead of WGS. Three SNP panels were derived from WGS data by selectively retaining variants present in commercial SNP panels: 50 K (BovineSNP50), 100 K (GGP Bovine 100 K), and HD (700 K – BovineHD genotype BeadChip).

Lastly, additional scenarios were created as preliminary results about the impact of parameters did not show a relevant differentiation based on the minimum number of SNPs and homozygous allowed (see Results section). Therefore, we considered the minimum number of markers to determine a HER equal to 5 and 10, combining the homozygous allowed inside of a HER, ranging from 0 to 5 (0–2 when the minimum number of markers was equal to 5, and 0–5 for minimum number of markers equal 10), and fixing the minimum length size of a HER in 10 Kb, resulting in nine additional scenarios. This approach was used to identify the best combination of parameters to detect HERs, using all the autosomal chromosomes.

### Heterozygosity estimation, nucleotide diversity, and Tajima’s D statistic test

Genetic variation in a population can be measured in several ways, the most common of which are heterozygosity (observed and expected) and the proportion of polymorphic nucleotide sites (π) [26]. Using such information, it is possible to estimate if the markers have been selected for heterozygous or homozygous alleles by estimating Tajima’s D value. Tajima’s D test is a good indicator of balancing selection, because it directly measures allele frequency and, since population size change and population structure should affect all loci, differences in Tajima’s D value between loci are likely to reflect differences in selection pressure [27]. It is possible to access the Tajima’s D value by the equation:

$$D=\frac{\frac{\pi-S}{a_{1}}}{\sqrt{V}},$$

Where, π is proportion of polymorphic nucleotide sites, *S* is the number of segregation sites, *V* is the sampling variance of the difference between π and *S,* and ${a}_{1}=\sum_{i=1}^{n-1}\frac{1}{i}$ is the coefficient related to dependent to the number of sequences (n). Here we compared the results of such metrics with the proportion of times that a marker appears on a HER, aiming to validate the parameters chosen.

Each marker’s observed and expected heterozygosity levels were obtained using the Hardy–Weinberg test statistics implemented in the PLINK v.1.09 software [28]. VCFtools [29] was used to estimate each marker’s nucleotide diversity and to perform the Tajima’s D test [30]. The difference between observed and expected heterozygosity, nucleotide diversity, and Tajima’s D statistic were collectively used to correlate with the results from the proportion of times the SNP appeared on a HER in the population, using the Spearman rank correlation to assess the ranking inside of the diversity metrics and the proportion of times the SNP appeared on a HER in the population in the different scenarios.

### Identification of HER islands, linkage disequilibrium, and genomic annotation

**Fig. 1:**
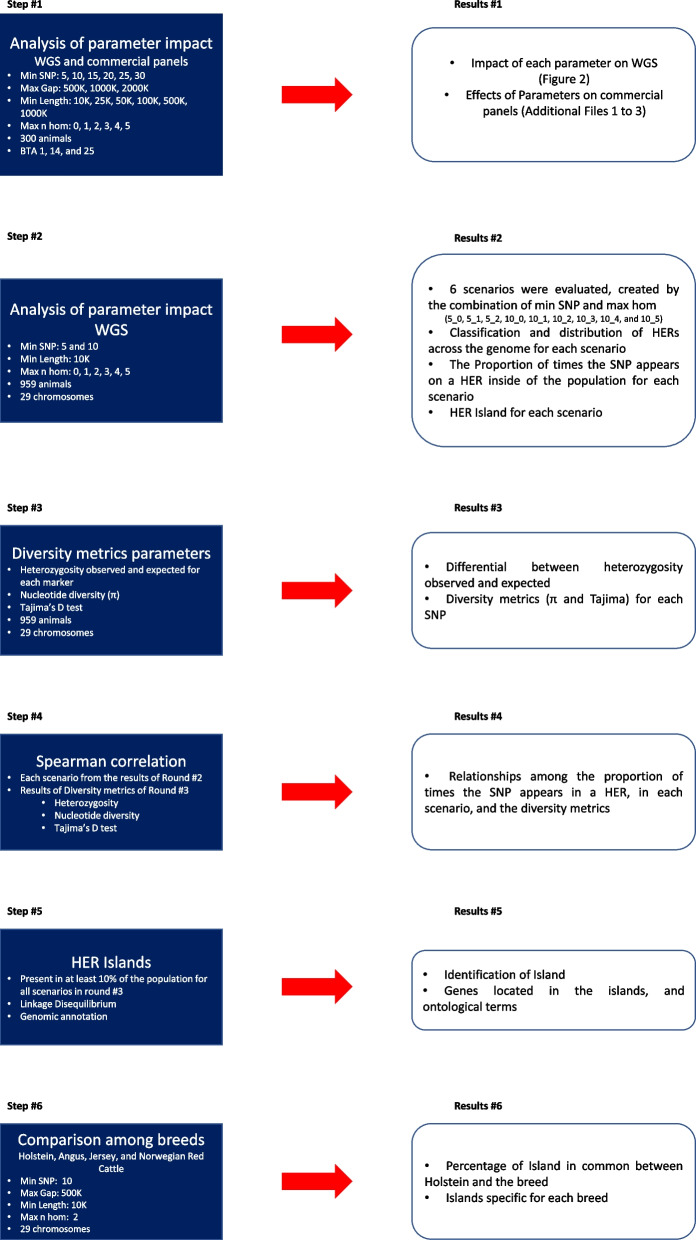
Roadmap of the analyses carried out in this study. Min SNP: minimum number of SNPs in a HER; Max Gap: maximum distance between two consecutive SNPs (Kb); Min Length: minimum length of a HER (Kb); Max n hom: Maximum number of homozygous allowed in a HER

We declared as HER island regions those that were present in at least 10% of the individuals and present in each one of the additional scenarios created on the step #2 (Fig. 1). As specific criteria for defining HER islands were lacking in the literature, we opted for a 10% threshold to serve as a representative value, offering a visual depiction of the overall concentration of HERs across the genome. The linkage disequilibrium (LD) for all markers within these HER islands was estimated using the LDheatmap package [31]. The genomic annotation of these regions was performed using the GALLO R package [32] with the annotated data for *Bos taurus* from the Ensembl database (www.ensembl.org/Bos_taurus/Info/Index), version ARS-UCD1.2 [33]. Subsequently, the WebGestaltR package [34] was used to mine Gene Ontology (GO) and identify potential biological processes, molecular functions, cellular components, and metabolic pathways in which the positional candidate genes might be involved.

### Comparison of HER islands from Holstein with other taurine breeds

The HER islands identified in HOL were compared to those from other three taurine (*Bos taurus taurus*) breeds [ANG (317 animals), JER (179 animals), and RDC (179 animals)] from the 1000 Bull Genomes Project [22]. The parameters used for the identification of HERs in all breeds were:

- Minimum number of SNPs in a HER equals to 10;
- Maximum distance between two consecutive SNPs (GAP, in Kb) equals to 500 Kb;
- Minimum length of a HER (Kb) equals to 10 Kb; and,
- The maximum number of homozygous genotypes allowed in a HER equals to two.

This combination of parameters was chosen for their ability to effectively capture HERs, as demonstrated in the previous analyses. Figure 1 presents a summary of all the analyses performed.

## Results

### Impact of the parameters used on HER identification

**Fig. 2:**
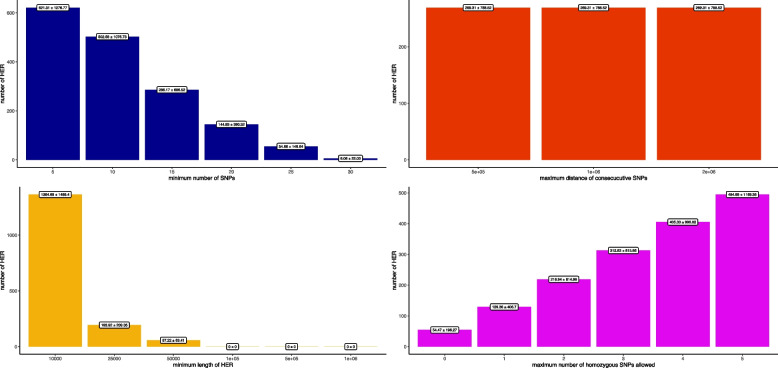
Effect of parameters on the detection of heterozygous-enriched regions

The effect of the parameters investigated on the number of HERs identified based on WGS data is reported in Fig. 2. The maximum distance between consecutive SNPs did not impact the average number of HERs detected. In contrast, increasing the minimum number of SNP in a HER decreased the number of HERs from 621.31(± 1,276.77), when considering 5 SNPs, to 6.08(± 6.08), when considering 30 SNPs. The same pattern was observed for the minimum length of the HERs, where a decrease in the number of detected HERs was observed when increasing the minimum length size of a HER, with a higher detection when the parameter was equal to 10 Kb. At a minimum HER length of 10 Kb, the corresponding increase in the number of HERs was 603% and 2,283% compared to HER minimum sizes of 25 Kb and 50 Kb, respectively. The minimum length size higher than 100 Kb did not detect HERs, as shown in Fig. 2.

When considering the maximum number of homozygous markers allowed inside a HER, the pattern was the opposite compared to the minimum number of markers and length size of a HER. As expected, more HERs were observed when more homozygous markers were allowed in a HER. The range for this parameter was on average 54.47 (± 196.27) numbers of HER, when allowing 0 homozygous markers, to 494.89 (± 1,169.35), when allowing 5 homozygous markers, an average increase of 808% on the number of detected HERs. A similar pattern was observed in the HD SNP panel results for all the parameters. However, the minimum length of a HER in the 50 K and 100 K SNP panels exhibited a greater variation across the selected sizes, ranging from 16,542.94 (± 34,229.63) HERs detected for a length of 10 Kb to 102.97 (± 194.07) for a length of 1,000 Kb in the 50 K panel, and from 35,365.22 (± 60,859.43) for a length of 10 Kb to 93.36 (± 111.62) for a length of 1,000 Kb in the 100 K panel. The effect of the parameters on the commercial panels is reported in Additional file 1 Figure S1 to Additional file 3 Figure S3, and the number of HERs overlapping between SNP panels and WGS for each parameter combination is reported in Additional file 4 Table S1 to Additional file 6 Table S3.

**Fig. 3:**
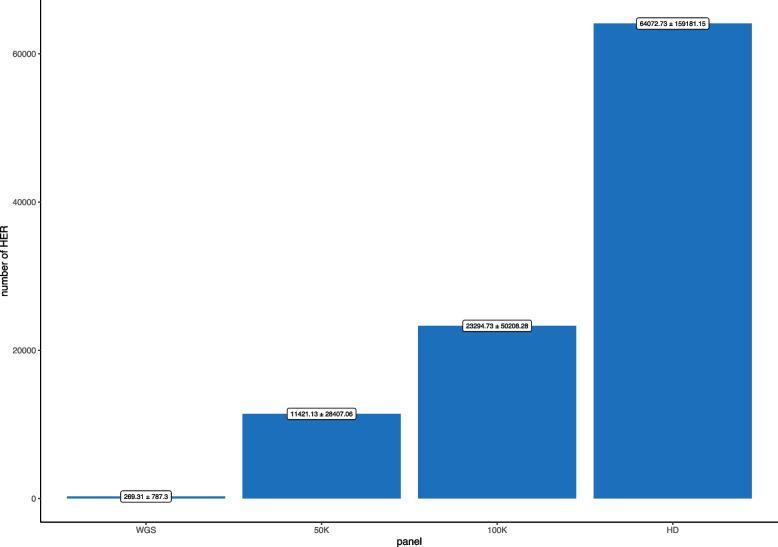
Average and standard deviation of heterozygous-enriched regions detected by whole genome sequence (WGS) and commercial SNP panel

The average number of HERs detected based on each of the lower density SNP panels evaluated is illustrated in Fig. 3. The number of HERs identified based on SNP panels was higher than the one observed based on WGS data and increased from the 50 K (11,421.13 ± 28,407.06) to the HD panel (64,072.73 ± 159,181.15), with a difference of 4,141%, 8,550%, 23,692%, for 50, 100 K, and HD respectively, in comparison to the WGS data results.

### Classification and distribution of HERs across the genome

There is no unique metric for the minimum number of markers or the number of homozygous markers allowed inside HERs that show a high differentiation in the HER call. As such, a minimum number of markers equal to 5 and a maximum number of homozygous ranging from 0 to 2, and a minimum number of markers equals 10, and the maximum homozygous ranging from 0 to 5 were chosen to assess the distribution of HER across the genome, as well the classification based on the length size of a HER. For all these new scenarios, created in step #2 (Fig. 1), the minimum length was hold at 10 Kb, as this value showed a substantial difference in the number of HERs identified, in comparison to the others length sizes evaluated.

Table 1 shows the classification of HERs based on the length size for the scenarios with minimum number of markers equal to 5 and 10. The number of HERs detected increased with the number of homozygous markers allowed in both scenarios. In the scenario considering the minimum number of markers equal to 5, the number of HERs ranged from 36,024 to 99,811, with the number of homozygous markers equal to 0 to 2, respectively. In scenario 10, the range was 17,942 to 115,568 HER detected with the maximum number of homozygous markers ranging from 0 to 5, respectively. Most HER detected in all scenarios were classified as 10–20 Kb, representing 63.3% to 75.1% of the total number of HERs in scenario 5 and 53.4% to 72.0% in scenario 10.

**Table 1:** Number of heterozygous-enriched regions (HERs) detected and classified based on the length size for the scenarios with minimum number of markers equals to 5 and 10

Length of HER	5_0	5_1	5_2	10_0	10_1	10_2	10_3	10_4	10_5
10-20 Kb	23,888	49,777	74,945	9,578	22,895	39,236	55,166	69,741	83,251
20-30 Kb	2,426	6,396	11,624	1,131	3,007	6,059	10,015	14,941	20,015
30-40 Kb	1,443	2,301	3,415	730	1,255	1,780	2,222	2,765	3,535
40-50 Kb	901	1,349	1,653	613	992	1,348	1,731	2,034	2,201
> 50 Kb	7,366	8,130	8,174	5,890	6,821	7,471	7,381	6,998	6,566
Total	36,024	67,953	99,811	17,942	34,970	55,894	76,515	96,479	115,568

**Fig. 4:**
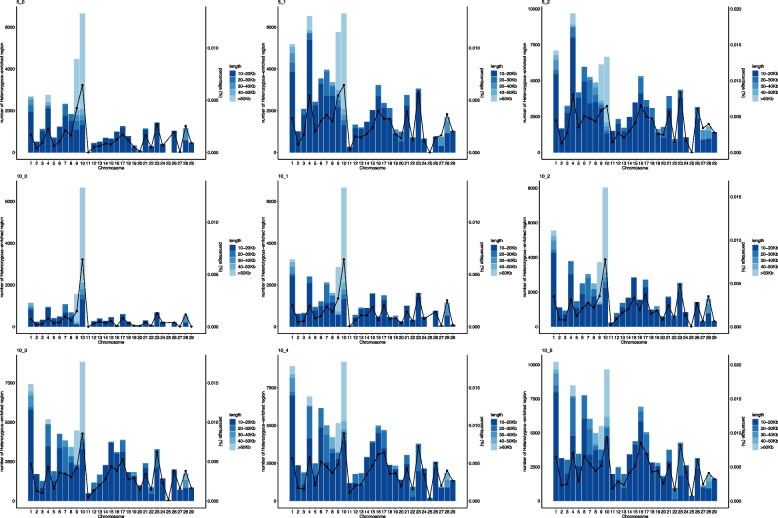
Classification of heterozygous-enriched regions (HER), by chromosome, according to length and the average percentage of chromosome coverage by HERs in each of the scenarios with minimum number of the marker in a HER equals to 5 and 10 and number of homozygous markers allowed inside of a HER equals to 0 to 5

5_0: scenario with minimum number of markers equal 5 and 0 homozygous allowed inside of a HER;

5_1: scenario with minimum number of markers equal 5 and 1 homozygous allowed inside of a HER;

5_2: scenario with minimum number of markers equal 5 and 2 homozygous allowed inside of a HER;

10_0: scenario with minimum number of markers equal 10 and 0 homozygous allowed inside of a HER;

10_1: scenario with minimum number of markers equal 10 and 1 homozygous allowed inside of a HER;

10_2: scenario with minimum number of markers equal 10 and 2 homozygous allowed inside of a HER;

10_3: scenario with minimum number of markers equal 10 and 3 homozygous allowed inside of a HER;

10_4: scenario with minimum number of markers equal 10 and 4 homozygous allowed inside of a HER;

10_5: scenario with minimum number of markers equal 10 and 5 homozygous allowed inside of a HER;

Figure 4 shows the HER classification by chromosome and the percentage of genome coverage by HERs. BTA10 showed the highest concentration of HERs of large size (> 50 Kb). In addition, BTA10 showed more HERs in scenarios with minimum number of markers equal to 5, with maximum number of homozygous 0 and 1, and the minimum number of markers equal to 10, with maximum number of homozygous 0 to 4. Regarding genome coverage, the percentage of HERs did not exceed 0.01%, with BTA10 showing the highest rate.

### Correlations

**Fig. 5:**
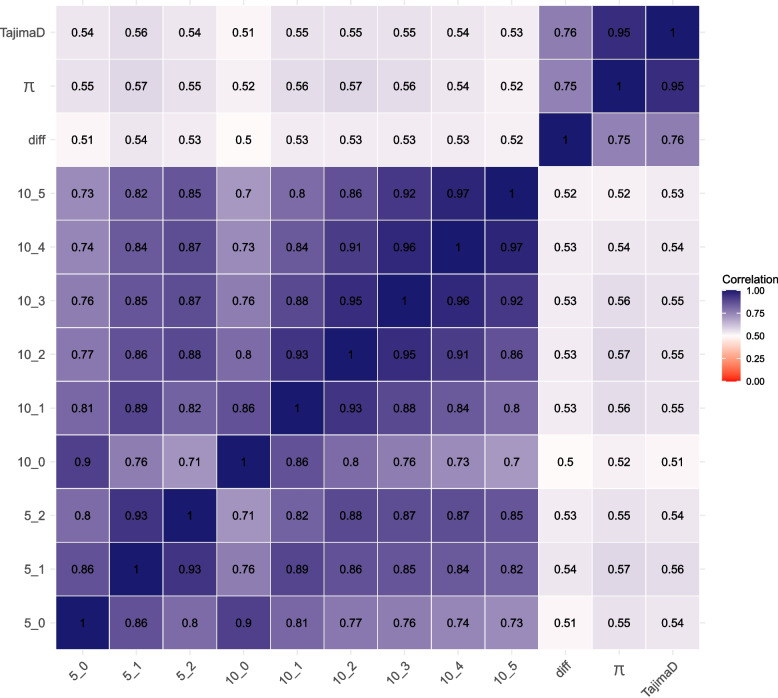
Spearman correlation among scenarios with a minimum number of the marker inside of HER equals to 5 and 10 and a number of homozygous markers allowed inside of a HER from 0 to 5, and differences based on heterozygosity observed and expected, diversity of nucleotide (π) and Tajima’s D statistic

The Spearman correlation among scenarios ranged from moderate to high, considering the proportion of times that the SNP appears inside a HER. The rank correlation was considered moderate between all scenarios and between the diversity metric estimated (expected and observed heterozygosity, nucleotide diversity, and Tajima’s D test). The overall Spearman correlations are presented in Fig. 5. Additional file 7 Figure S4 provides the Spearman correlations among scenarios and diversity nucleotide metrics per chromosome.

### Heterozygous-enriched region islands, linkage disequilibrium, and functional analyses

**Fig. 6:**
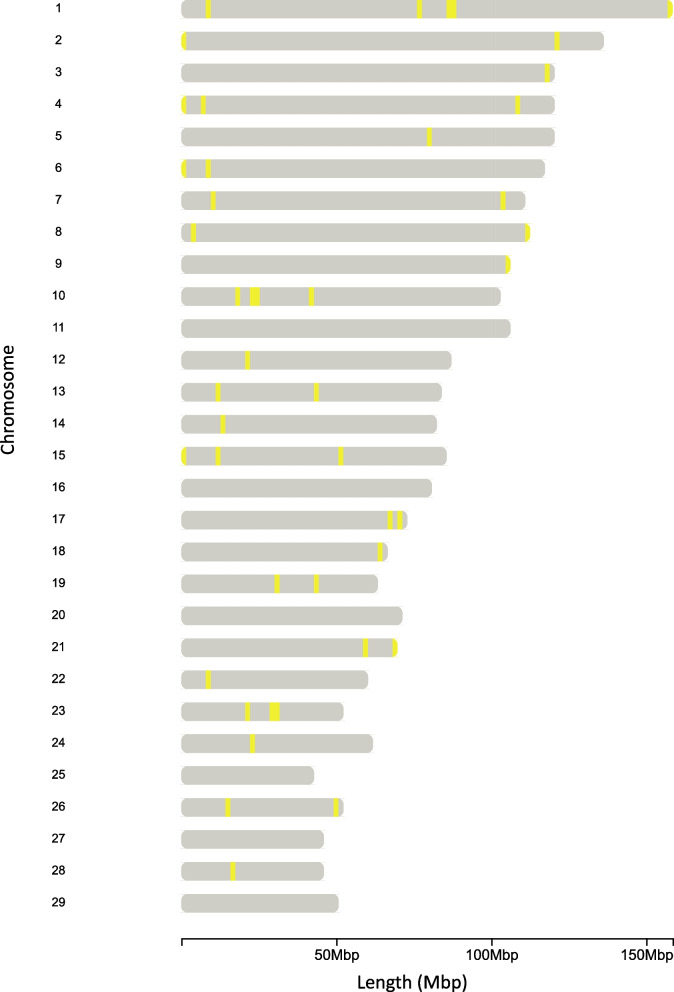
Distribution of heterozygous-enriched region islands across the Holstein cattle genome

The overlapped of the HER islands across all nine scenarios created in step #2 (Fig. 1) are presented in Fig. 6. Forty-five islands with markers with high Tajima’s D values and high differential of observed and expected heterozygosity were found across all scenarios evaluated. These HER islands were located on 23 chromosomes and contained 3,440 markers. The percentage that each island appears in each of the scenarios and the average linkage disequilibrium between the markers located within HERs are presented in Table 2.

**Table 2:** Percentage of heterozygous-enriched regions (HERs) islands observed in all scenarios and the average of linkage disequilibrium (r^2^) of markers inside HER islands in Holstein cattle

CHR	START	END	NSNP	r^2^	5_0	5_1	5_2	10_0	10_1	10_2	10_3	10_4	10_5
BTA1	8,947,612	8,984,143	54	0.010	10.14	35.97	61.94	10.11	32.86	58.74	77.79	88.23	94.93
BTA1	77,541,594	77,574,520	114	0.143	24.20	48.00	67.19	17.75	41.52	64.06	74.97	78.78	84.88
BTA1	86,455,167	86,476,612	40	0.062	10.00	10.00	14.77	10.00	10.00	12.34	23.67	32.36	32.87
BTA1	88,572,745	88,595,480	149	0.021	10.00	10.00	10.00	10.00	10.00	10.00	12.92	19.51	25.28
BTA1	158,026,308	158,204,765	2064	0.173	46.31	75.37	87.27	18.18	45.38	70.30	85.03	91.26	93.40
BTA2	11,729	23,106	13	0.001	10.00	10.95	26.02	10.00	10.95	26.02	43.45	55.63	63.23
BTA2	121,358,897	121,376,671	23	0.010	33.05	56.62	70.09	17.84	41.76	62.04	73.44	76.09	75.48
BTA3	119,398,854	119,418,253	22	0.004	34.63	56.51	71.32	24.38	49.64	69.09	80.00	78.71	76.62
BTA4	114,921	131,012	29	0.001	11.17	40.27	70.22	11.17	40.27	70.22	88.15	94.99	98.03
BTA4	7,748,093	7,786,109	61	0.001	39.61	68.96	83.21	27.85	55.59	73.49	83.41	88.04	92.01
BTA4	108,267,668	108,297,255	35	0.004	28.34	57.05	64.85	21.52	50.88	63.06	72.90	82.38	90.95
BTA5	79,520,323	79,544,963	23	0.002	39.11	71.75	89.56	28.25	57.91	80.25	91.35	96.19	98.20
BTA6	39,744	67,663	32	0.003	32.00	46.50	69.91	17.76	31.63	60.84	84.15	95.38	95.93
BTA6	8,997,519	9,019,722	29	0.014	19.78	38.10	56.98	19.57	37.63	56.89	71.05	83.75	89.99
BTA7	9,986,201	9,996,885	10	0.133	53.60	84.25	91.97	10.22	28.68	48.38	69.13	81.96	90.93
BTA7	104,258,582	104,281,037	14	0.001	49.34	79.67	92.09	24.80	55.07	80.54	92.39	95.66	97.55
BTA8	4,462,538	4,479,090	20	0.001	38.93	60.33	70.98	19.52	44.73	63.41	78.73	90.41	96.83
BTA8	111,993,409	112,004,112	16	0.001	14.86	39.06	66.67	14.86	37.75	64.39	85.79	96.15	88.00
BTA9	104,119,378	104,141,024	13	0.002	29.58	63.91	84.23	10.32	35.01	64.23	84.81	95.70	98.46
BTA10	18,852,596	18,940,485	99	0.101	23.20	63.54	75.32	14.38	49.14	75.32	89.89	96.52	98.24
BTA10	23,775,405	24,071,948	47	0.009	67.00	87.97	88.53	42.16	72.19	88.53	94.83	96.73	97.42
BTA10	24,100,254	24,459,318	44	0.001	68.02	88.26	88.46	43.29	72.75	88.46	94.40	96.17	96.98
BTA10	42,169,769	42,201,656	32	0.002	29.98	57.85	74.20	20.15	50.29	74.20	79.39	74.09	76.30
BTA12	21,476,167	21,501,639	42	0.006	25.52	49.65	66.89	21.92	45.33	63.26	77.92	84.18	89.73
BTA13	11,310,352	11,334,273	40	0.028	22.71	44.06	58.87	21.32	42.72	58.24	70.72	77.30	81.55
BTA13	43,331,311	43,358,950	22	0.003	15.59	51.88	81.02	10.29	37.05	67.87	86.79	95.53	96.96
BTA14	13,489,163	13,506,285	13	0.001	47.68	74.43	90.80	21.87	56.03	81.83	81.15	89.10	95.45
BTA15	7,225	21,935	40	0.002	13.54	40.19	65.25	13.05	39.08	64.56	79.05	72.18	63.03
BTA15	12,265,252	12,276,097	11	0.002	33.28	68.21	88.58	21.58	57.92	82.08	93.63	66.95	77.78
BTA15	51,457,087	51,470,355	15	0.002	10.00	30.59	54.46	10.00	28.37	52.39	65.55	76.03	81.23
BTA17	68,057,715	68,070,742	11	0.001	32.07	52.70	71.96	16.49	39.07	63.25	81.00	92.69	88.78
BTA17	71,143,576	71,169,181	36	0.001	36.07	59.83	78.10	27.79	56.11	77.38	90.09	95.91	97.75
BTA18	63,626,958	63,643,160	17	0.001	22.49	38.40	58.38	22.49	38.40	58.38	83.92	84.09	90.50
BTA19	31,258,759	31,290,592	27	0.002	10.00	12.64	21.25	10.00	10.66	20.16	33.49	48.13	61.26
BTA19	43,263,869	43,277,164	10	0.001	19.34	53.57	79.55	10.00	21.47	56.75	81.73	91.62	94.29
BTA21	58,941,106	58,962,580	11	0.007	20.53	49.73	68.81	10.00	30.53	55.44	76.78	87.90	91.34
BTA21	69,831,448	69,844,336	18	0.039	29.53	63.65	84.69	18.87	51.74	78.87	91.47	91.19	93.04
BTA22	8,699,801	8,723,909	18	0.001	12.38	40.16	65.00	10.00	25.38	53.58	76.12	85.55	89.24
BTA23	21,755,400	21,781,579	23	0.191	31.61	54.95	75.63	25.04	40.82	63.21	86.17	94.46	95.79
BTA23	30,203,633	30,220,658	20	0.001	36.33	73.85	89.66	21.46	57.35	81.93	91.83	94.93	96.25
BTA23	30,303,856	30,326,074	25	0.001	19.08	49.33	71.44	13.90	36.24	62.50	81.51	89.13	93.70
BTA24	22,868,170	22,890,804	36	0.035	26.95	47.28	63.89	18.59	38.13	59.57	68.92	80.36	85.13
BTA26	15,215,724	15,230,752	13	0.001	32.61	43.57	84.13	15.28	43.57	71.63	88.80	95.25	97.08
BTA26	50,115,341	50,125,621	11	0.015	10.00	24.67	55.64	10.00	24.67	53.02	78.12	83.11	85.62
BTA28	16,525,956	16,567,307	16	0.001	44.52	66.83	79.83	28.34	55.69	76.22	89.45	93.82	95.44

CHR: chromosome;

START: base-pair position where the heterozygous-enriched region island starts;

END: base-pair position where the heterozygous-enriched region island ends;

NSPN: number of markers inside of the heterozygous-enriched region;

5_0: scenario with minimum number of markers equal 5 and 0 homozygous allowed inside of a HER;

5_1: scenario with minimum number of markers equal 5 and 1 homozygous allowed inside of a HER;

5_2: scenario with minimum number of markers equal 5 and 2 homozygous allowed inside of a HER;

10_0: scenario with minimum number of markers equal 10 and 0 homozygous allowed inside of a HER;

10_1: scenario with minimum number of markers equal 10 and 1 homozygous allowed inside of a HER;

10_2: scenario with minimum number of markers equal 10 and 2 homozygous allowed inside of a HER;

10_3: scenario with minimum number of markers equal 10 and 3 homozygous allowed inside of a HER;

10_4: scenario with minimum number of markers equal 10 and 4 homozygous allowed inside of a HER;

10_5: scenario with minimum number of markers equal 10 and 5 homozygous allowed inside of a HER;

The largest HER island was identified on BTA10, in the region between 24,100,254 bp to 24,459,318 bp, with a length of 359.06 Kb. The smallest HER island was identified on BTA26, in the regions between 50,115,341 bp to 50,125,621 bp. The average length size of the HER islands was 40.06 Kb (± 68.37 Kb). The level of linkage disequilibrium was generally small in all HER islands evaluated, ranging from 0.001 to 0.191. One particular HER island (BTA23:21,755,400 –21,781,579 bp) shows part of the markers with strong linkage disequilibrium, as shown in Fig. 7. This region is responsible for coding the gene *ENSBTAG00000054235;* a gene previously linked with paratuberculosis infection in cattle [35]. Additional file 8 Figure S5 presents the heatmap for all the HER islands found.

**Fig. 7:**
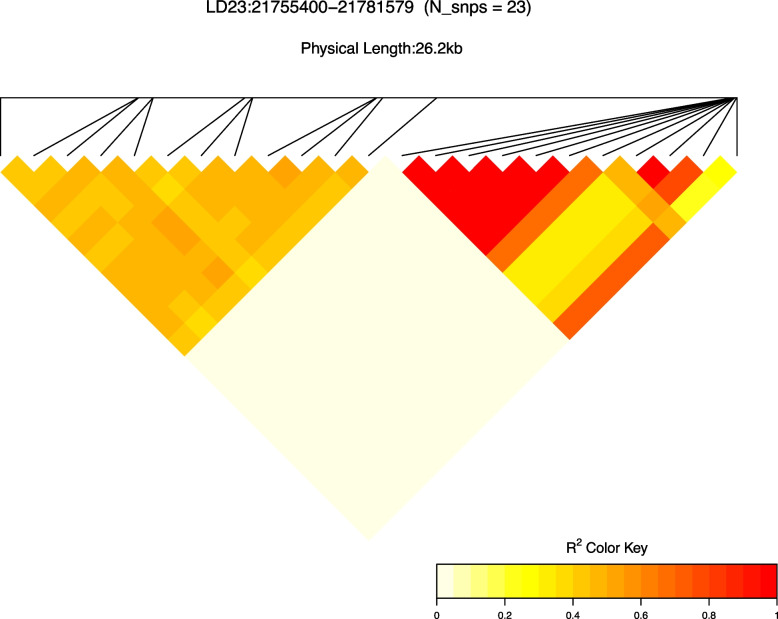
Heatmap of linkage disequilibrium for the heterozygous-enriched region islands located on chromosome 23

Table 3 presents the gene annotation for the regions in HER islands. The significant (*p* < 0.05) biological processes, molecular functions, cellular components, and metabolic pathways for the genes found in the HER islands are presented in Table 4.

**Table 3:** Gene annotation for the Heterozygous enriched regions (HER) islands

CHR	START	END	Length	Gene ID	Gene name	Gene biotype
BTA1	77,541,594	77,574,520	32,926	ENSBTAG00000015460	*TP63*	Protein coding
BTA1	8,947,612	8,984,143	36,531	ENSBTAG00000052332	*H4C3*	Protein coding
BTA1	158,026,308	158,204,765	178,457	ENSBTAG00000053325		Protein coding
BTA1	158,026,308	158,204,765	178,457	ENSBTAG00000049601	*OR2B28*	Protein coding
BTA1	158,026,308	158,204,765	178,457	ENSBTAG00000037965	*ZSCAN23*	Protein coding
BTA1	158,026,308	158,204,765	178,457	ENSBTAG00000050787	*5S_rRNA*	rRNA
BTA4	7,748,093	7,786,109	38,016	ENSBTAG00000050410		pseudogene
BTA6	39,744	67,663	27,919	ENSBTAG00000048454	*U6*	snRNA
BTA8	111,993,409	112,004,112	10,703	ENSBTAG00000003540	*MYT1L*	Protein coding
BTA10	18,852,596	18,940,485	87,889	ENSBTAG00000007433	*MYO9A*	Protein coding
BTA10	18,852,596	18,940,485	87,889	ENSBTAG00000052861	*bta-mir-2285dh*	miRNA
BTA12	21,476,167	21,501,639	25,472	ENSBTAG00000019134	*NEK5*	Protein coding
BTA13	11,310,352	11,334,273	23,921	ENSBTAG00000051963		Protein coding
BTA17	71,143,576	71,169,181	25,605	ENSBTAG00000017305	*IG_C_gene*	Protein coding
BTA19	43,263,869	43,277,164	13,295	ENSBTAG00000049347		Protein coding
BTA19	43,263,869	43,277,164	13,295	ENSBTAG00000049357	*U2*	snRNA
BTA19	43,263,869	43,277,164	13,295	ENSBTAG00000045620	*U2*	snRNA
BTA19	31,258,759	31,290,592	31,833	ENSBTAG00000021938	*ARHGAP44*	Protein coding
BTA21	58,941,106	58,962,580	21,474	ENSBTAG00000039808	*SERPINA6*	Protein coding
BTA23	21,755,400	21,781,579	26,179	ENSBTAG00000054235		Protein coding
BTA23	30,203,633	30,220,658	17,025	ENSBTAG00000051232		Protein coding
BTA23	30,303,856	30,326,074	22,218	ENSBTAG00000051232		Protein coding
BTA23	30,203,633	30,220,658	17,025	ENSBTAG00000051628		Protein coding
BTA23	30,303,856	30,326,074	22,218	ENSBTAG00000008943	*ZSCAN12*	Protein coding
BTA24	22,868,170	22,890,804	22,634	ENSBTAG00000010299	*NOL4*	Protein coding
BTA26	15,215,724	15,230,752	15,028	ENSBTAG00000055018		pseudogene
BTA28	16,525,956	16,567,307	41,351	ENSBTAG00000010109	*CDK1*	Protein coding
BTA28	16,525,956	16,567,307	41,351	ENSBTAG00000035206		Protein coding

**Table 4:** Significant (*p* < 0.05) Gene Ontology (GO) terms and pathways for the genes located within heterozygous-enriched regions (HER) islands

GeneSet	Description	P-value	Gene ID
Biological Processes			
GO:0051301	Cell division	2.5 × 10^–03^	*ENSBTAG00000015460; ENSBTAG00000010109*
GO:0030855	Epithelial cell differentiation	3.2 × 10^–03^	*ENSBTAG00000015460; ENSBTAG00000010109*
GO:0030162	Regulation of proteolysis	5.2 × 10^–03^	*ENSBTAG00000015460; ENSBTAG00000039808*
GO:0006325	Chromatin organization	5.6 × 10^–03^	*ENSBTAG00000015460; ENSBTAG00000010109*
GO:0006974	Cellular response to DNA damage stimulus	6.1 × 10^–03^	*ENSBTAG00000015460; ENSBTAG00000010109*
GO:0007164	Establishment of tissue polarity	7.0 × 10^–03^	*ENSBTAG00000015460*
GO:0048483	Autonomic nervous system development	7.6 × 10^–03^	*ENSBTAG00000015460*
GO:1,904,888	Cranial skeletal system development	9.6 × 10^–03^	*ENSBTAG00000015460*
GO:0030104	Water homeostasis	1.1 × 10^–02^	*ENSBTAG00000015460*
GO:0042303	Molting cycle	1.7 × 10^–02^	*ENSBTAG00000015460*
GO:0055123	Digestive system development	2.0 × 10^–02^	*ENSBTAG00000015460*
GO:0007498	Mesoderm development	2.1 × 10^–02^	*ENSBTAG00000015460*
GO:0018210	Peptidyl-threonine modification	2.1 × 10^–02^	*ENSBTAG00000010109*
GO:0007568	Aging	2.4 × 10^–02^	*ENSBTAG00000015460*
GO:0072331	Signal transduction by p53 class mediator	2.4 × 10^–02^	*ENSBTAG00000015460*
GO:0098727	Maintenance of cell number	2.5 × 10^–02^	*ENSBTAG00000015460*
GO:0048736	Appendage development	2.8 × 10^–02^	*ENSBTAG00000015460*
GO:0007219	Notch signaling pathway	3.3 × 10^–02^	*ENSBTAG00000015460*
GO:0048863	Stem cell differentiation	3.5 × 10^–02^	*ENSBTAG00000015460*
GO:0001763	Morphogenesis of a branching structure	3.5 × 10^–02^	*ENSBTAG00000015460*
GO:0043588	Skin development	3.8 × 10^–02^	*ENSBTAG00000015460*
GO:0030522	Intracellular receptor signaling pathway	4.0 × 10^–02^	*ENSBTAG00000015460*
GO:0070997	Neuron death	4.3 × 10^–02^	*ENSBTAG00000015460*
GO:0008544	Epidermis development	4.3 × 10^–02^	*ENSBTAG00000015460*
GO:0008202	Steroid metabolic process	4.7 × 10^–02^	*ENSBTAG00000039808*
Molecular Functions			
GO:0003682	Chromatin binding	3.4 × 10^–03^	*ENSBTAG00000015460; ENSBTAG00000010109*
GO:0002039	p53 binding	1.2 × 10^–02^	*ENSBTAG00000015460*
GO:0003684	Damaged DNA binding	1.5 × 10^–02^	*ENSBTAG00000015460*
GO:0031072	Heat shock protein binding	2.4 × 10^–02^	*ENSBTAG00000010109*
GO:0061134	Peptidase regulator activity	5.0 × 10^–02^	*ENSBTAG00000039808*
Cellular Components			
GO:0030496	Midbody	4.7 × 10^–02^	*ENSBTAG00000010109*
Pathways			
bta04115	p53 signaling pathway	1.8 × 10^–02^	*ENSBTAG00000010109*
bta04914	Progesterone-mediated oocyte maturation	2.1 × 10^–02^	*ENSBTAG00000010109*
bta04540	Gap junction	2.2 × 10^–02^	*ENSBTAG00000010109*
bta04114	Oocyte meiosis	2.7 × 10^–02^	*ENSBTAG00000010109*
bta04110	Cell cycle	2.9 × 10^–02^	*ENSBTAG00000010109*
bta04218	Cellular senescence	4.0 × 10^–02^	*ENSBTAG00000010109*
bta05168	Herpes simplex infection	4.7 × 10^–02^	*ENSBTAG00000010109*

For the QTL enrichment analyses, 31 regions related to 11 traits were identified. Of those, 40% are related to milk, 30% to production,13.33% to the exterior, 10% to health, and 6.67% to reproduction. The QTL enrichment results are presented in Table 5.

**Table 5:** Significant (*p* < 0.05) QTL (quantitative trait loci) for the genes located within heterozygous-enriched regions (HER) islands

QTL	CHR	N_QTLs	*P*-value	Adjusted *P*-value	QTL type
Metabolic body weight	BTA1	8	3.6 × 10^–12^	4.0 × 10^–11^	Production
Milk kappa-casein percentage	BTA1	8	1.3 × 10^–04^	6.9 × 10^–04^	Milk
Milk unglycosylated kappa-casein percentage	BTA1	4	3.1 × 10^–02^	3.8 × 10^–02^	Milk
Clinical mastitis	BTA6	2	1.4 × 10^–03^	3.7 × 10^–03^	Health
Udder cleft	BTA6	1	7.0 × 10^–03^	1.3 × 10^–02^	Exterior
Udder depth	BTA6	1	1.5 × 10^–02^	2.1 × 10^–02^	Exterior
Udder height	BTA6	1	8.8 × 10^–03^	1.4 × 10^–02^	Exterior
Udder structure	BTA6	1	2.9 × 10^–04^	1.1 × 10^–03^	Exterior
*M. paratuberculosis* susceptibility	BTA10	1	6.6 × 10^–03^	1.3 × 10^–02^	Health

### Comparison among breeds

The number of HER islands found for each breed, the number of islands in common with HOL, the specific regions for the breed and HOL, and the gene set for the specific region in HOL are presented in Table 6.

**Table 6:** Comparison of heterozygous-enriched region (HER) islands among Holstein (HOL), Angus (ANG), Jersey (JER), and Norwegian red cattle (RDC)

Breed	n island	% in common	n specific islands	Breed-specific HER islands	Holstein-specific HER islands
JER	68	94.28	2	BTA4:7,754,341–7,779,125; BTA16: 4,805,929–4,981,727	BTA11: 29,406–32,150; BTA16: 48,405,929–49,810,727; BTA17: 30,241,885–30,254,164; BTA24: 46,608,950–46,619,501
ANG	72	95.72	4	BTA10: 8,947,684–8,979,955; BTA19: 566,224,233–566,316,890; BTA22: 86,844,336–87,184,460; BTA29: 48,220,837–48,234,421	BTA1: 8,947,673–8,982,695; BTA19: 56,624,233–56,631,689; BTA22: 8,699,807–8,723,909
RDC	72	97.14	2	BTA4: 7,756,346–7,779,125; BTA18: 5,717,899–5,727,914	BTA10: 10,036,762–10,051,565; BTA14: 49,089,801–55,444,216

The breed with the highest number of overlapped HER islands was NRC, with 97.14% of the same islands found in HOL cattle. JER had the smallest percentage of HER islands in common with the HOL breed (94.28%), followed by ANG (95.72%). The genes related to each one of the particular breed HER islands are presented in Additional file 9 Table S4.

## Discussion

### Impact of the parameters used in the identification of HERs

We first evaluated the impact of different parameters on the identification of HERs and HER islands. As presented in Fig. 2, three parameters (i.e., the minimum number of markers, minimum length of a HER, and the maximum number of homozygous markers) significantly influenced the detection of HERs, and the minimum length of 10 Kb enabled the detection of most HERs based on WGS. This corroborates the assumption that HERs are small regions scattered through the genome that randomly occur due to different population processes (e.g., selection, mutation, migration) [12]. This finding directly impacts the choice of the SNP panels to be used for these analyses as the accurate detection of shorter HERs across the genome requires denser SNP panels [36].

The high number of markers in the WGS dataset enabled the identification of small informative HERs across the genome. Therefore, the use of WGS is a good alternative, because the majority of HERs are small DNA stretches throughout the genome (Table 1 and Fig. 4). Another point to be highlighted, as shown by Ceballos et al. [36] is that the total number of heterozygous markers present in WGS is higher than that of SNP panels. Therefore, the chances of capturing HERs are higher when using WGS data than commercial panels.

SNP panels are usually designed based on SNPs present with a frequency of more than 1% in the population used for its design [37]. These polymorphisms typically have higher allele frequency and are located near QTL associated with important traits [38]. The genome scan with such SNP panels assumes that markers not located between two consecutive heterozygous SNP are heterozygous. Therefore, ascertainment bias in the information provided could exist, mainly in the length of a HER. Small-length HERs might not be detected, the length size might be inflated, or yet false-positive HERs might be identified.

As presented in Fig. 3, a substantially inflated number of HERs was obtained when using SNP panels compared to WGS. Interestingly, the higher density (100 K and HD) SNP panels enabled a greater detection rate of HERs. In addition, the larger HERs identified in less dense SNP panels might be multiple HERs side by side in denser panels. As the chances of detecting false-positive or false-negative HERs in SNP panels are higher, additional analyses, such as nucleotide diversity and/or Tajima D statistics, can be complementary metrics to validate the detected HERs.

It seems there is a pattern between the number of HERs found by chromosome and the diversity metrics. Although the overall rank correlation was moderated (Fig. 5), the correlation was higher for chromosomes with a larger number of HERs (Additional file 8 Figure S5 – case of BTA9 and BTA10). This suggests that for chromosomes with a higher number of HERs, and longer HERs, the combination of parameters in the additional scenarios captured the markers in higher diversity in the population, besides the difference between the minimum number of markers that constitute a HER and the number of homozygous allowed inside of a HER.

Regarding the minimum number of markers that make up a HER and the maximum number of homozygous markers allowed inside a HER, these parameters seem more related to how conservative the analyses are. Both parameters work in the opposite direction and have higher impact on detecting HERs. The choice of these parameters needs to consider previous information about the population, selection history, density of SNP panels, and genotyping quality. In populations under a long direct selection process, with small diversity among the individuals, and a small effective population size, the autozygosity levels in these populations are expected to be high [21, 39]. In response, more flexible parameters could be used to detect HERs in these populations. For instance, allowing more homozygous markers inside of HERs could be a way to consider possible calling errors that may wrongly break the sequence of heterozygous regions affected by the genotyping quality [36]. Therefore, the decision to allow or not for more lenient parameters in the analyses depends on the quality of the SNP panels or other DNA genotyping or sequencing platform used.

### Heterozygous-enriched region islands

Heterozygous-enriched regions can appear throughout the genome, but when they are concentrated in a specific region, this could indicate a pattern of selection events in the population [40]. Forty-five islands were found in all scenarios, and all of them had a positive Tajima’s D value indicating selection for heterozygous genotypes (balancing selection). The Tajima’s D test considers the nucleotide diversity (π) and an expectation for π based on the average pairwise markers plus the total number of mutations [41]. As a result, if the values are positive, the marker is under balancing selection [30]. Here, we used such analysis as validation to confirm that the markers inside of a HER had been selected over the generations and endorse the potential island of HERs.

Regarding the linkage disequilibrium among markers inside a HER, most markers have shown a low linkage disequilibrium. Comparing our results to those from Qanbari and Wittenburg [42], part of the HER islands found in our study are located in regions defined as recombination hotspot intervals, where the recombination rate exceeds 2.5 standard deviations from the genome-wide average recombination rate. Such recombination breaks and recombines different alleles. In this process, new HERs are created mainly in HERs near telomere regions. However, this does not seem to be the only process that leads to the creation of HER islands. Twenty-eight genes and 11 QTLs were founded related to the HER islands in HOL (Tables 3 and 5) and could be, in some way, associated with a heterozygous advantage. Some of the genes found in these regions are related to immune response, as expected once higher heterozygosity levels can lead to greater infectious disease resistance [43]. On the other hand, many genes were found to be related to traits such as fertility and production. For instance, TP63 or tumor protein p63, which has a function of binding certain regions of DNA and controlling the activities of particular genes, was previously associated with puberty in cattle [44]. The *ZSCAN23* gene is involved in transcription’s regulation of RNA polymerase III and has been associated with male fertility [44]. The *ARHGAP44* gene, which affects cell polarity, vesicular trafficking, cell cycle, and transcription, has also been reported to influence cow fertility [45].

The *TP63* and *CDK1* genes are related to a higher number of processes and pathways, as shown in Table 4. Such genes impact mechanisms that control the process of multiplication of cells and/or transcription of the genes and are involved in multiple gene ontological terms. Other traits related to genes found in HER islands include milk fatty acids and milk fat percentage (e.g., *MYT1L*, [46]); residual feed intake (e.g., *MAYO9A* [47]); vitrification temperature of mature bovine oocytes (e.g., *NEK5* [48]); and, hormone homeostasis and levels of progesterone (e.g., *SERPINA6* [49]). Some of these traits, although are under positive selection processes, could still be present within a HER due to certain processes such as increased diversity around a target selection or non-synonymous polymorphisms segregating at intermediate frequencies [19]. These processes can lead to concentration of heterozygous alleles around these regions and increase in the number of polymorphic markers around them.

Regarding the comparison among breeds, at least 94.28% of the HER islands in HOL are present in the other three breeds evaluated. Interestingly, particularities such as the ancestry among the breed (as in HOL and NRC) or distinctive selection processes (HOL and ANG) seem not to show an impact on the presence of common HERs across the breeds. Although the process that contributes to the presence of the heterozygosity in the population is known, the persistence of such heterozygosity in areas of the genome is still not completely uncovered [11]. The most acceptable reason for this persistence the heterozygote advantage [19] is likely linked to the fitness/survival traits that are related to evolutionary process associated with animal adaptation [11, 15].

### Limitations and implications

In this study, we applied different parameter combinations for the identification of HERs, which is the first study to assess the impact of various combinations of different parameters on HER detection. This study provides background information for the design of future HER studies to better understand their role in biological mechanisms and evolutive processes. Our results show that HERs are small regions spread across the genome and likely concentrated in genomic regions under balancing selecting pressure. Although it is difficult to determine what are the “true” HERs on the genome when using real datasets (instead of simulated datasets), they provide important insights about the different selection forces that the population may have been through. The use of WGS data for HERs detection studies is the most recommended. Our results show that SNP panels resulted in an inflated number of HERs and applying additional metrics that contribute to decrease such inflation is recommended.

Here, the minimum length to capture more HERs was 10 Kb and, although neither the minimum number of markers nor the maximum number of homozygous markers allowed inside of a HER showed a distinct difference, we observed better identification of HERs with the minimum number of markers equals to 10 and a maximum number of homozygous ranging from 0 to 3. These parameter values seem to be adequate for capturing the relevant HERs, decreasing the capture of noises, and, more importantly, capturing regions with a concentration of HER islands. Interestingly, all the HER islands found had a minimum number of markers equal to or higher than 10, which reinforces the use of such level for the minimum number of markers. Regarding, the level assumed to declare if a region is located in a HER island (> 10%), we understand that it could be considered a low level. A metric that confirms whether the region has been or is not selected should also be applied. In our study, all the islands found showed a Tajima’s D value higher than zero, indicating that such regions on the genome had been selected for higher heterozygosity. Future studies utilizing simulated datasets are recommended to further investigate the roles of HERs in phenotypic variability and evolutionary processes within livestock populations. To achieve more reliable simulation results, it is crucial to understand how HERs are inherited across generations and the mechanisms that influence their presence and concentration in the animal genome. Additionally, it is important to consider the emergence and maintenance of variation, which depends on selection schemes affecting genetic variants, the genetic architecture of complex traits (including the number of genes controlling traits, their effects, and genetic linkage), and the coordination of allele expression [18]. This comprehensive understanding will provide the necessary foundation for conducting simulations that accurately reflect the underlying biological processes of HERs in the cattle genome.

## Conclusions

The identification of HERs depends on the parameters used to assess the heterozygosity of the regions. The minimum length of 10 Kb resulted in the highest number of HERs detected, confirming that HERs are small regions scattered throughout the genome. The minimum number of markers that define a HER and the maximum number of homozygous allowed inside a HER did not show substantial impact based on the data sources evaluated, being more linked to the population structure and quality of genotyping. Forty-five HER islands were identified in all scenarios of parameter combinations, with high Tajima’s D values indicating that such regions are likely under balancing selection. In general, those regions have a small linkage disequilibrium and are related to traits such as fertility, production, and immune response. As for the breed comparisons, the majority of the identified HERs were in common among the four taurine breeds (> 94%), regardless of the selection forces each breed went through over the generations.

## Data Availability

All the necessary information to support the results of this study are included within the article and supplementary material. Data that support the findings of this study are available from the Centre of Genetic Improvement of Livestock (CGIL, University of Guelph, Guelph, ON, Canada) based upon reasonable request and permission from the 1000 Bull Genomes Project.

## Appendix

Supplementary Material 1. Supplementary Material 2. Supplementary Material 3. Supplementary Material 4. Supplementary Material 5. Supplementary Material 6. Supplementary Material 7. Supplementary Material 8. Supplementary Material 9.
