# Supplementary figures and images for: Detection and evaluation of parameters influencing the identification of heterozygous-enriched regions in Holstein cattle based on SNP chip or whole-genome sequence data
Source: BMC Genomics. 2024 Jul 26;25:726. doi: 10.1186/s12864-024-10642-2 (PMC11282608; doi:10.1186/s12864-024-10642-2)

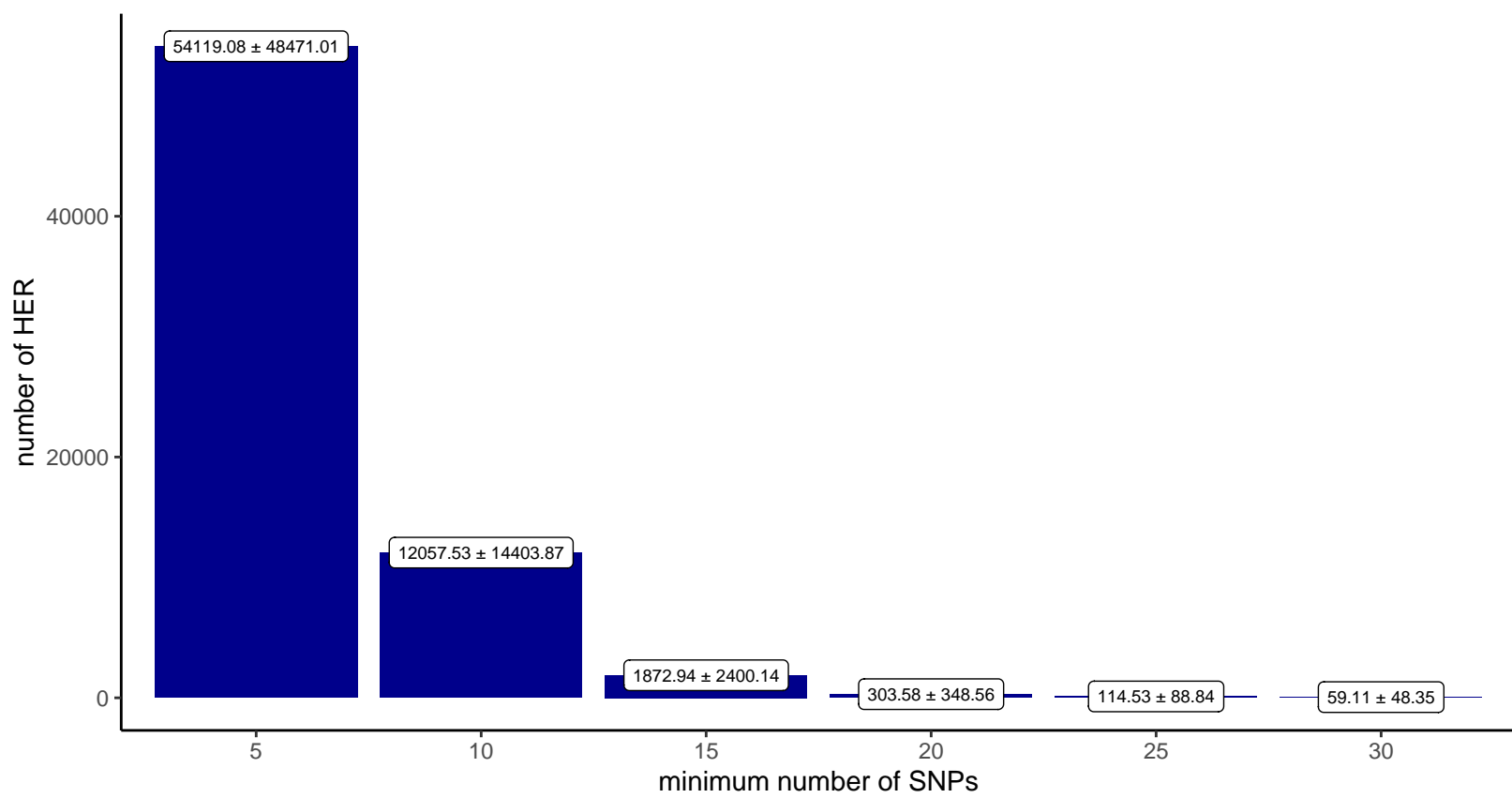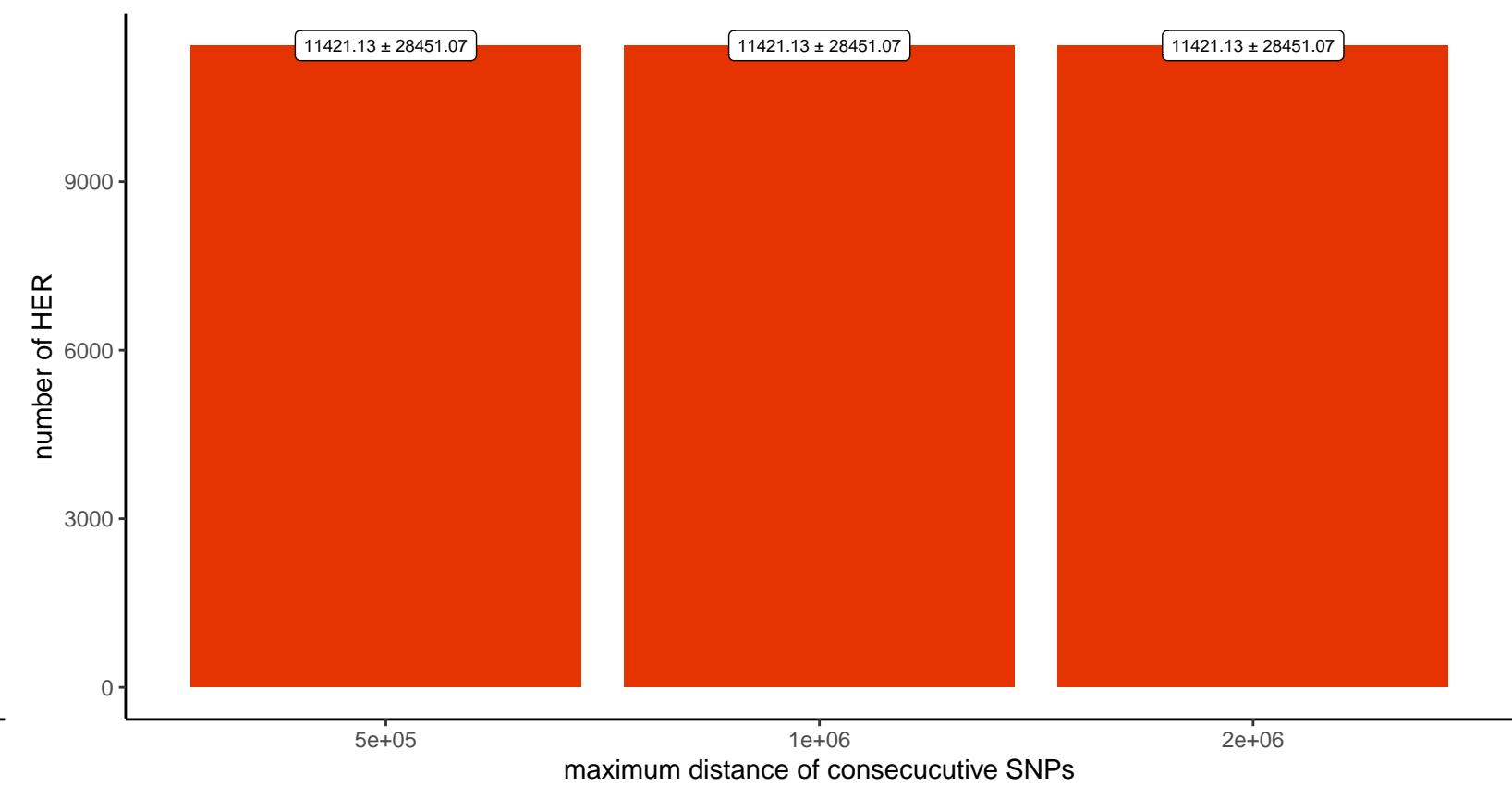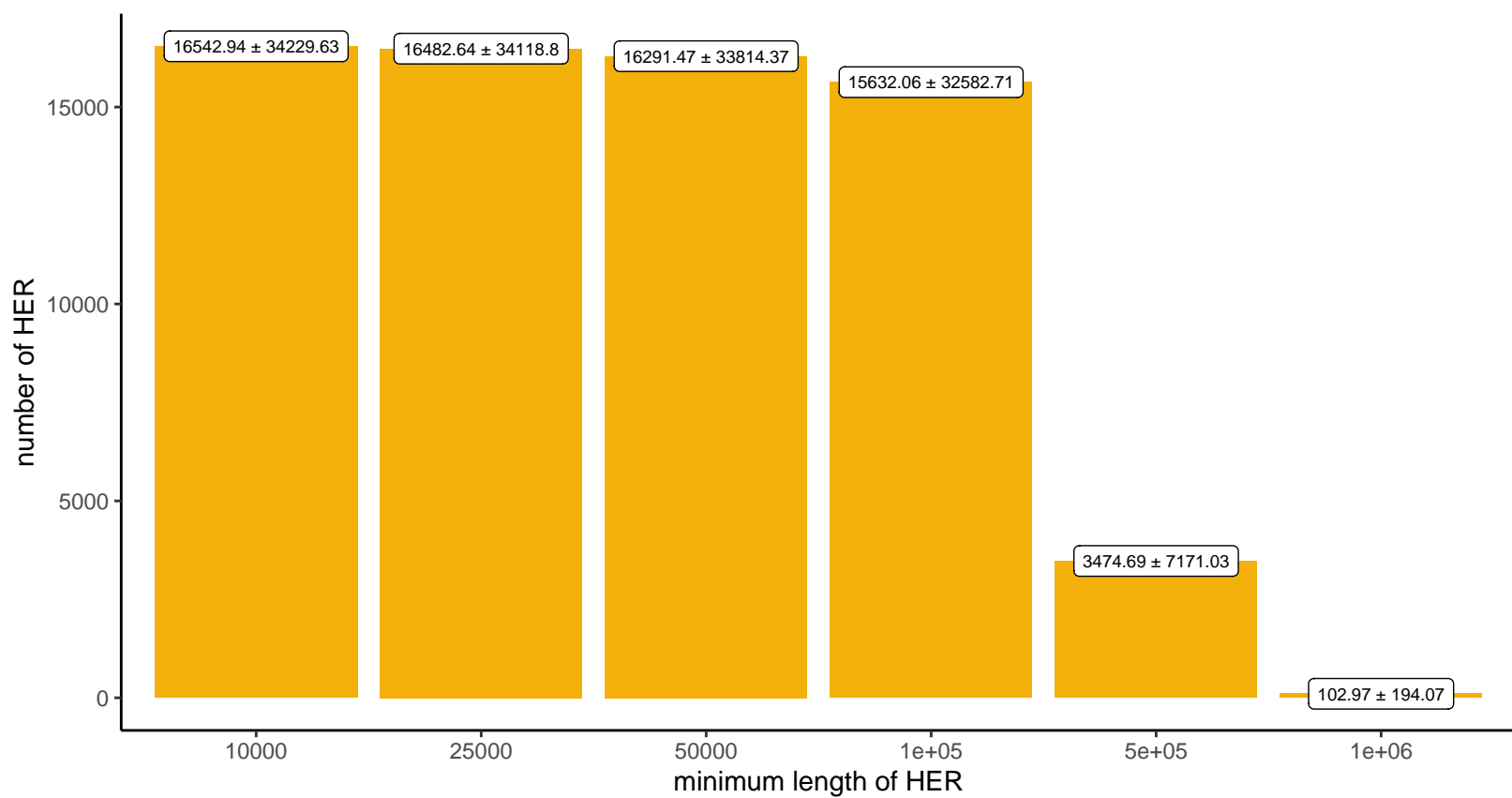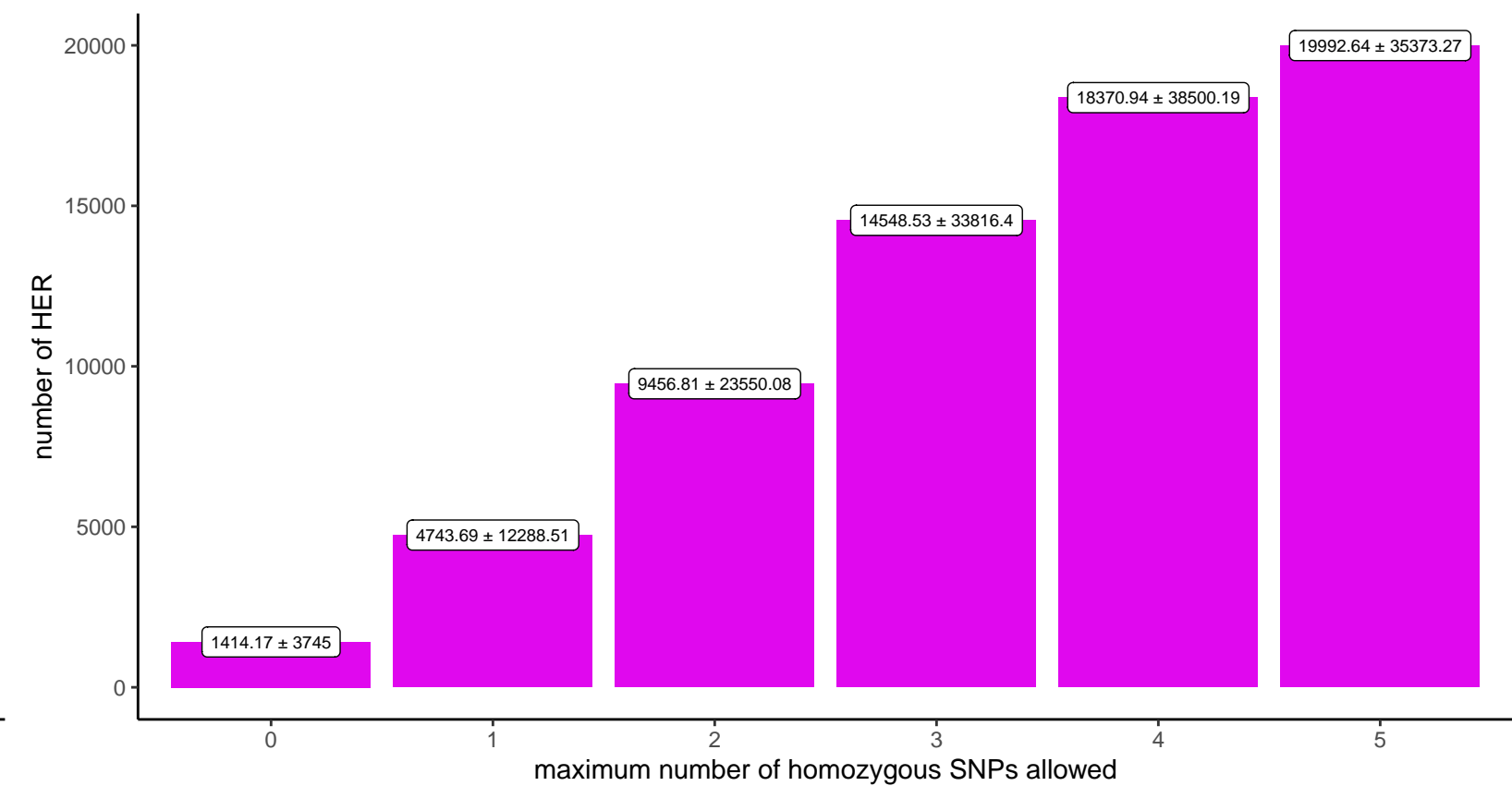

Supplement: Supplementary file 1 — Supplementary Material 1. [file 12864_2024_10642_MOESM1_ESM.pdf]

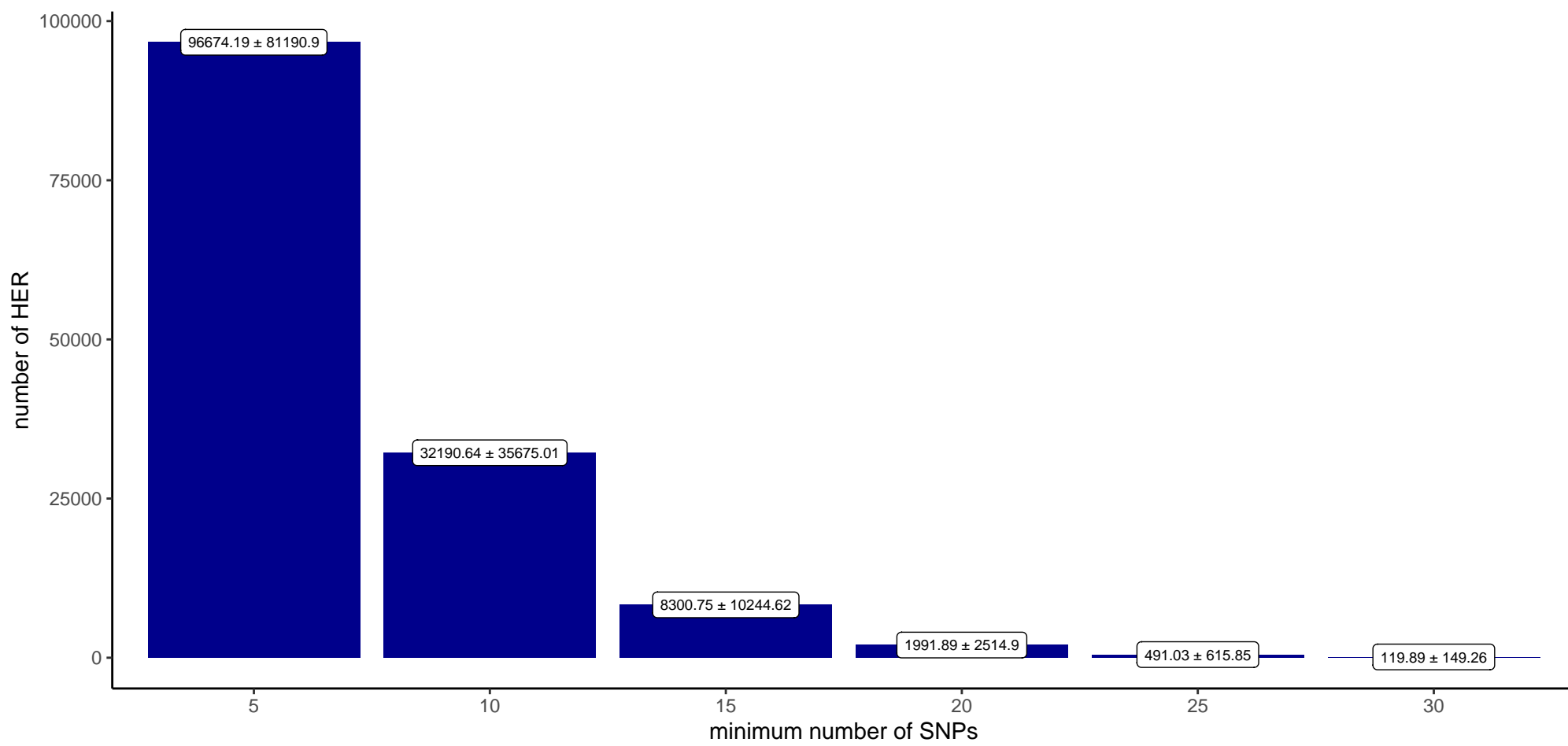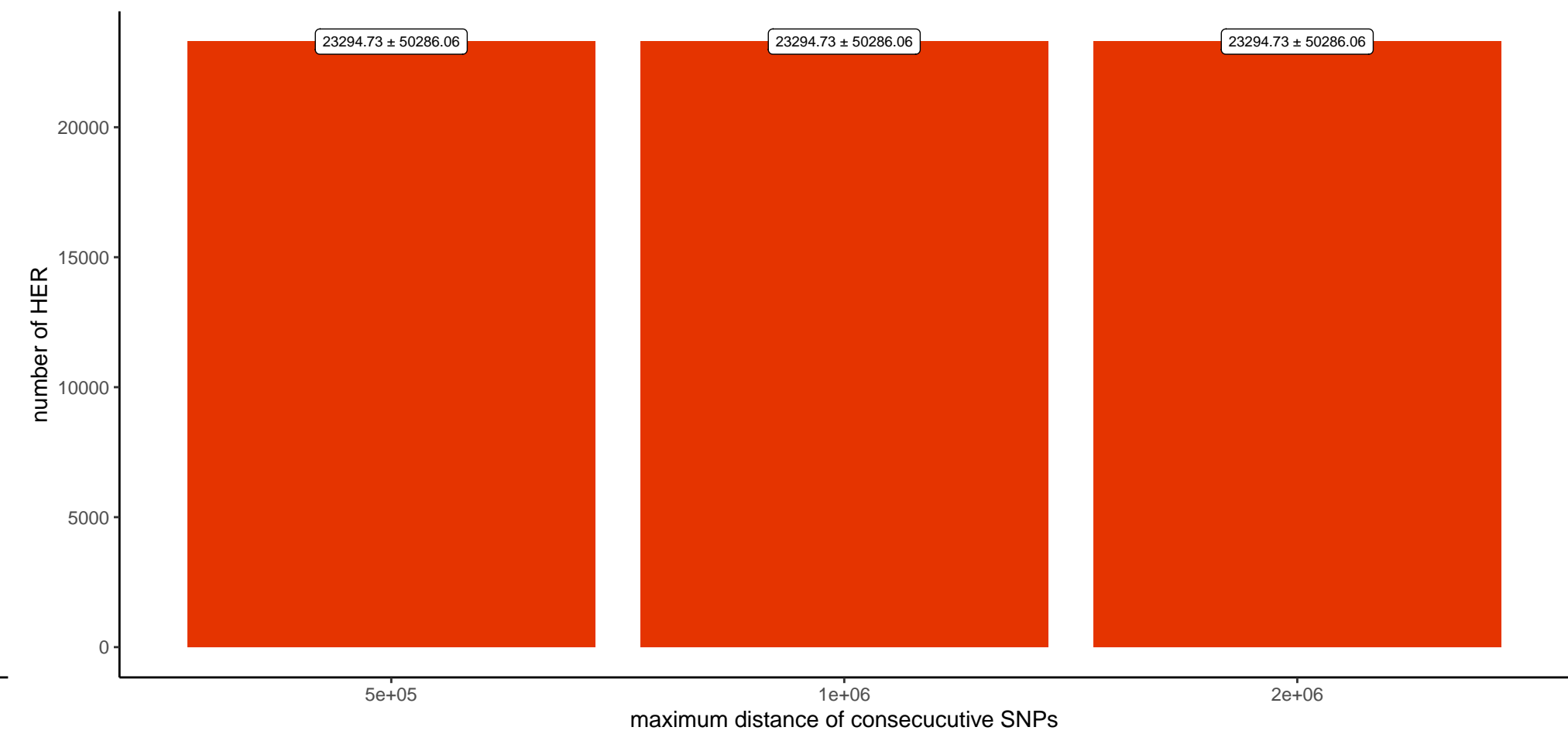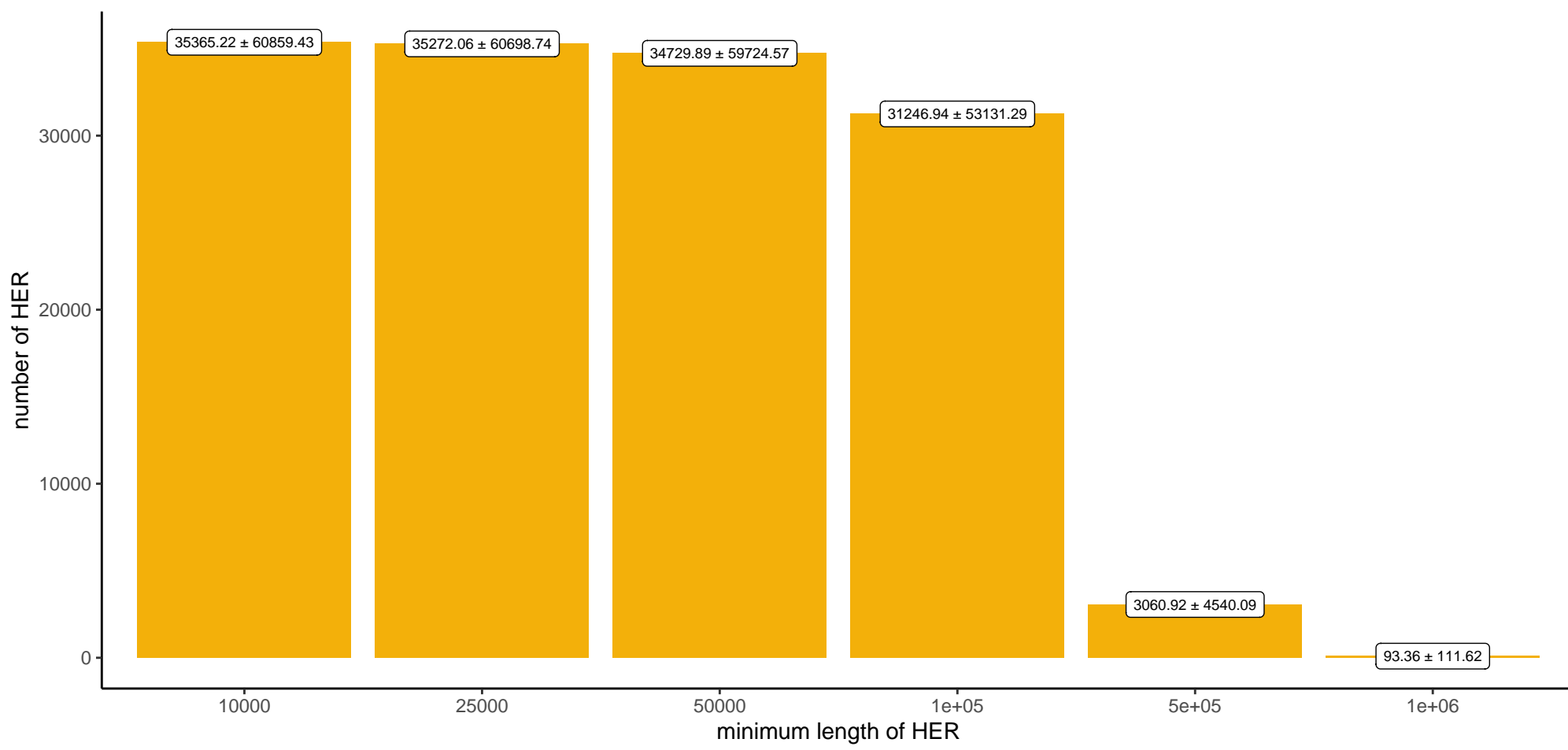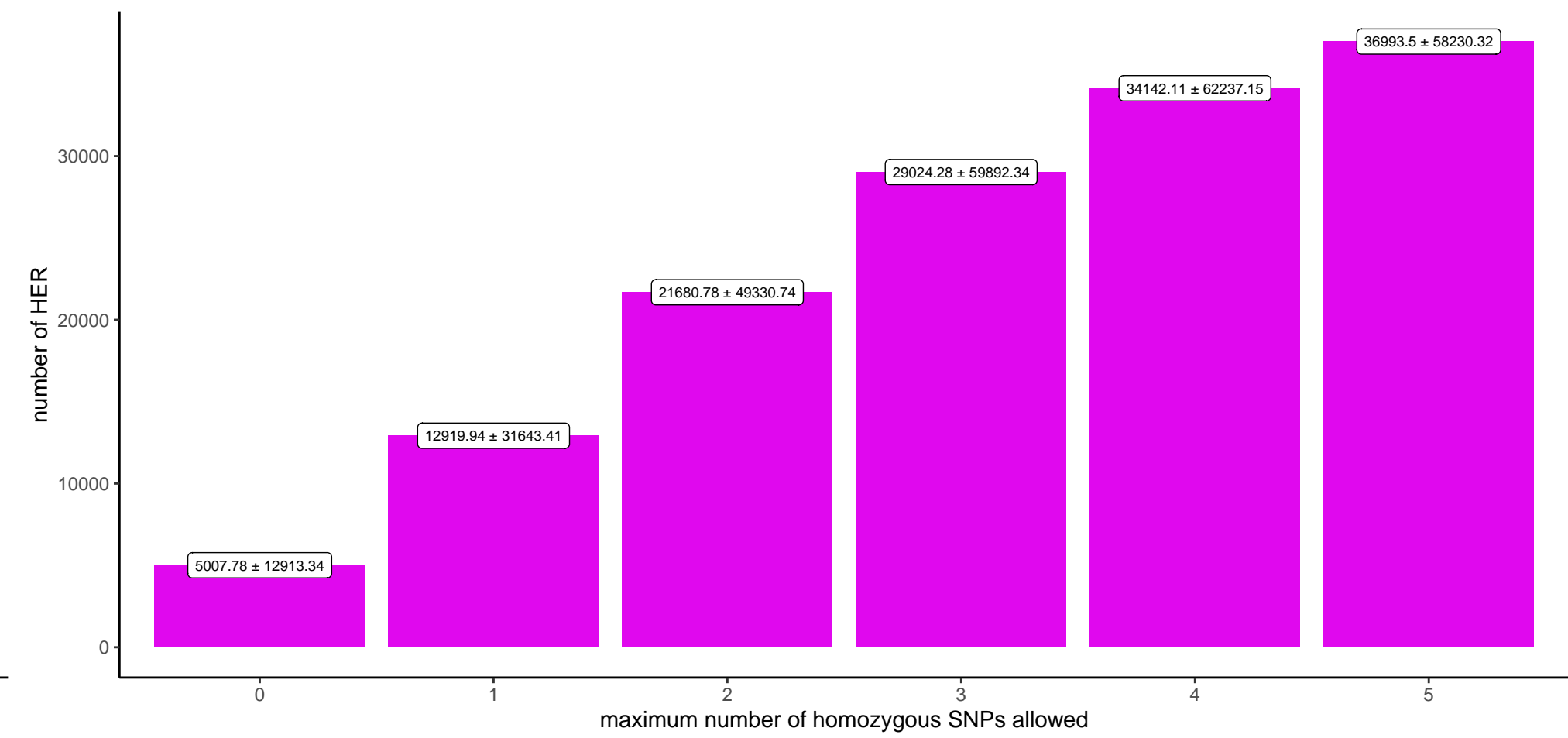

Supplement: Supplementary file 2 — Supplementary Material 2. [file 12864_2024_10642_MOESM2_ESM.pdf]

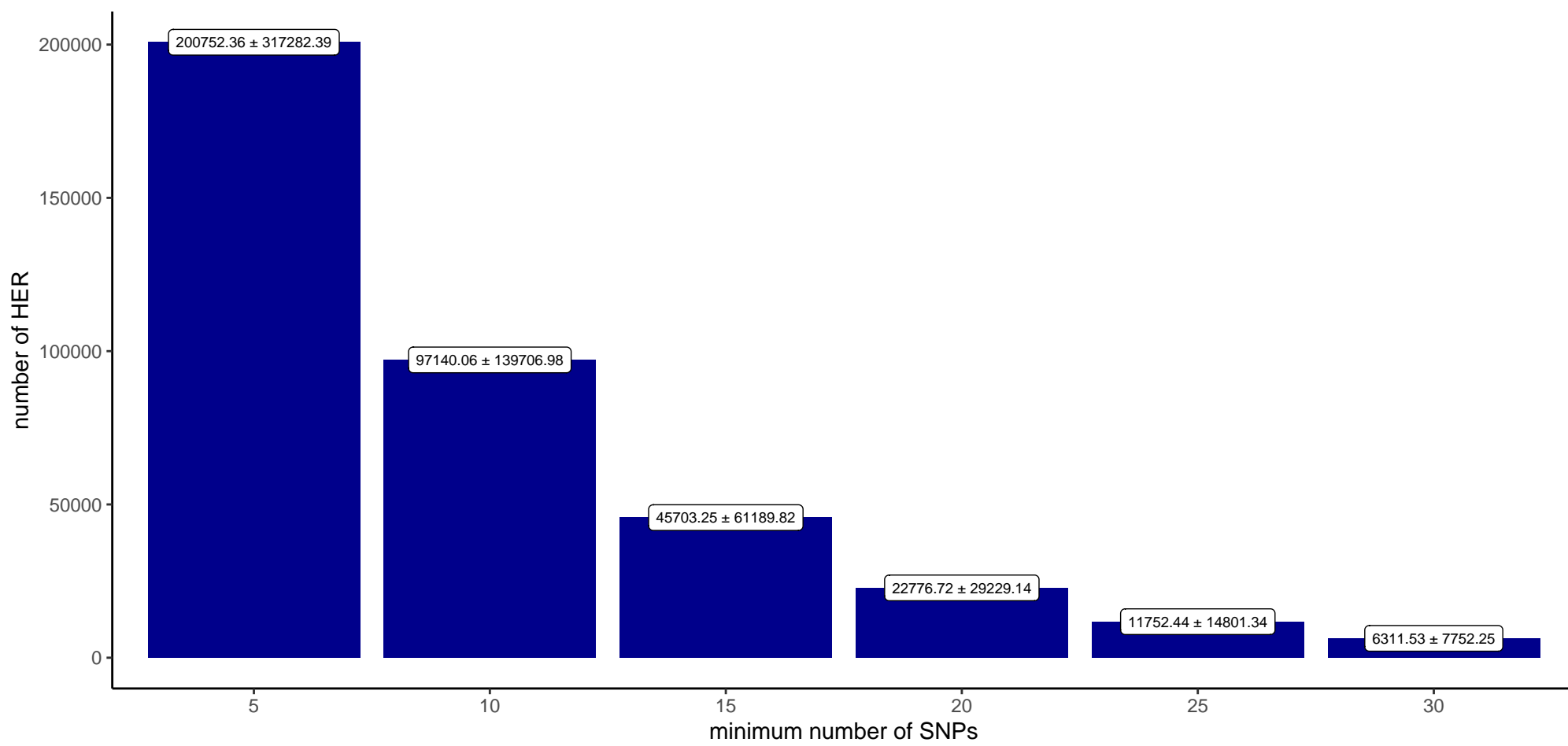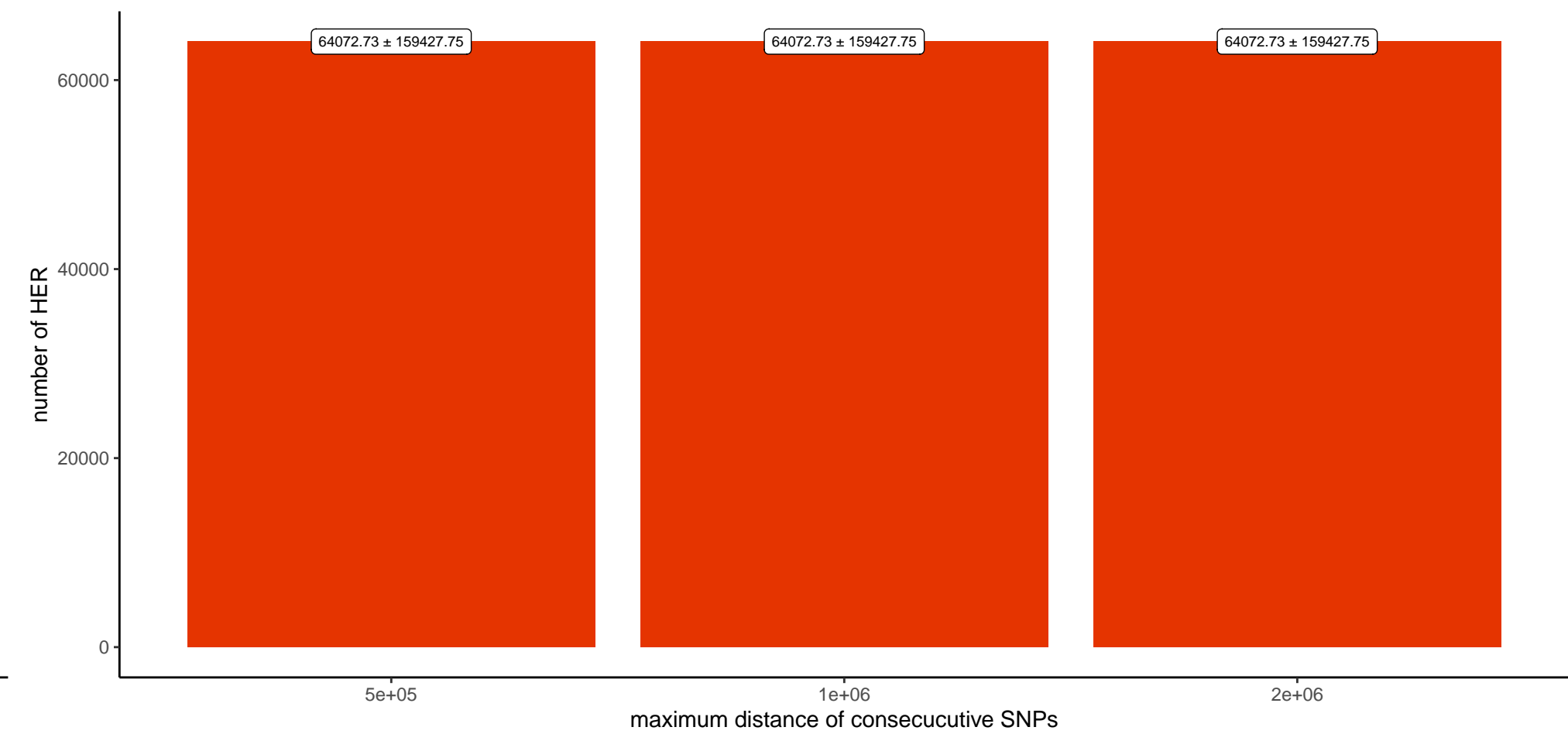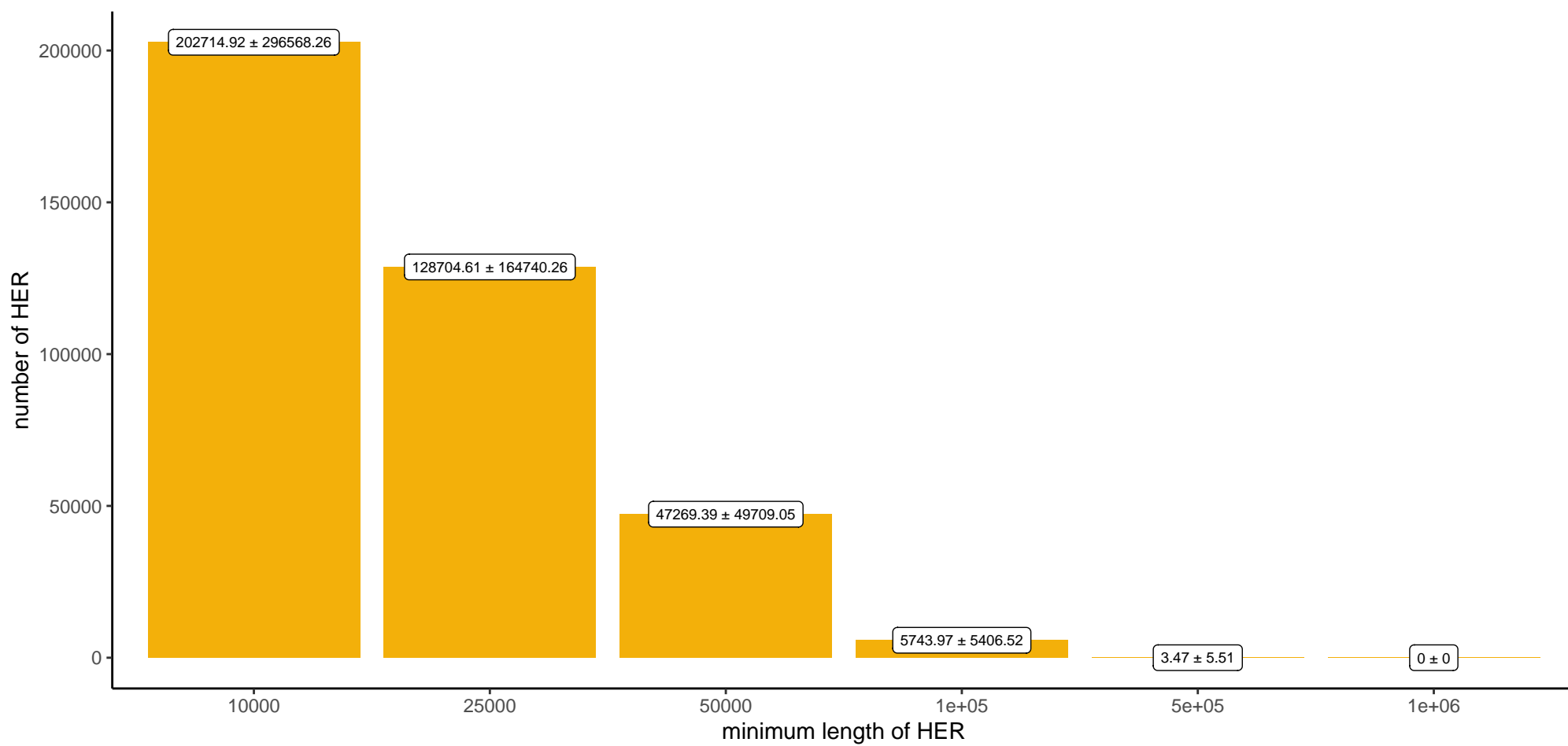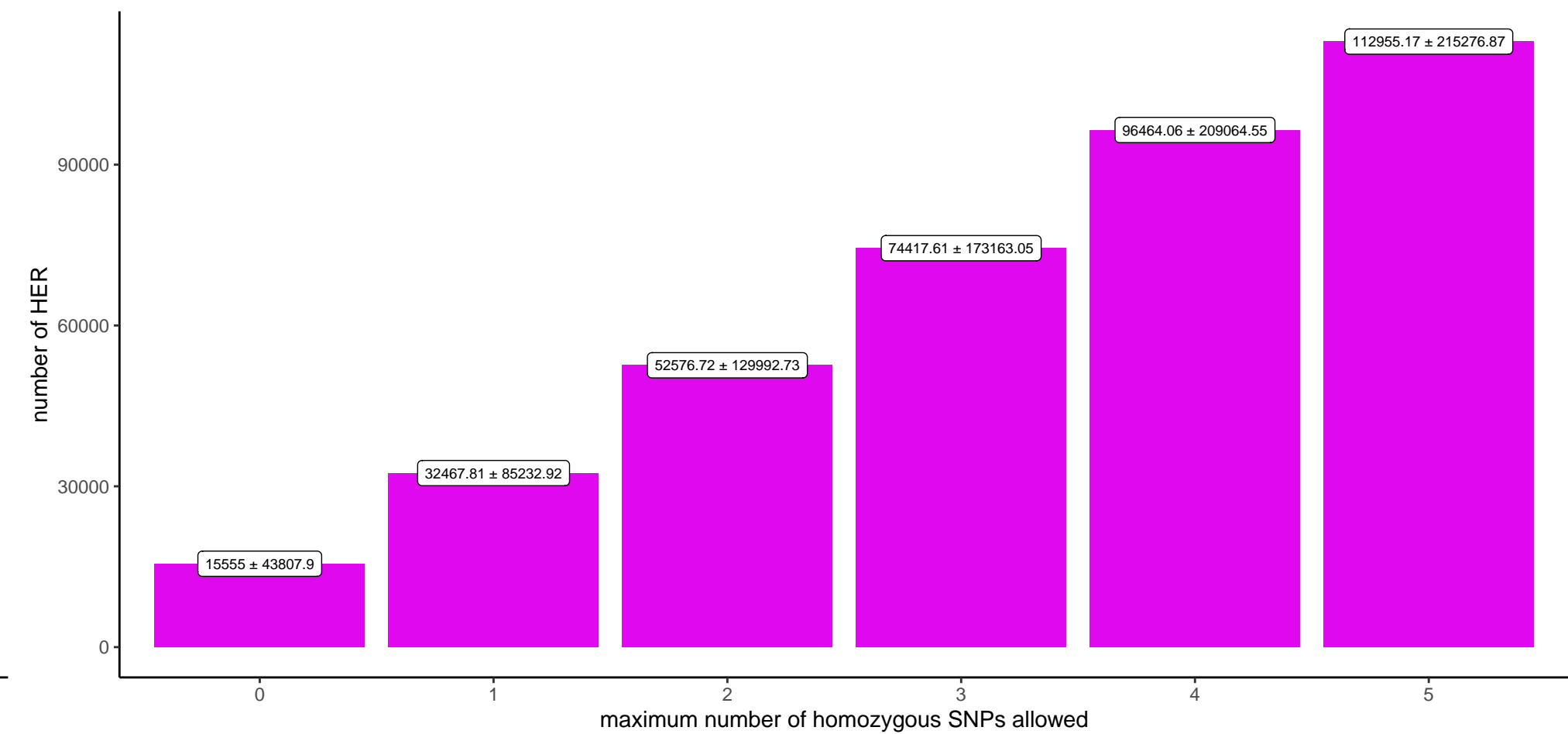

Supplement: Supplementary file 3 — Supplementary Material 3. [file 12864_2024_10642_MOESM3_ESM.pdf]

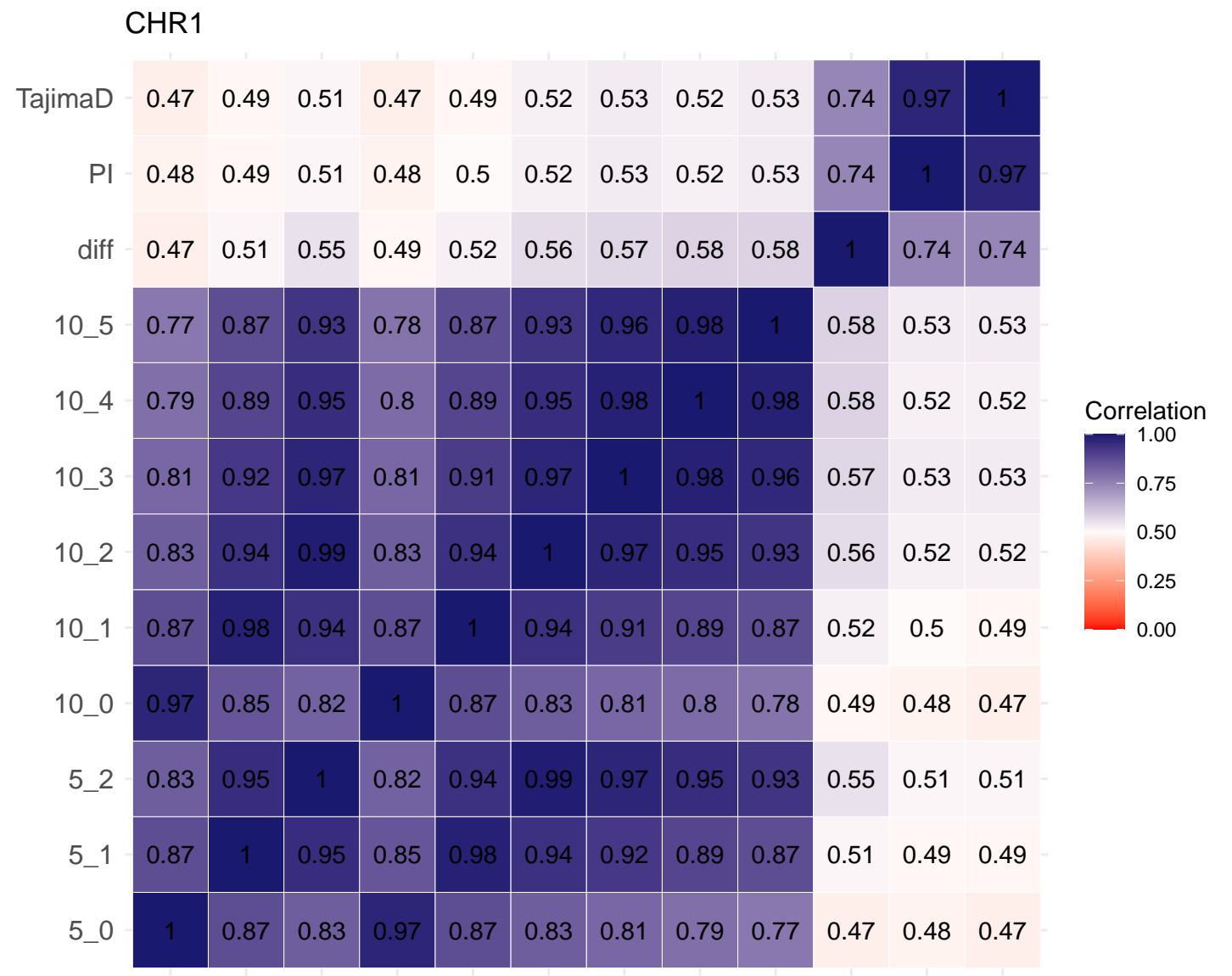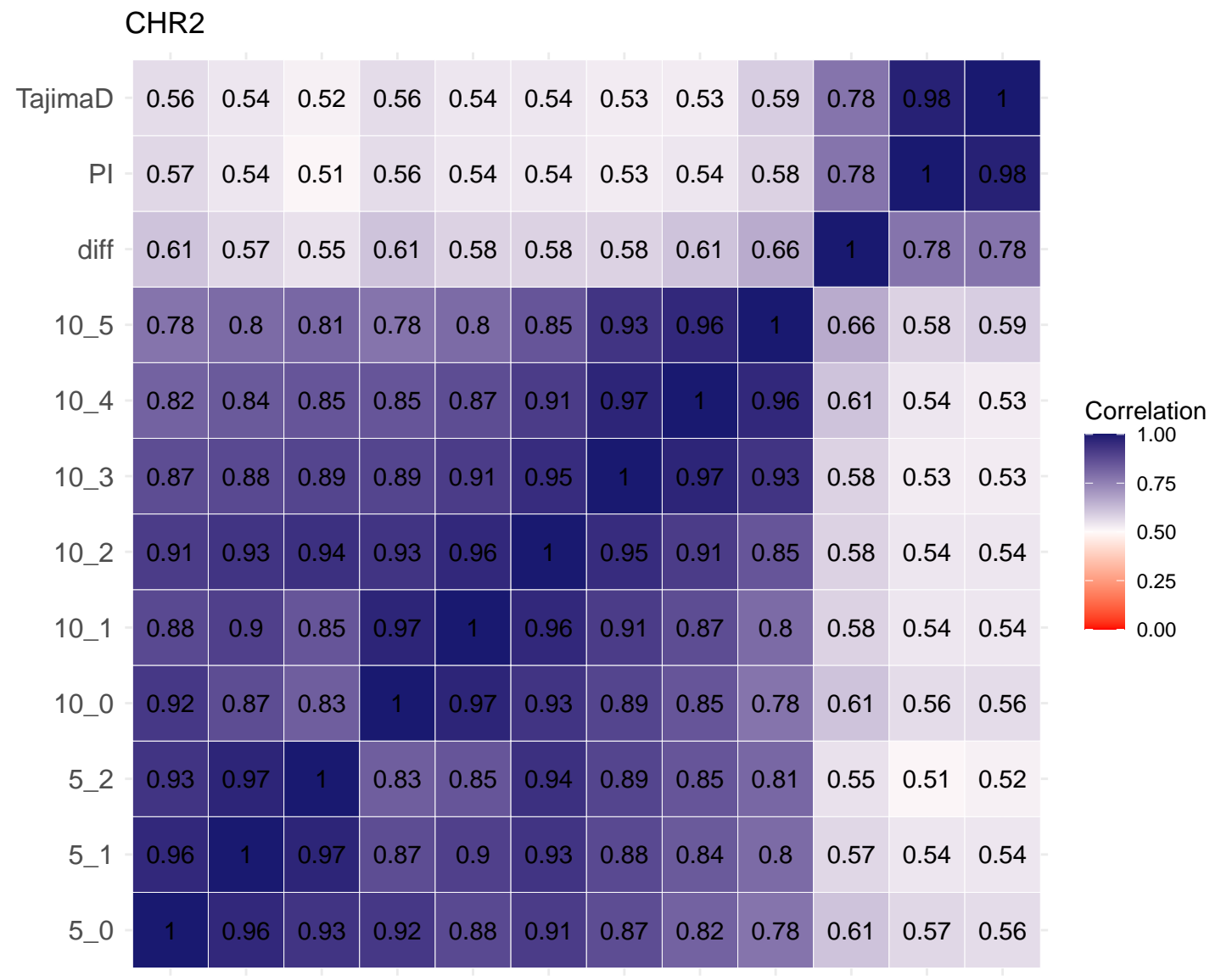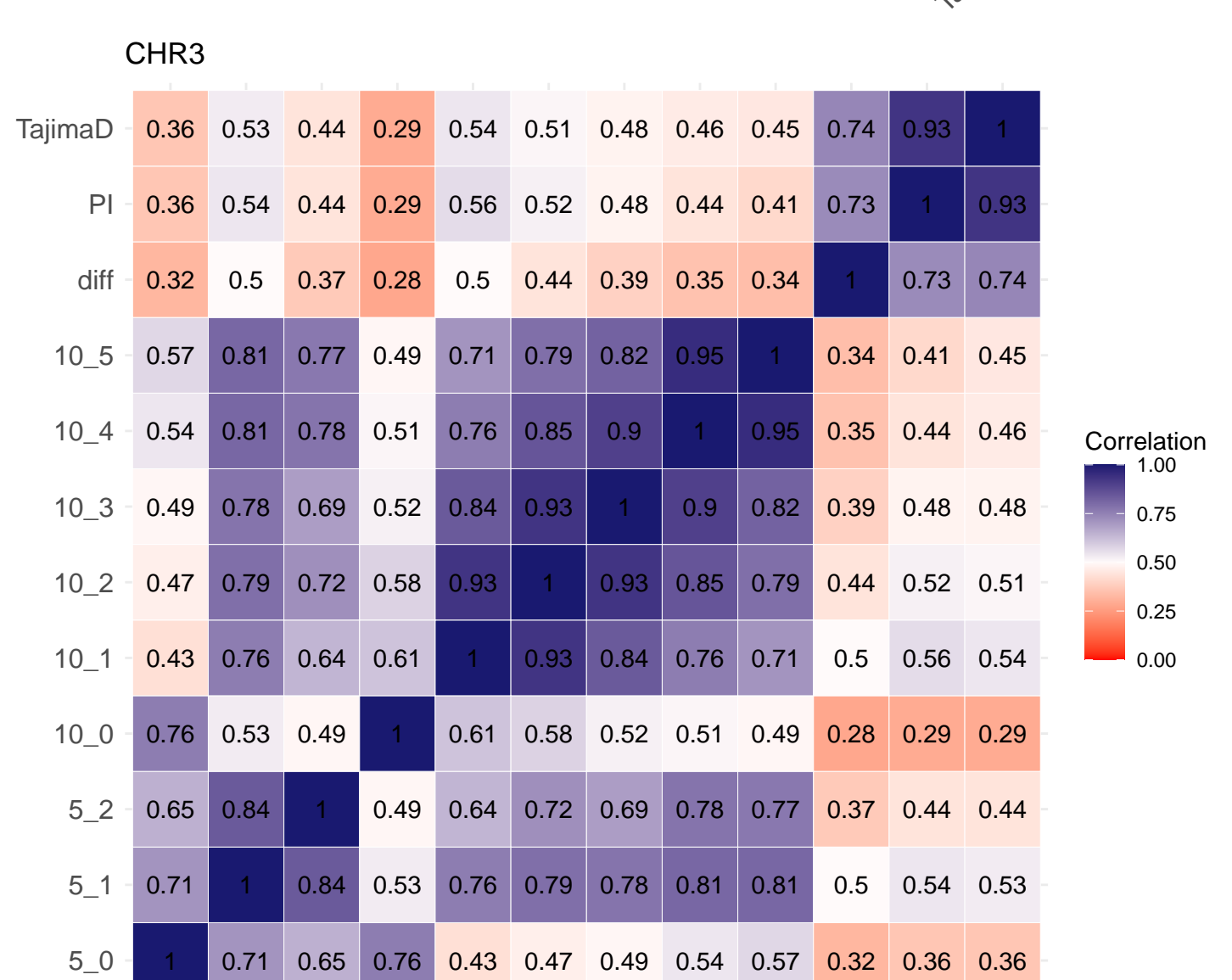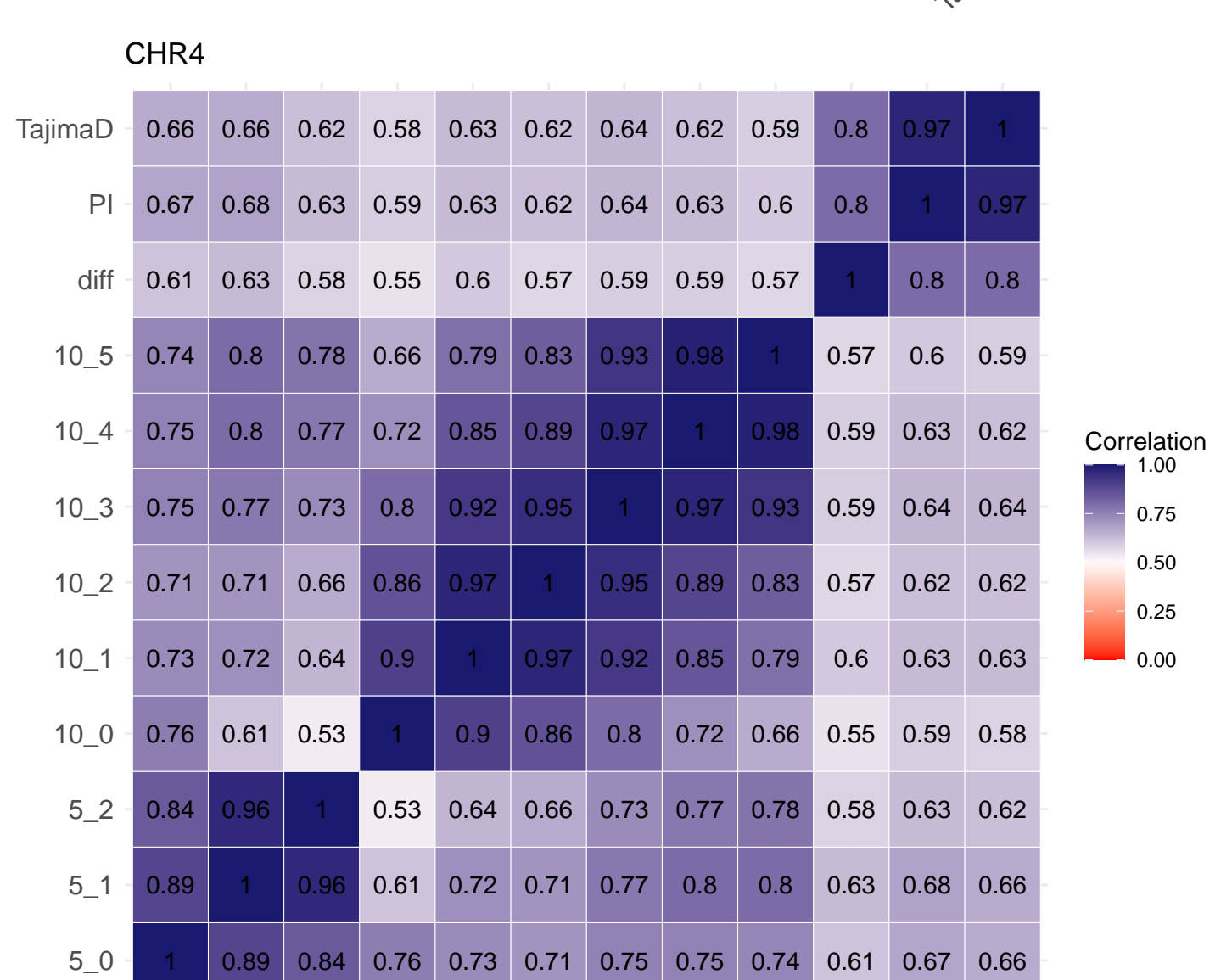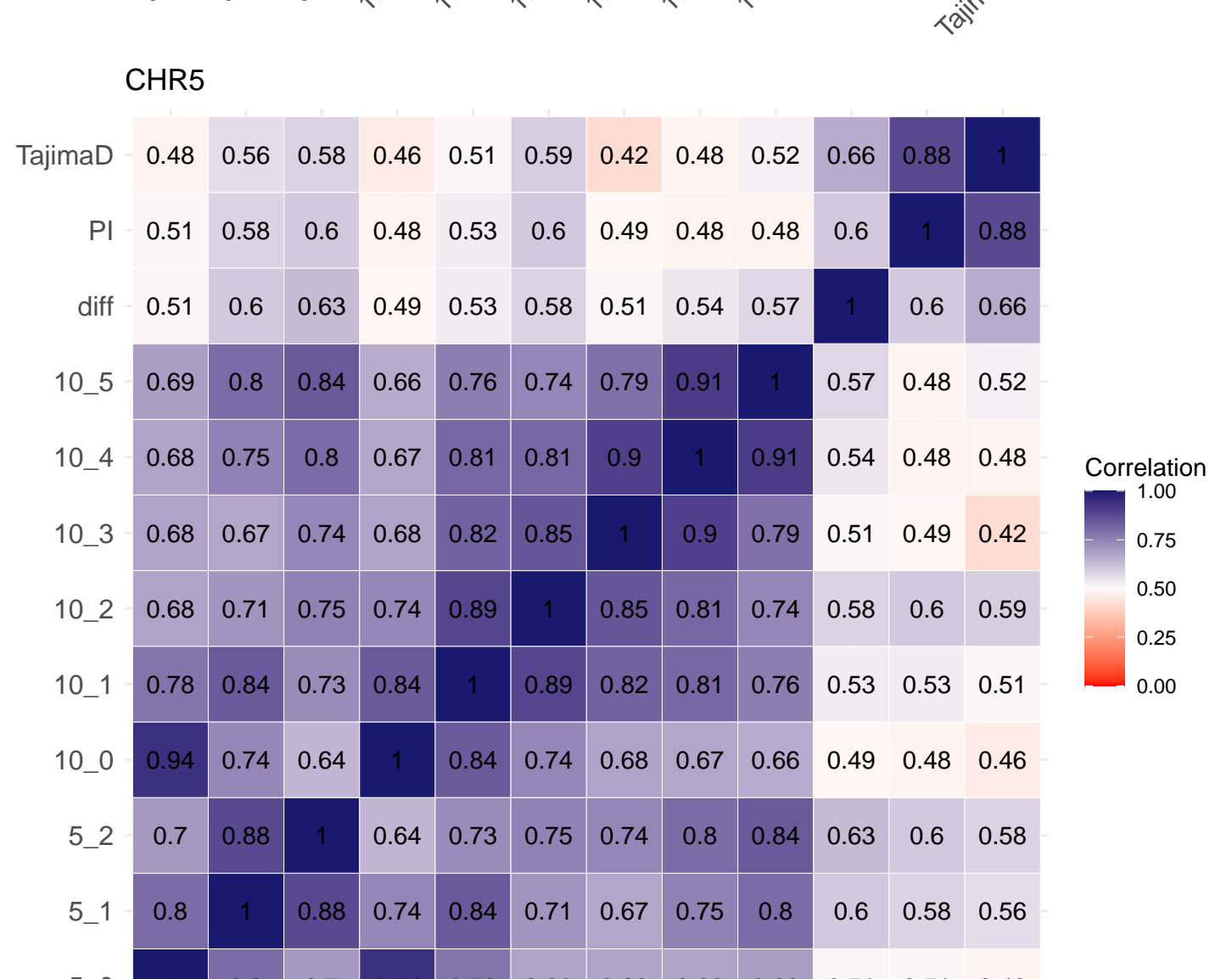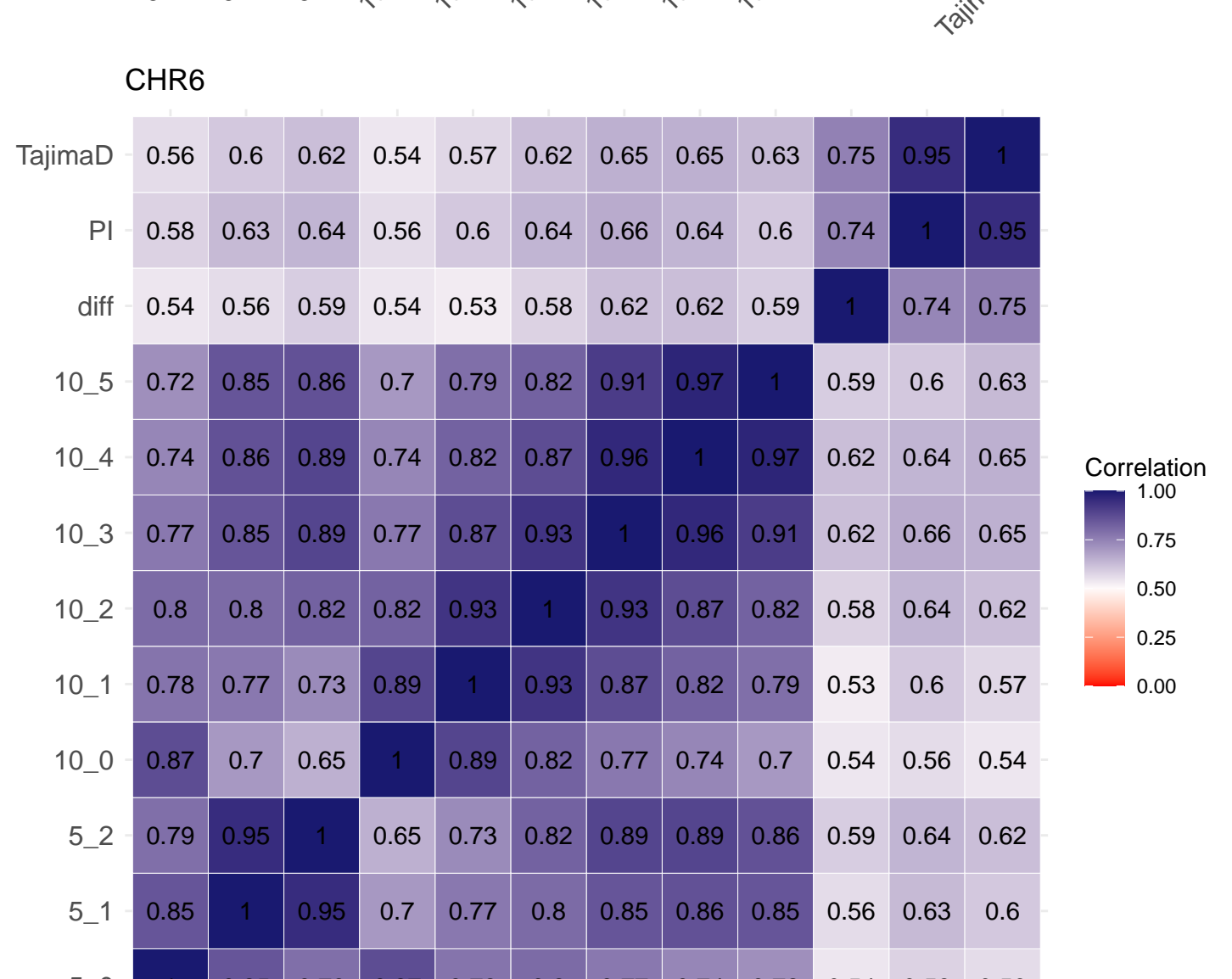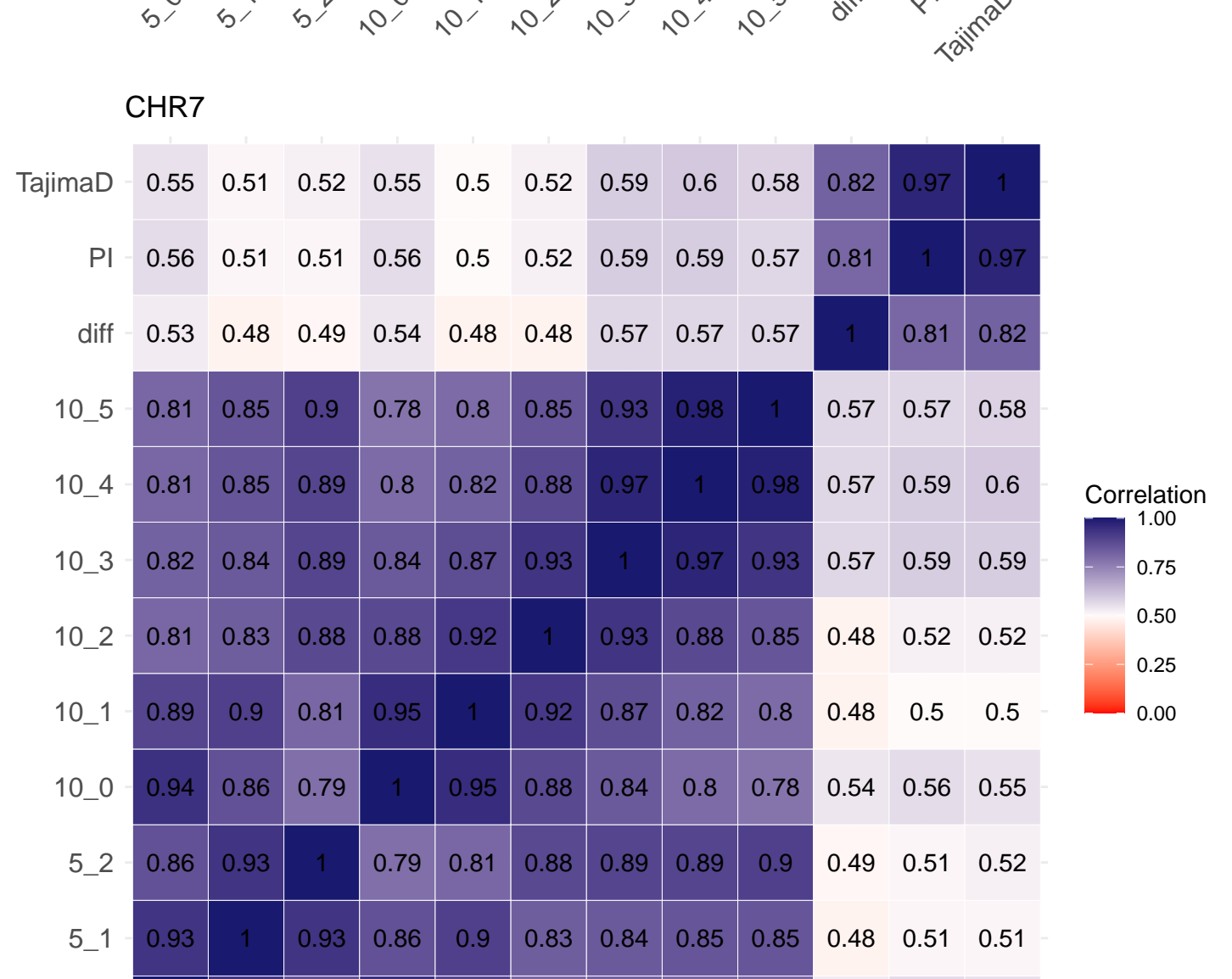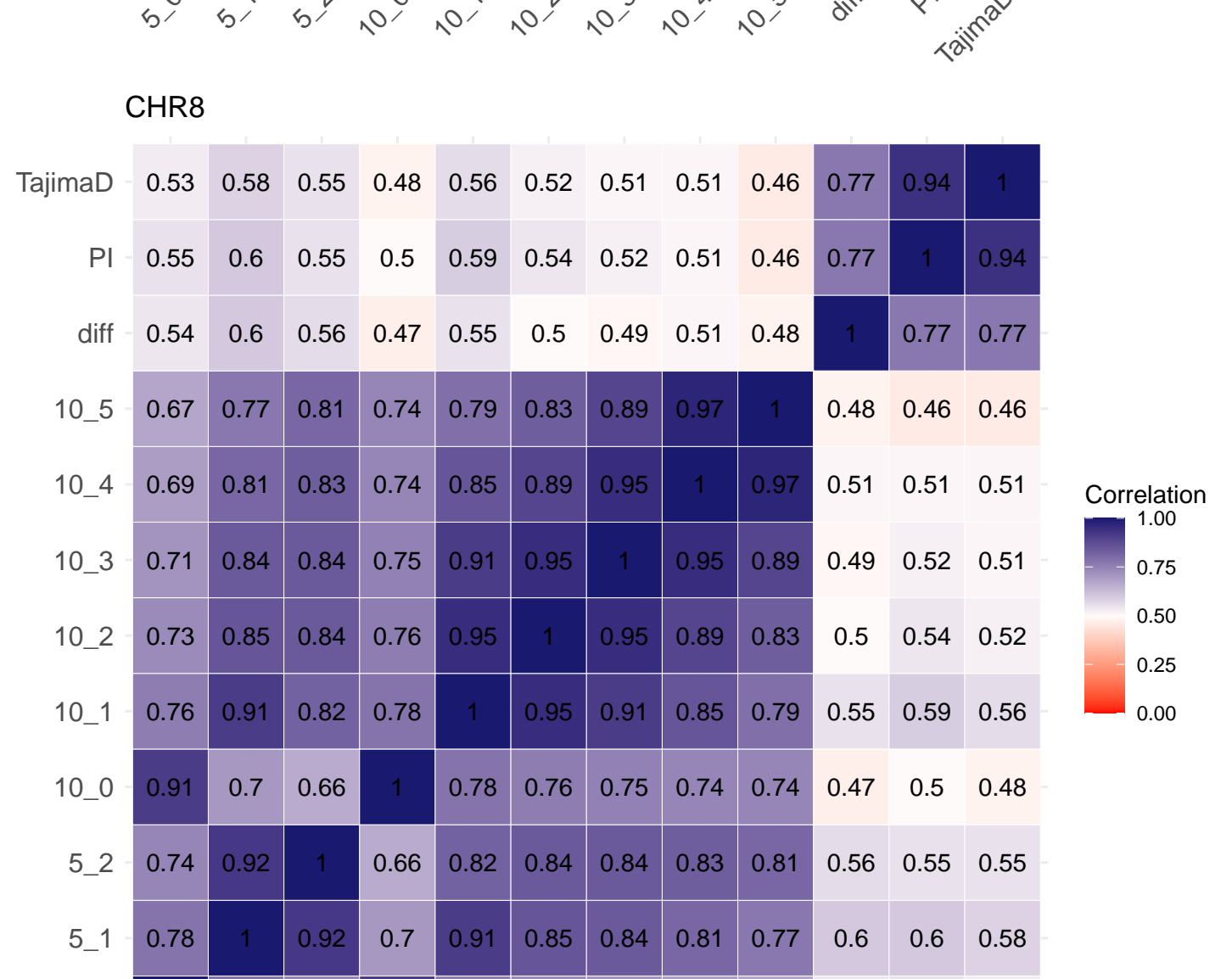

Supplement: Supplementary file 7 — Supplementary Material 7. [file 12864_2024_10642_MOESM7_ESM.pdf]
