# Supplementary material for: Detection and evaluation of parameters influencing the identification of heterozygous-enriched regions in Holstein cattle based on SNP chip or whole-genome sequence data
Source: BMC Genomics. 2024 Jul 26;25:726. doi: 10.1186/s12864-024-10642-2 (PMC11282608; doi:10.1186/s12864-024-10642-2)
Supplement: Supplementary file 8 — Supplementary Material 8. [file 12864_2024_10642_MOESM8_ESM.pdf]

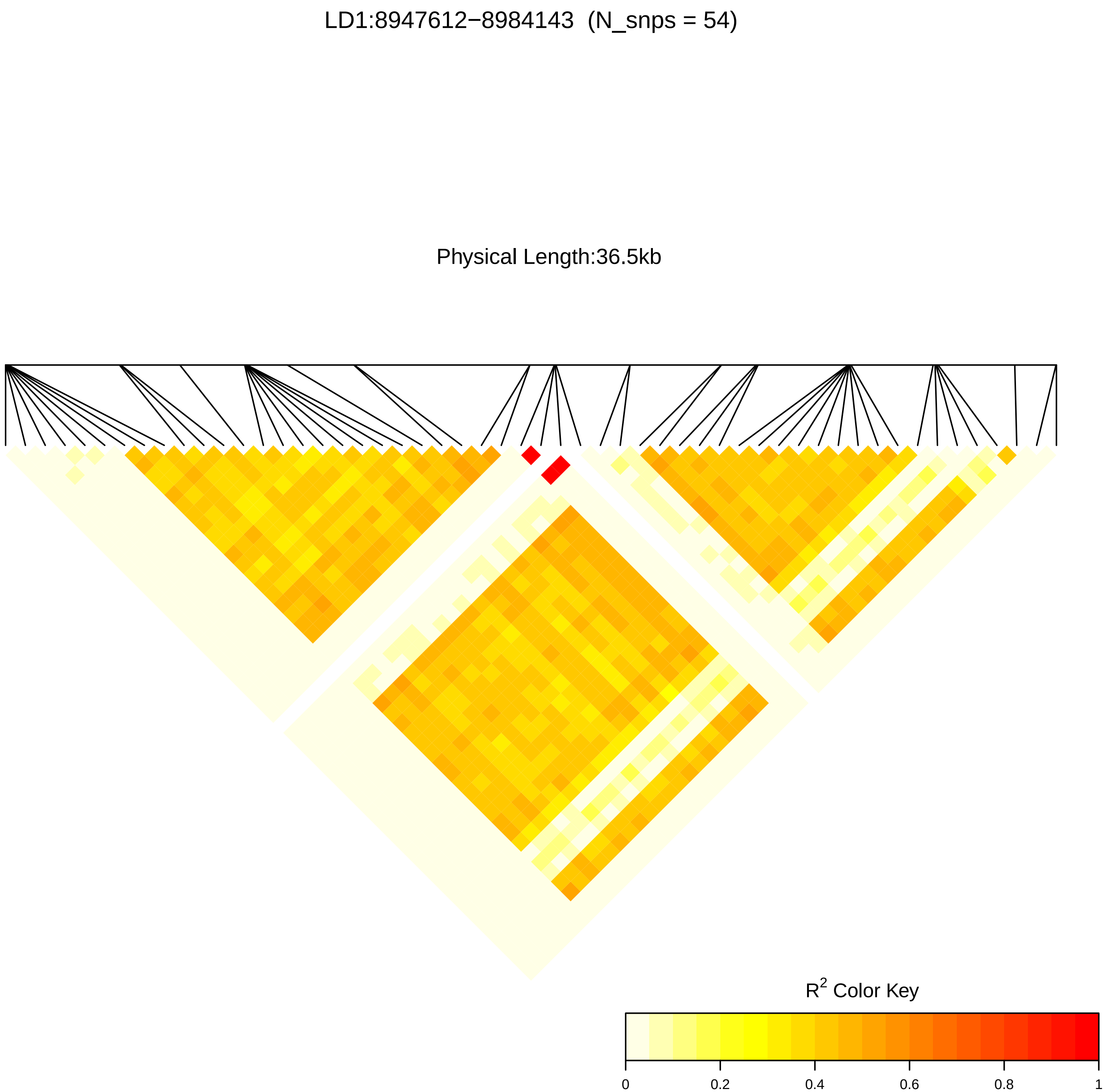
LD1:8947612−8984143 (N_snps = 54)
Physical Length:36.5kb
R^2 Color Key
0
0.2
0.4
0.6
0.8
1

LD1:77541594−77574520 (N_snps = 114)

Physical Length:32.9kb

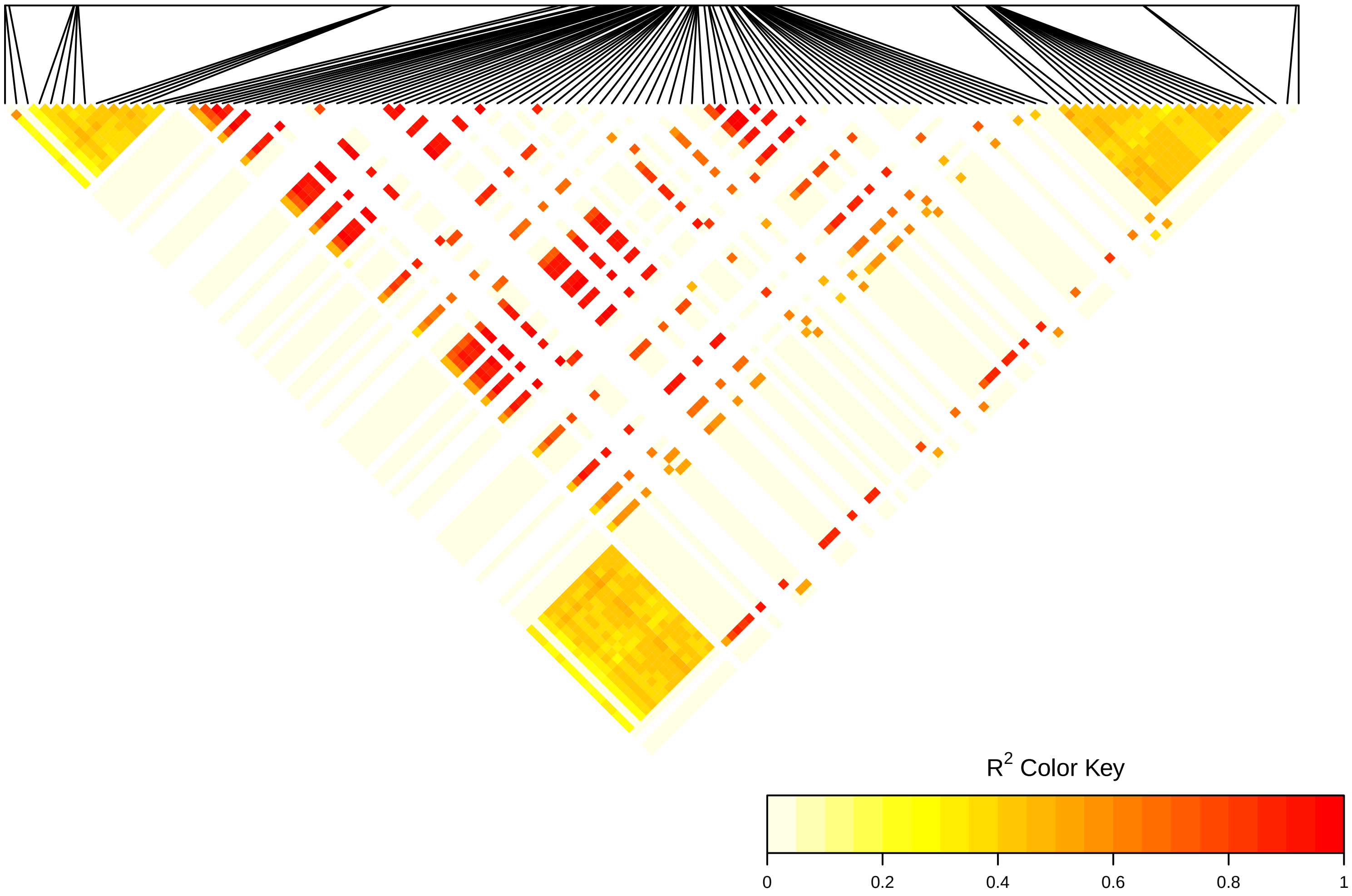

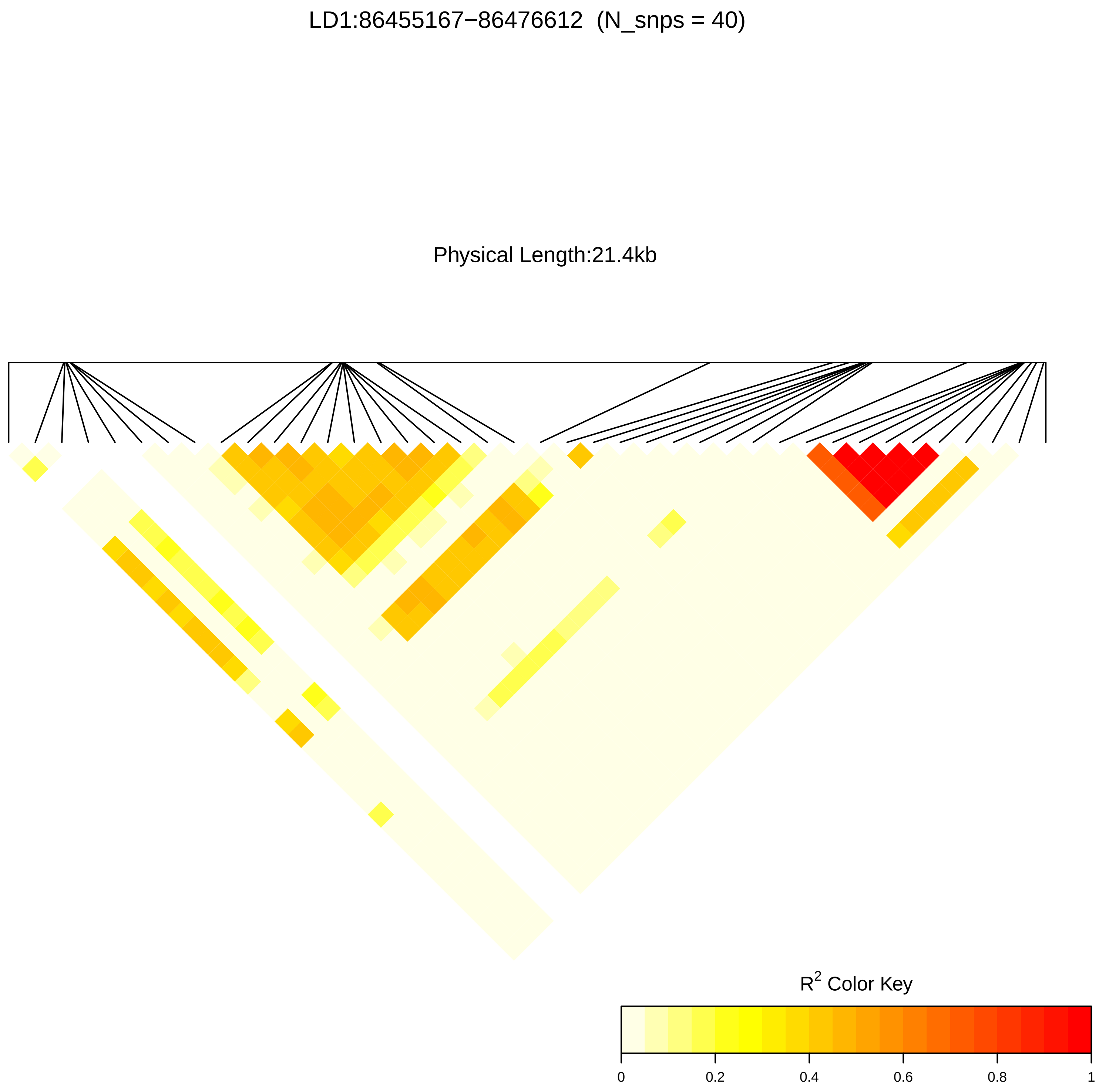
LD1:86455167−86476612 (N_snps = 40)
Physical Length:21.4kb
R2 Color Key
0
0.2
0.4
0.6
0.8
1

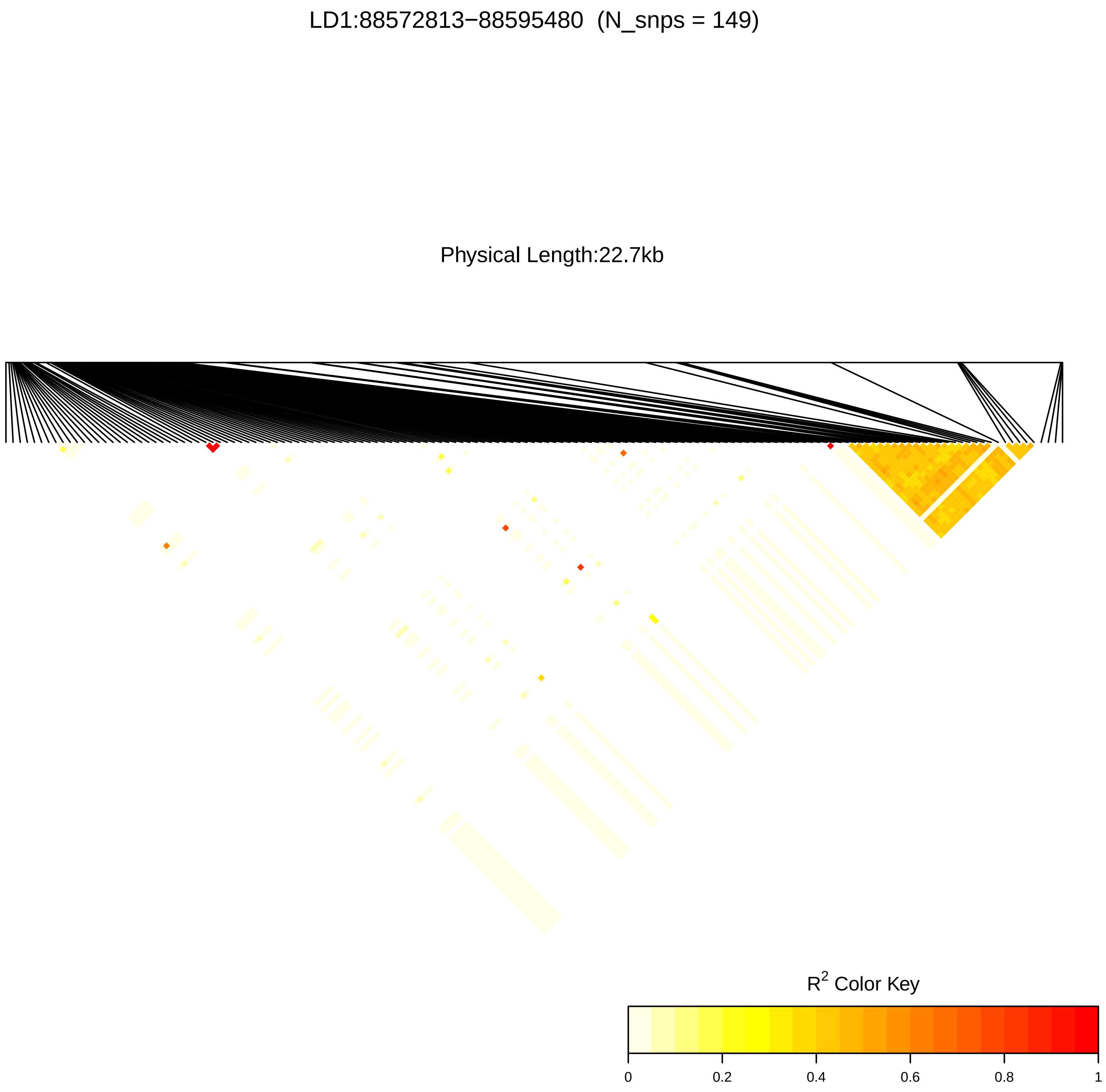
LD1:88572813−88595480 (N_snps = 149)
Physical Length:22.7kb
R2 Color Key
0
0.2
0.4
0.6
0.8
1

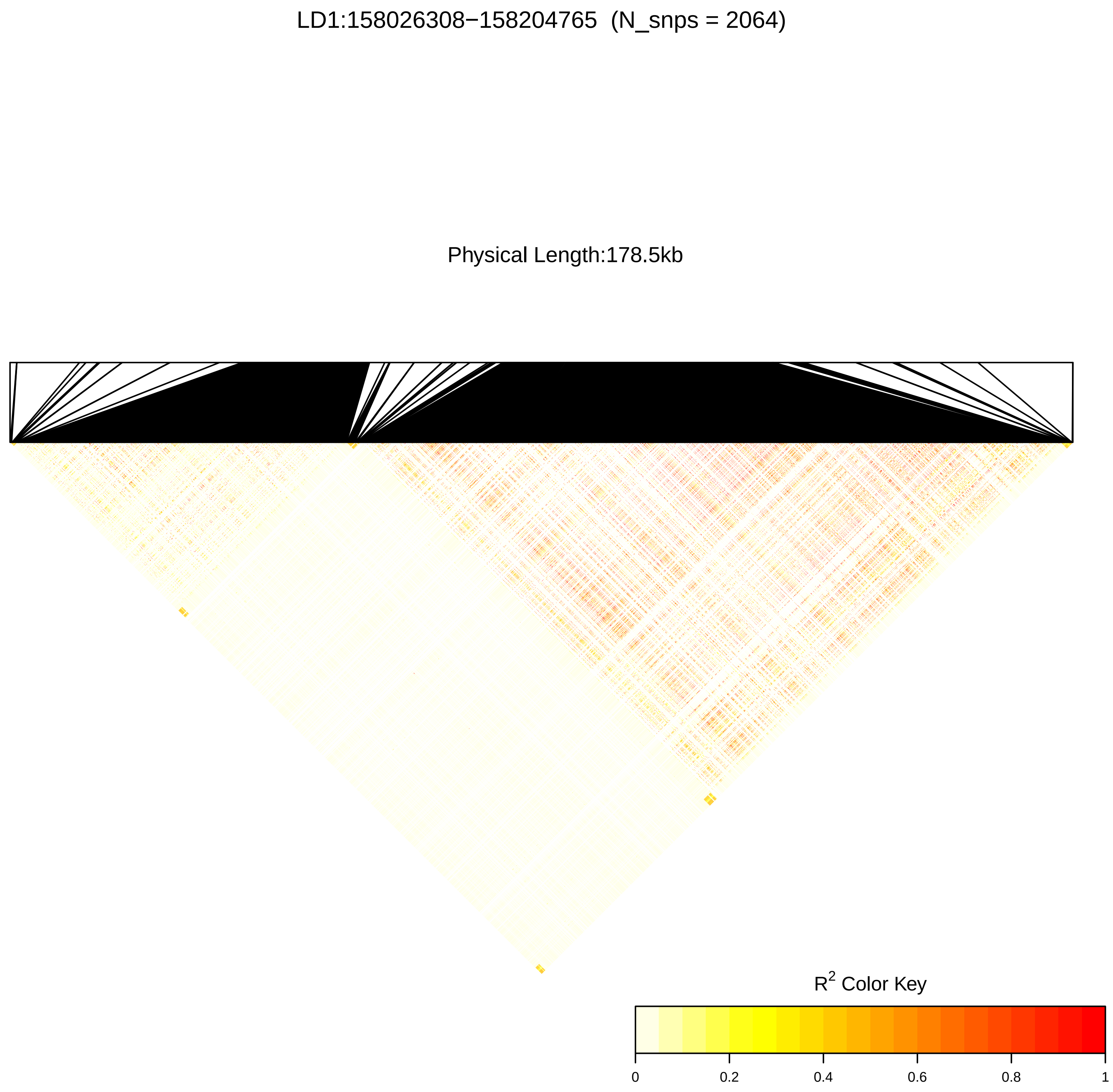
LD1:158026308−158204765 (N_snps = 2064)
Physical Length:178.5kb
R2 Color Key
0
0.2
0.4
0.6
0.8
1

LD2:11729−23106 (N_snps = 13)

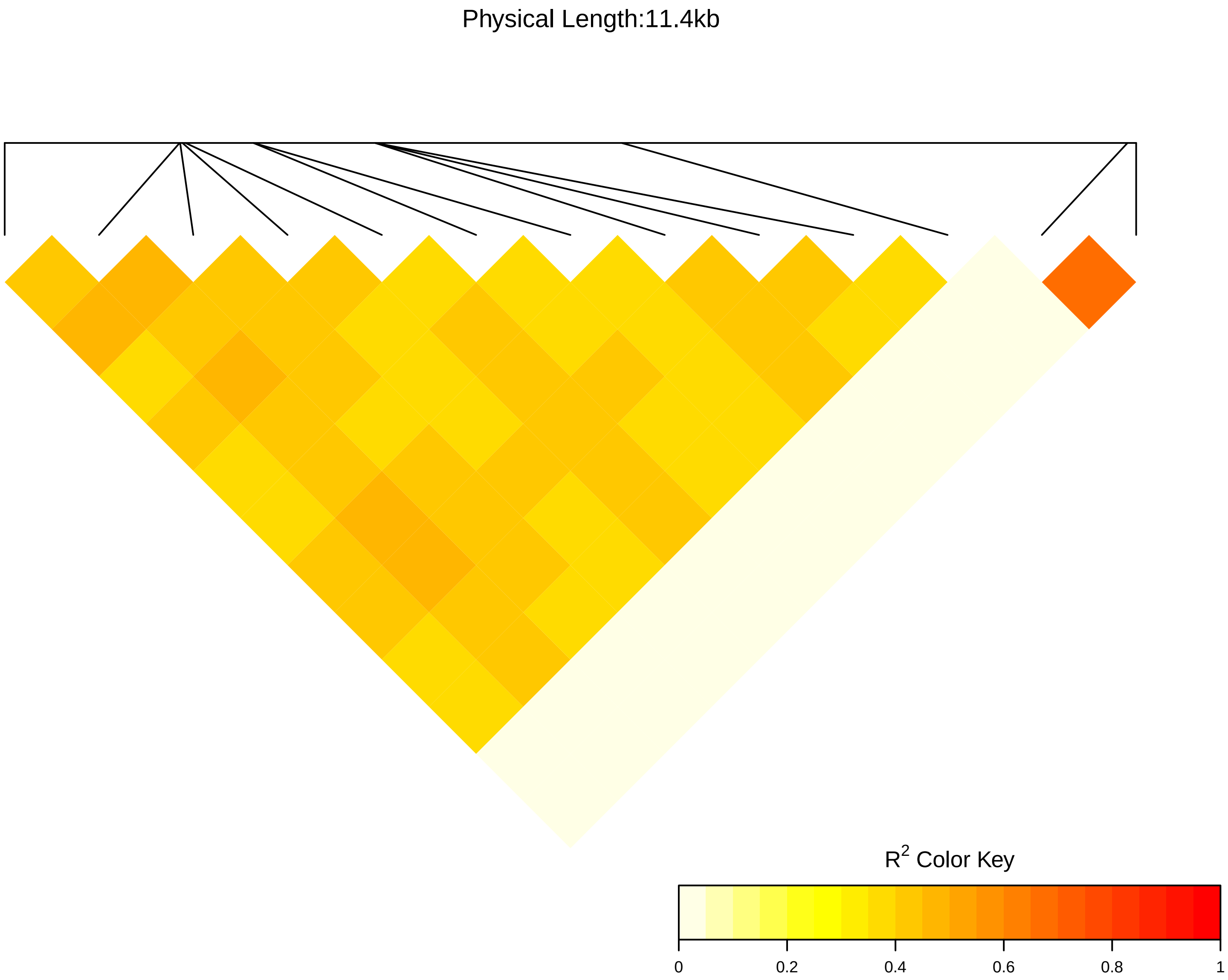

LD2:121358897−121376671 (N_snps = 23)

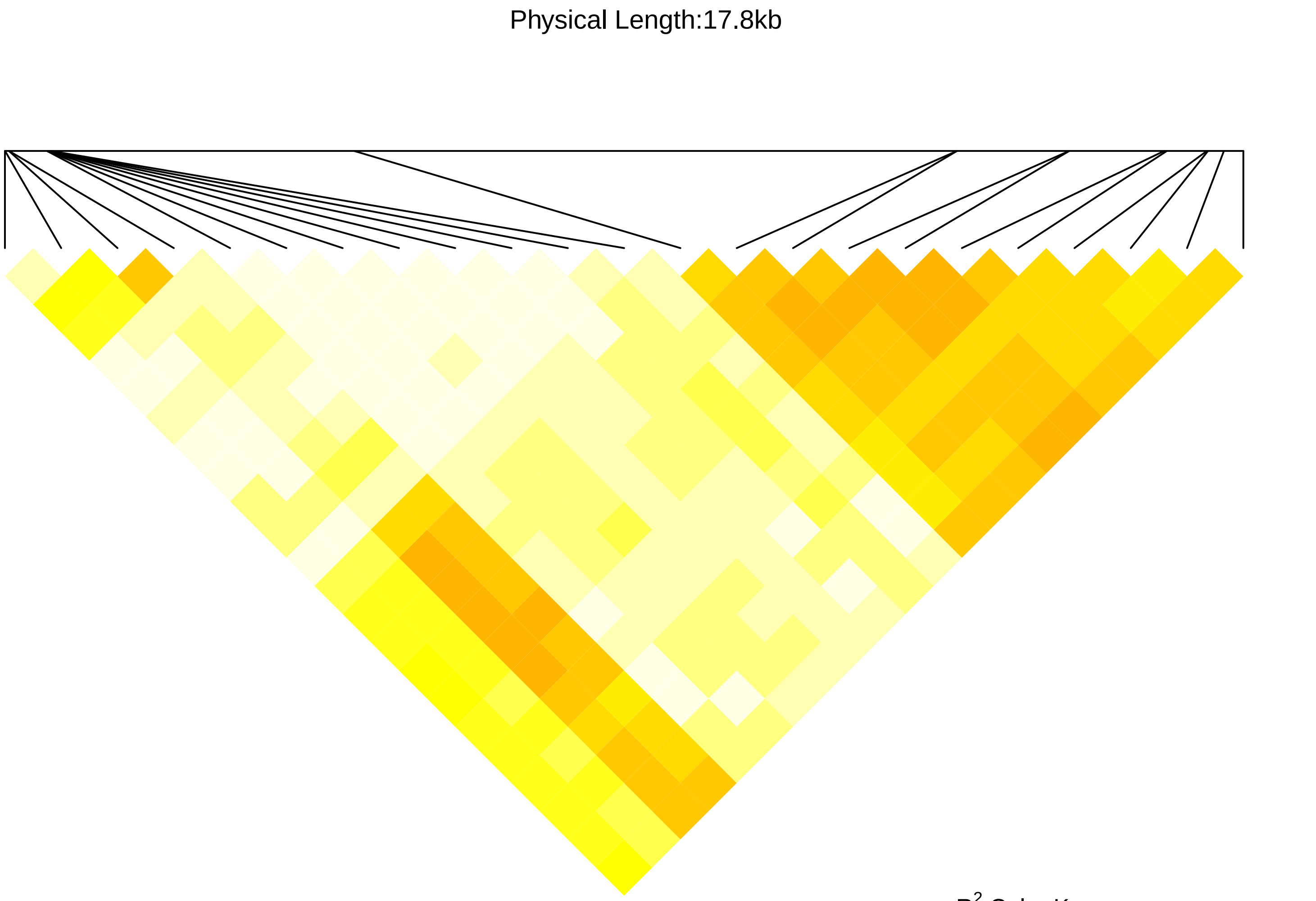

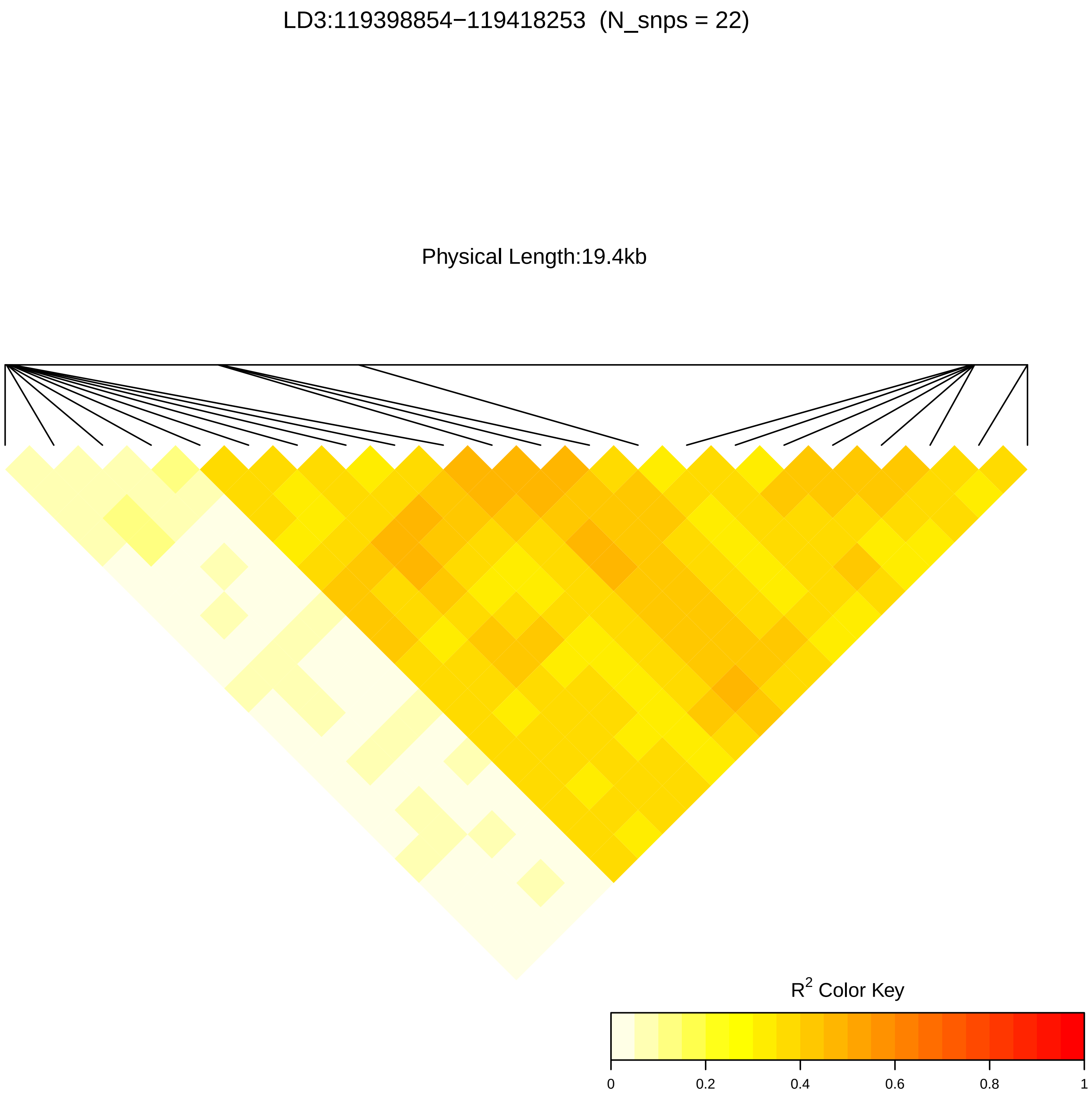
LD3:119398854−119418253 (N_snps = 22)
Physical Length:19.4kb
R2 Color Key
0
0.2
0.4
0.6
0.8
1

LD4:114921−131012 (N_snps = 29)

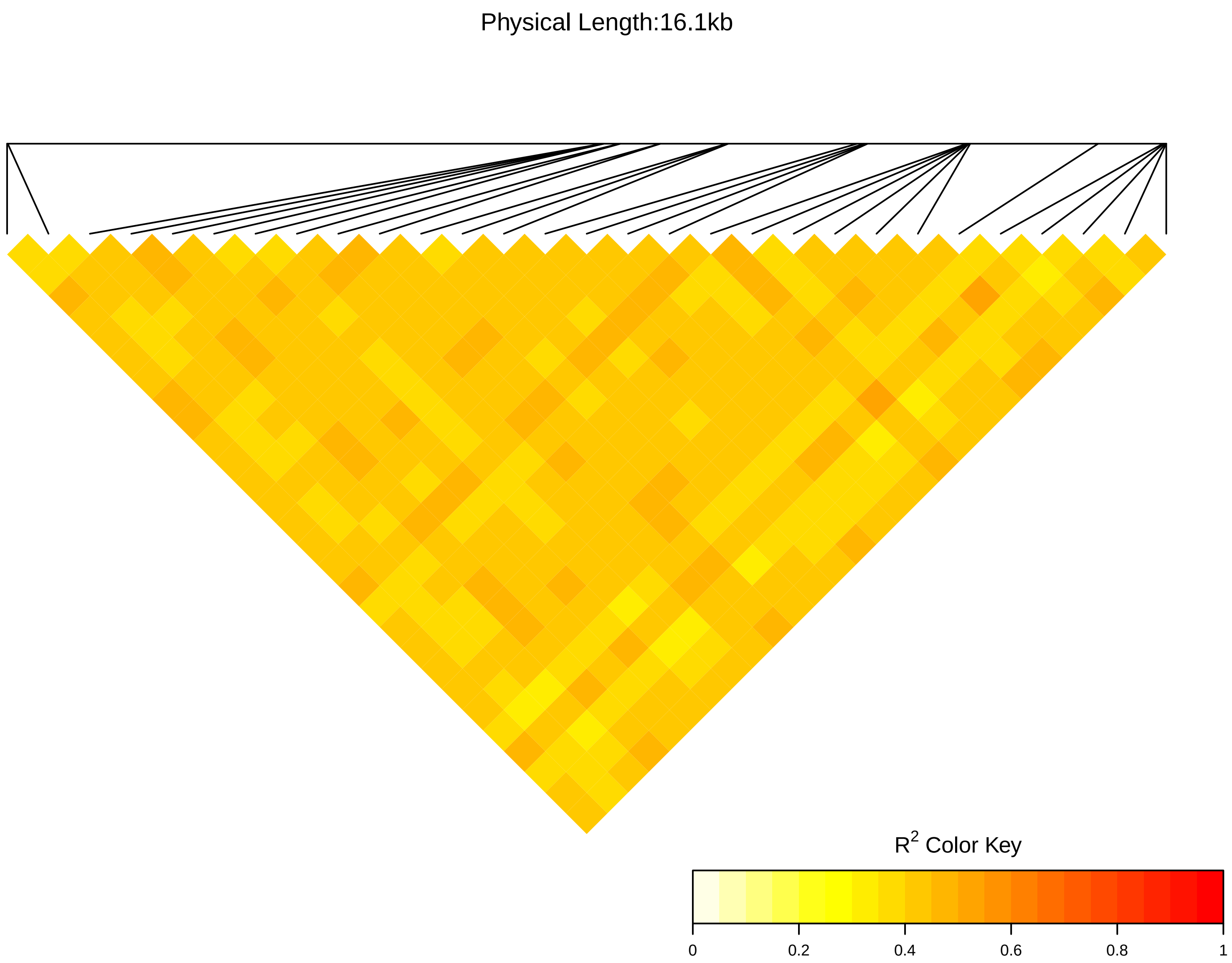

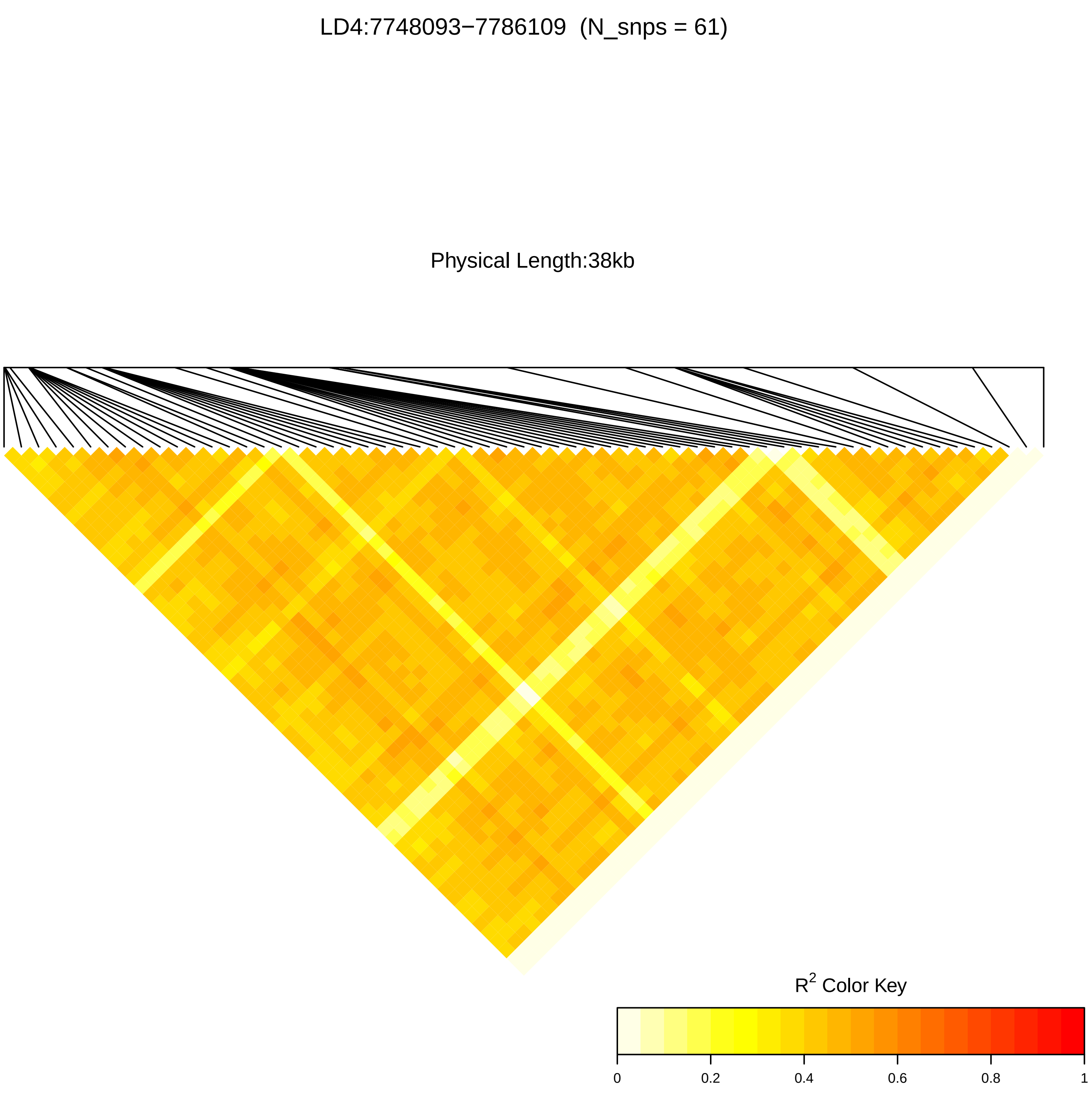

LD4:7748093−7786109 (N_snps = 61)
Physical Length:38kb
R^2 Color Key
0
0.2
0.4
0.6
0.8
1

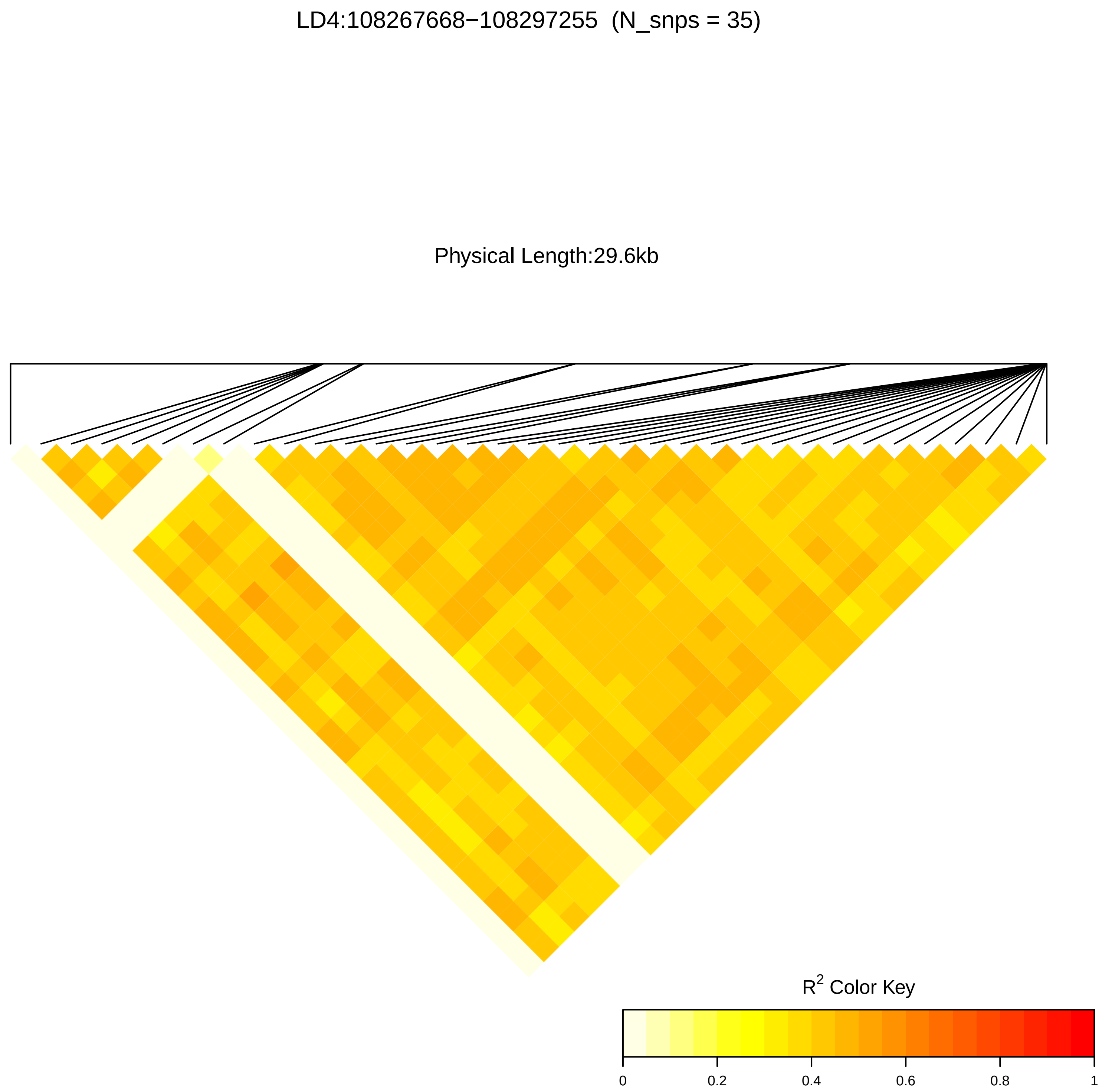
LD4:108267668−108297255 (N_snps = 35)
Physical Length:29.6kb
R^2 Color Key
0
0.2
0.4
0.6
0.8
1

LD5:79520323−79544963 (N_snps = 23)

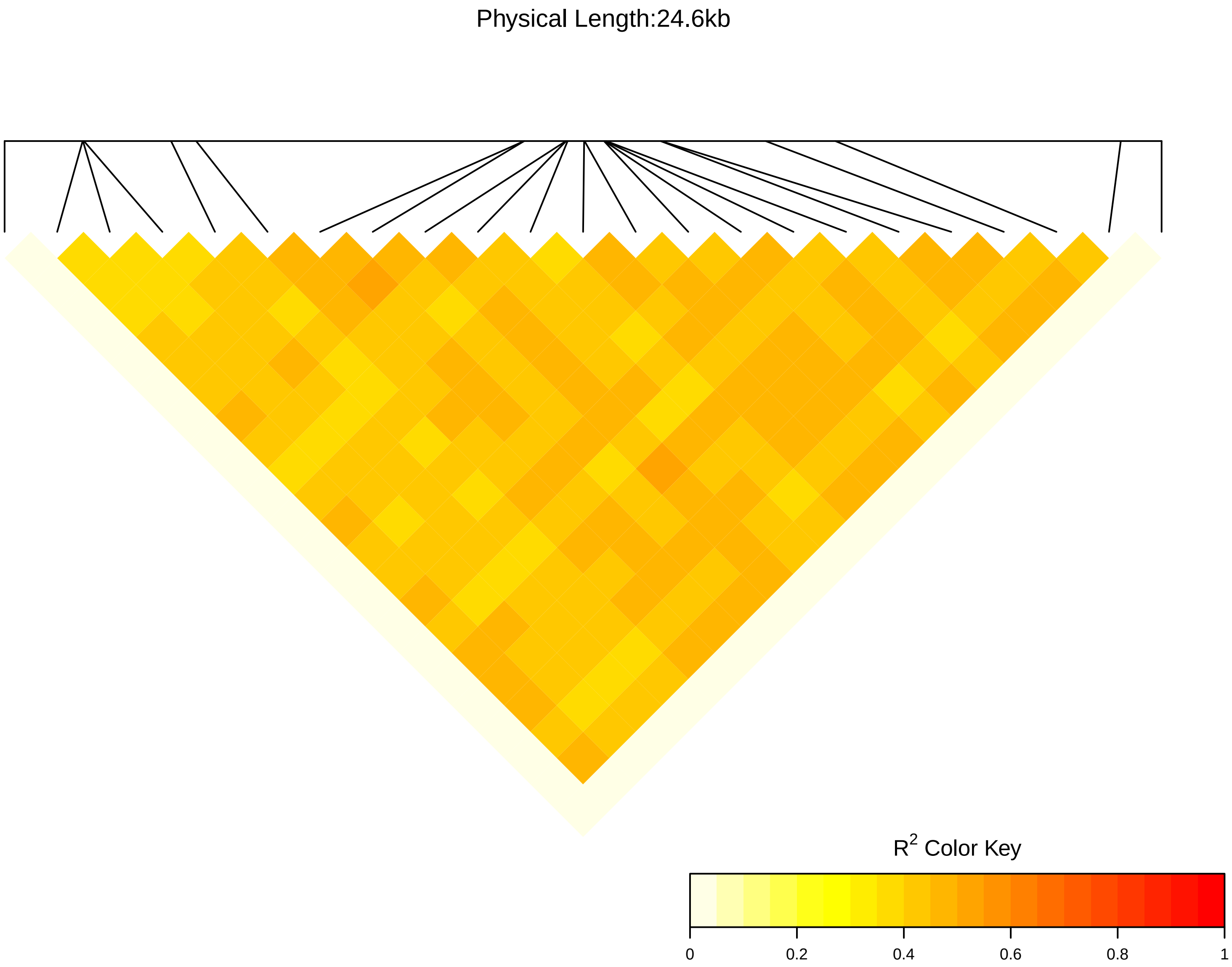

LD6:39744−67663 (N_snps = 32)

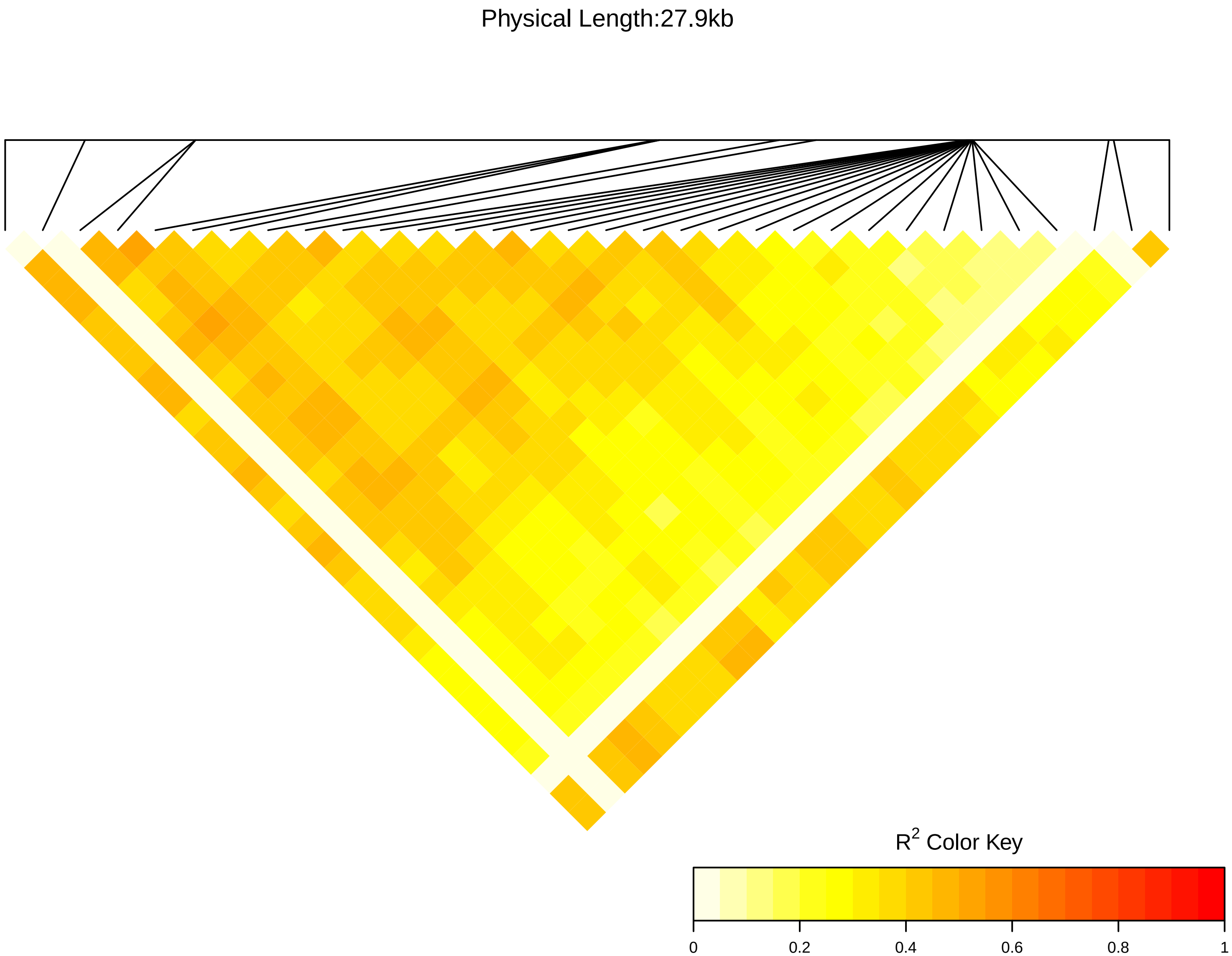

LD6:8997519−9019722 (N_snps = 29)

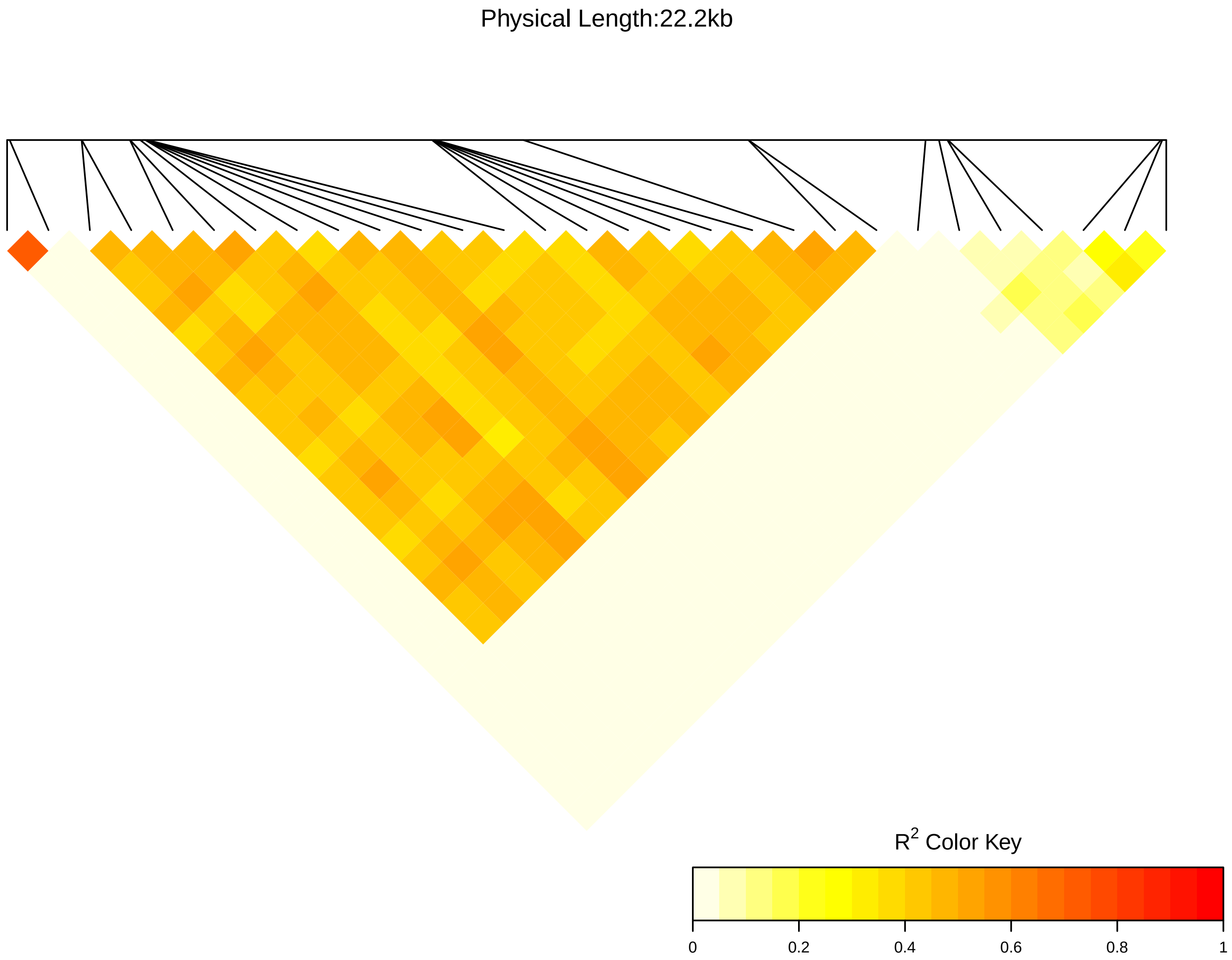

LD7:9986201−9996888 (N_snps = 10)

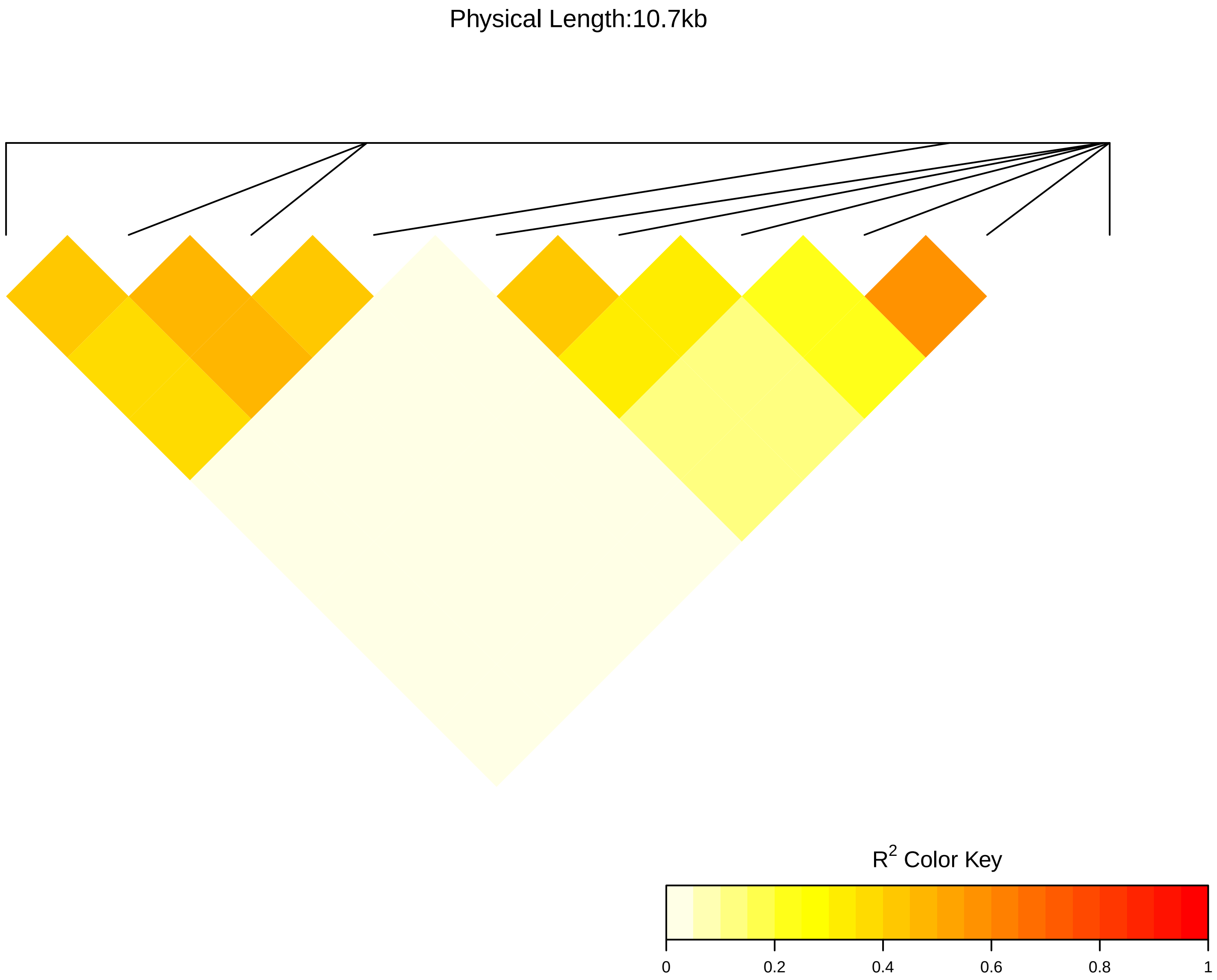

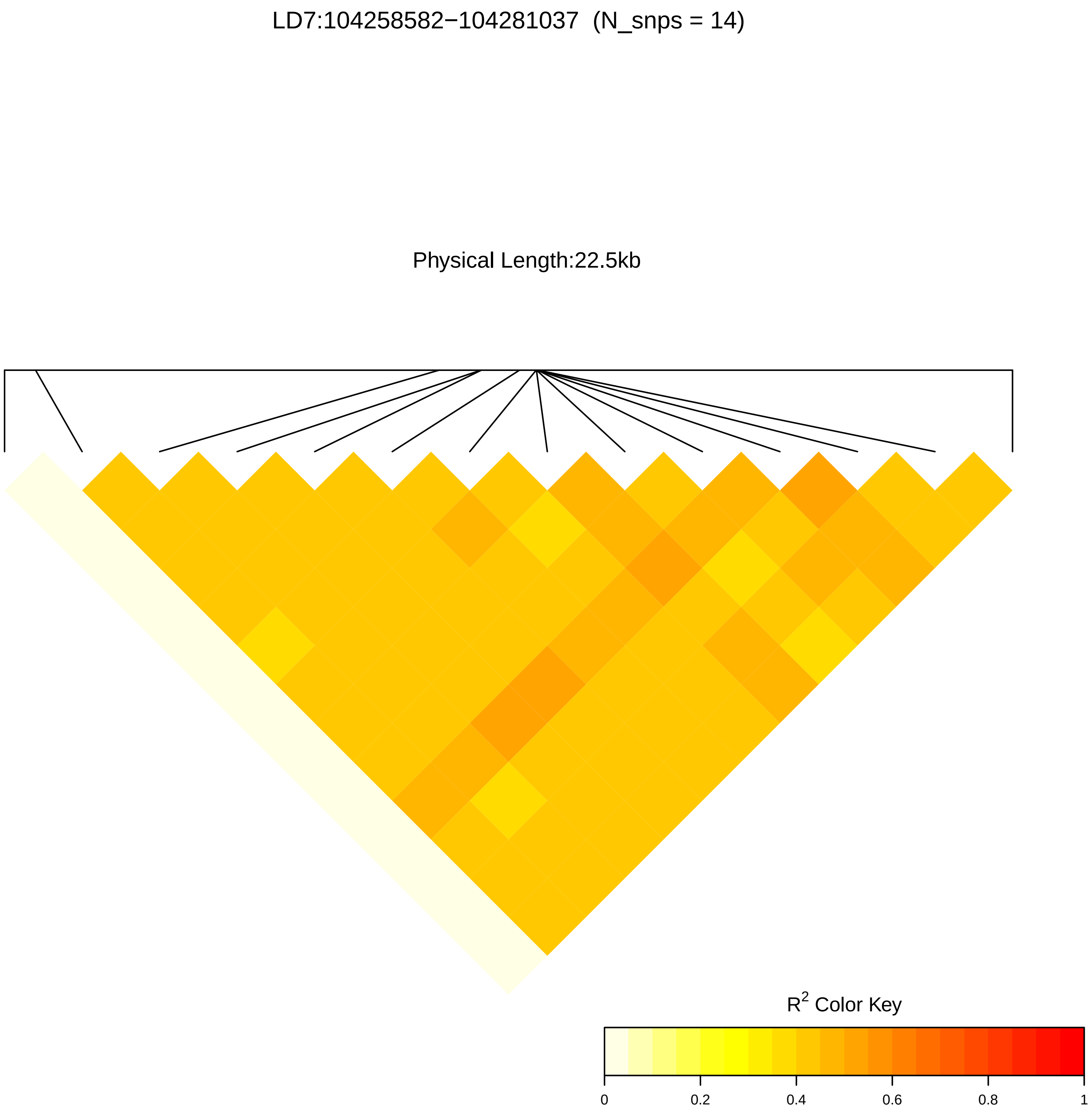
LD7:104258582−104281037 (N_snps = 14)
Physical Length:22.5kb
R^2 Color Key
0
0.2
0.4
0.6
0.8
1

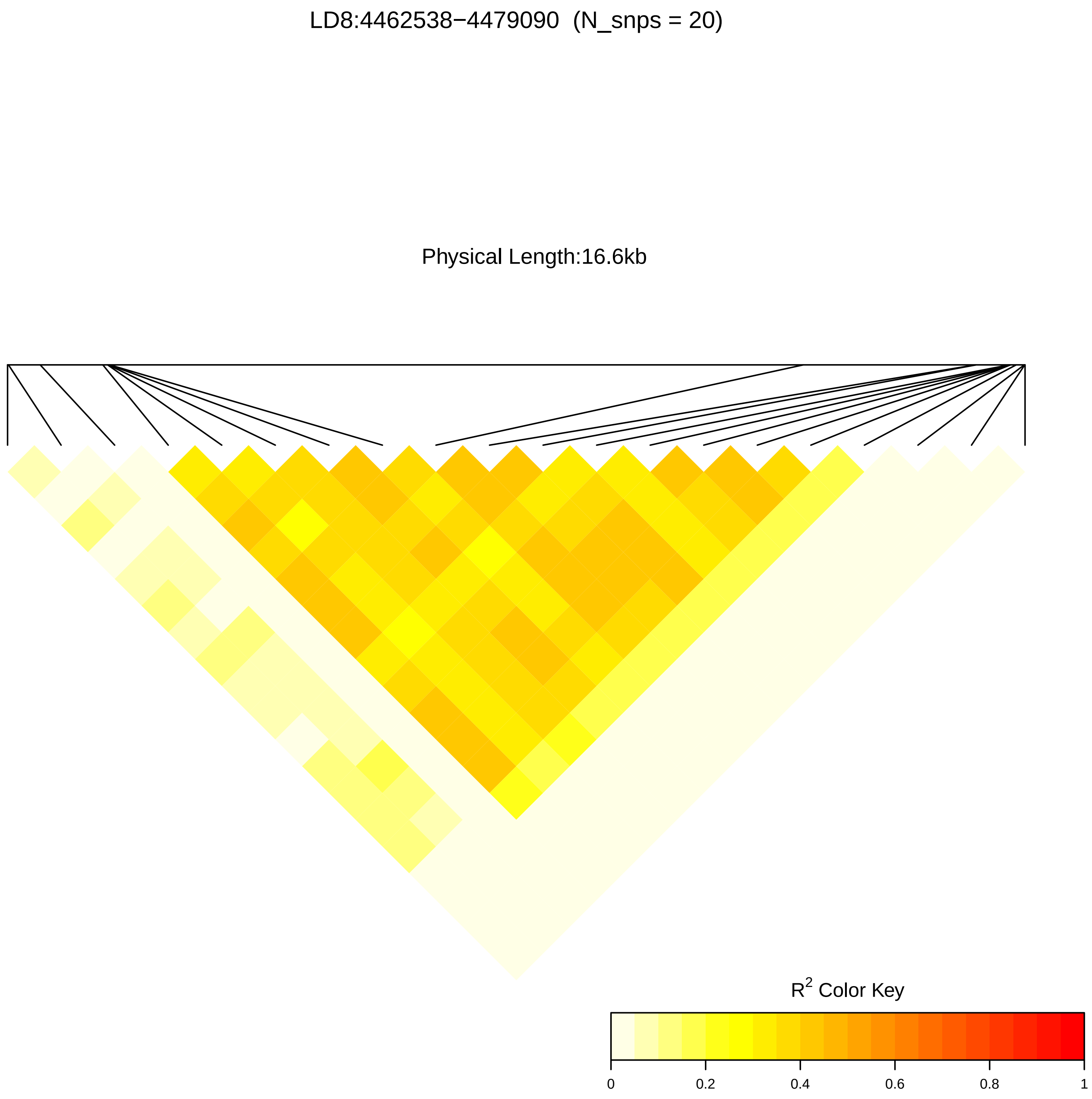
LD8:4462538−4479090 (N_snps = 20)
Physical Length:16.6kb
R2 Color Key
0
0.2
0.4
0.6
0.8
1

LD8:111993409−112004112 (N_snps = 16)

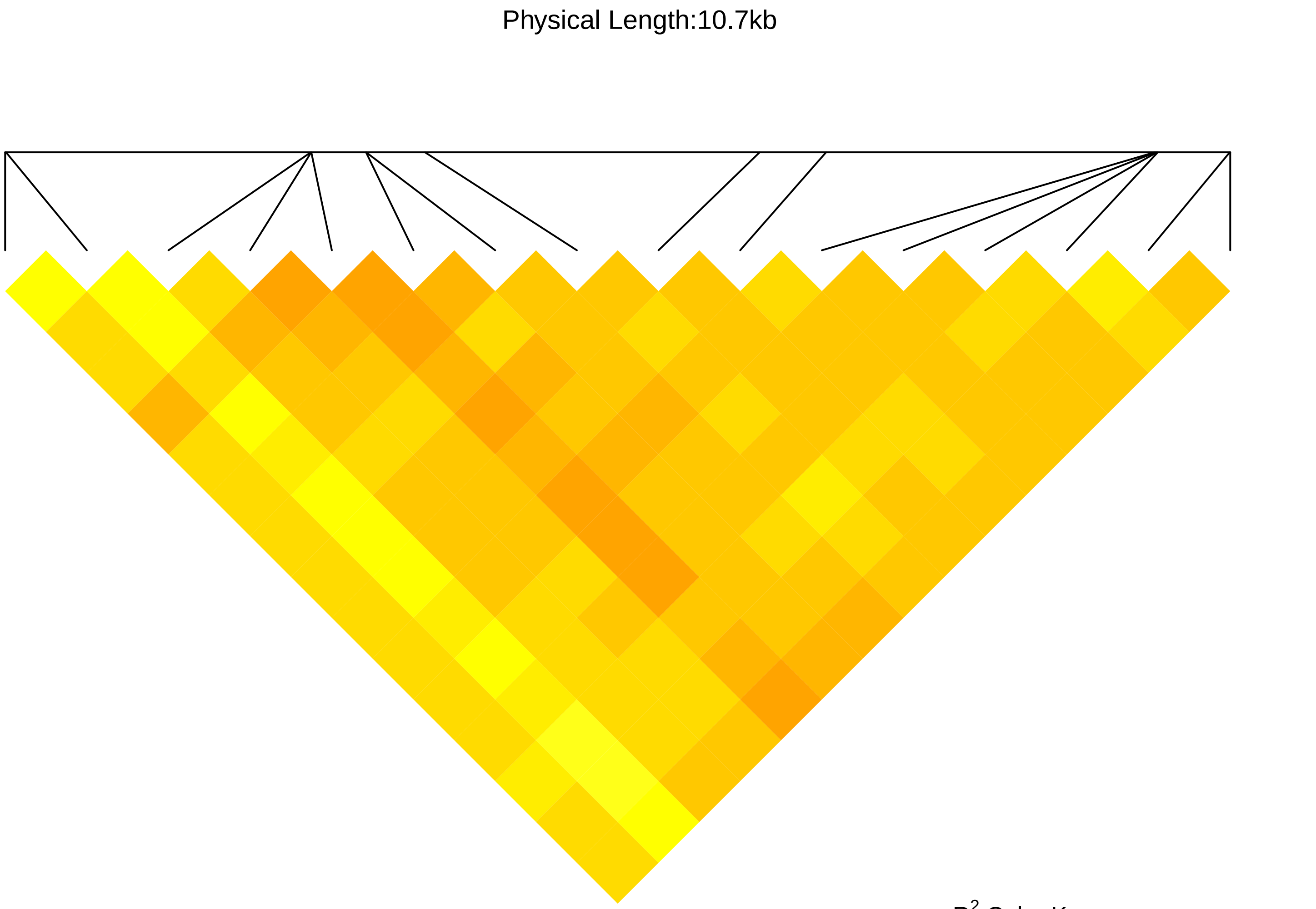

LD9:104119378−104141024 (N_snps = 13)

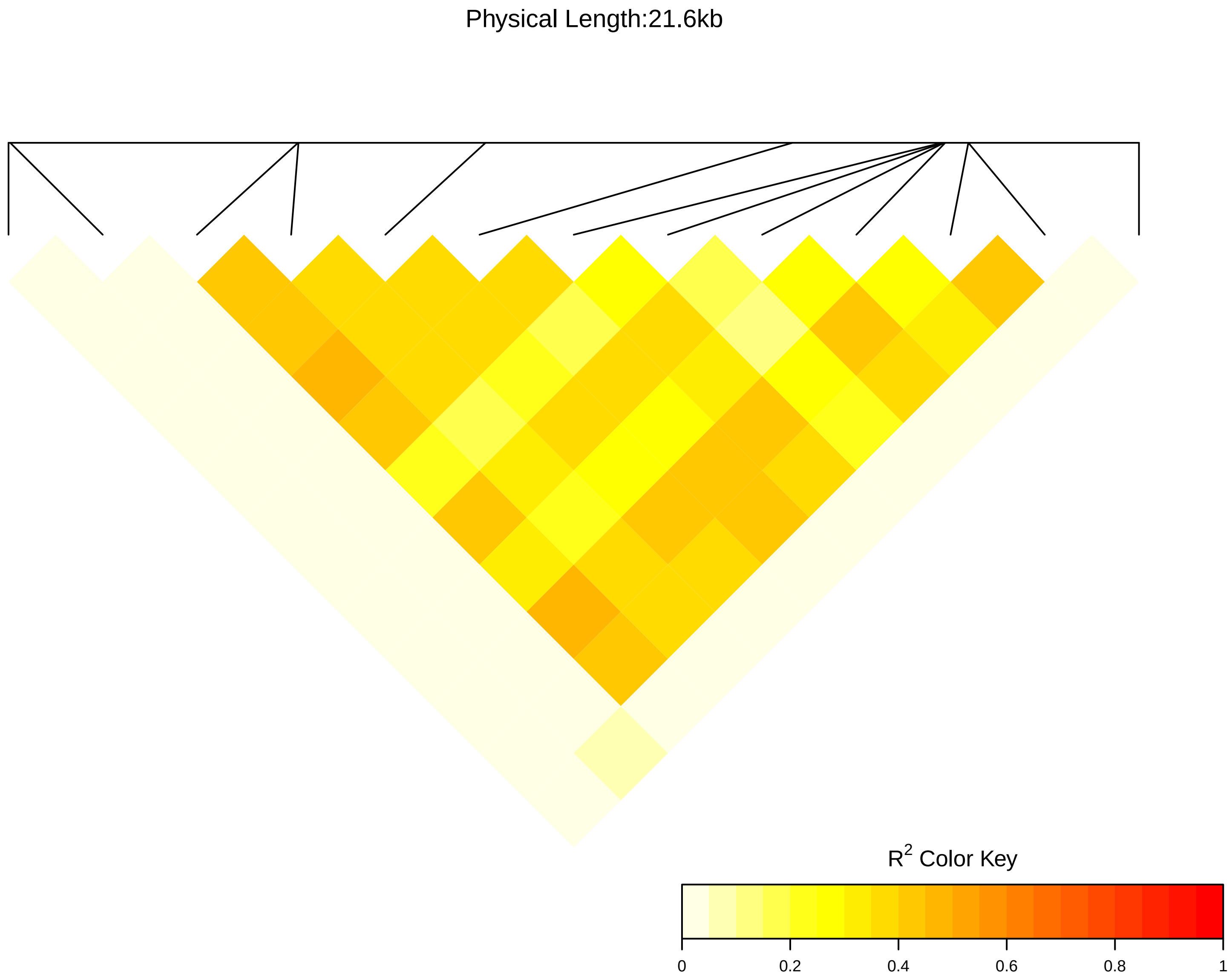

LD10:18852596−18940485 (N_snps = 99)

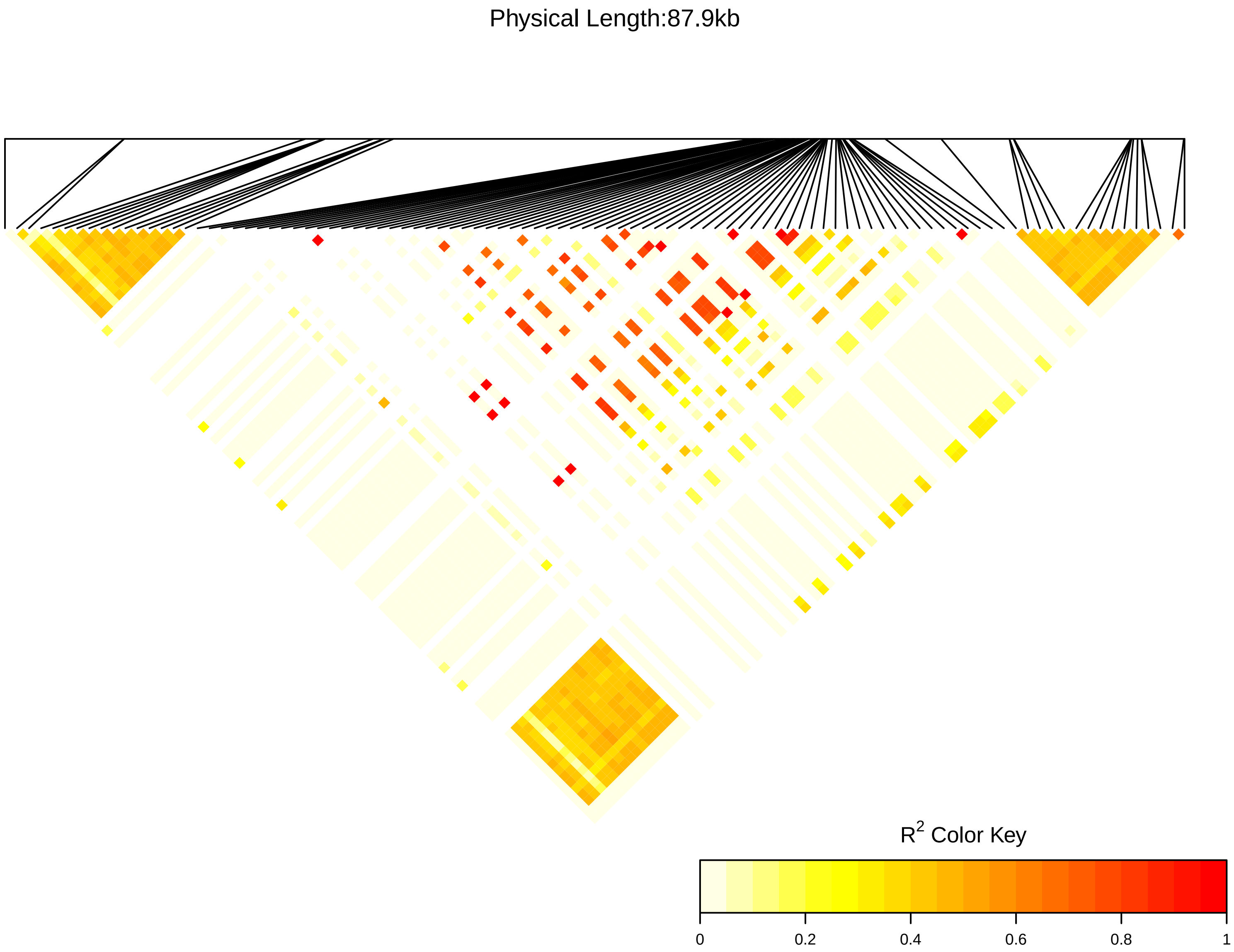

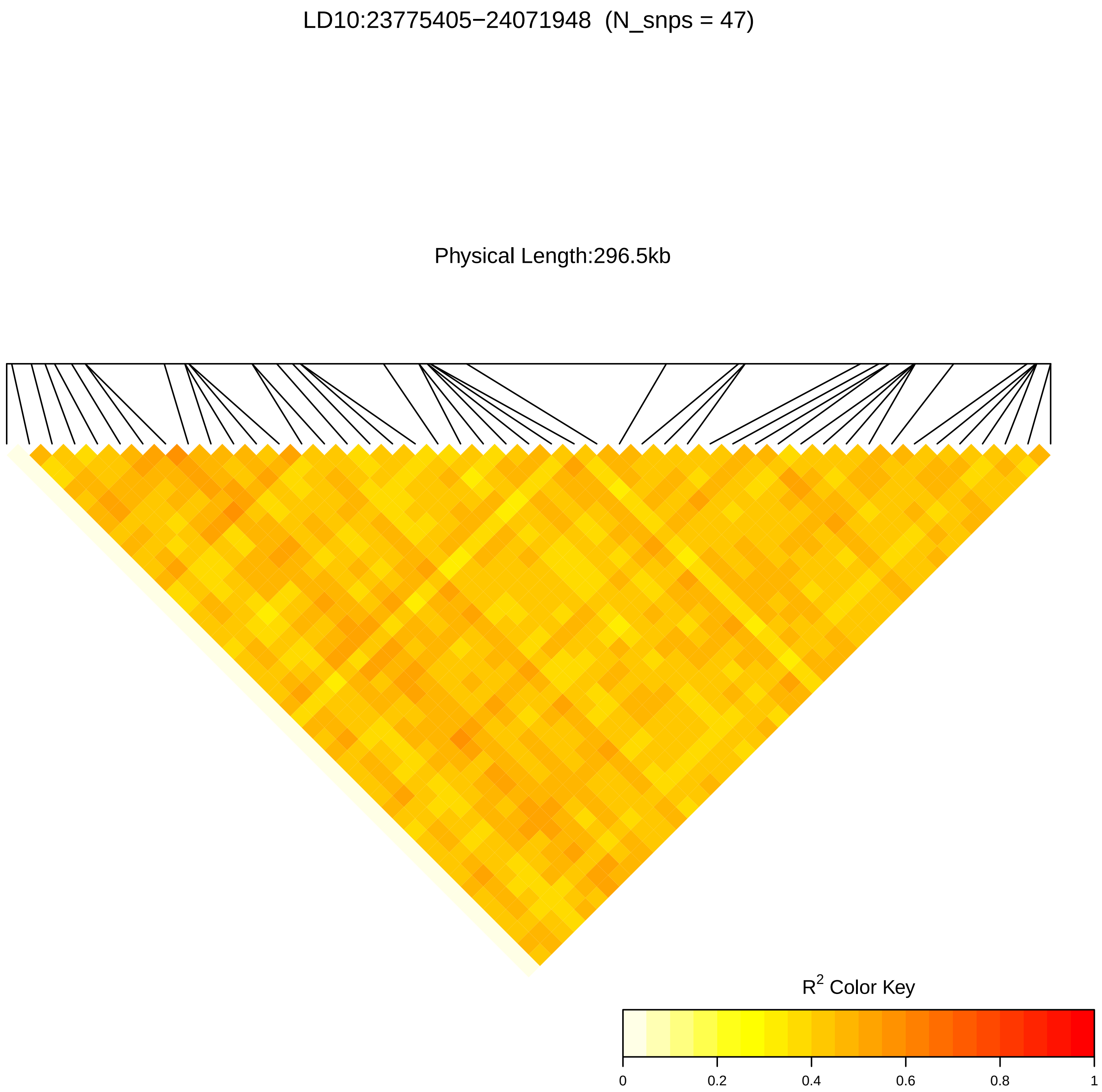

LD10:23775405−24071948 (N_snps = 47)
Physical Length:296.5kb
R2 Color Key
0
0.2
0.4
0.6
0.8
1

LD10:24100254−24459318 (N_snps = 44)

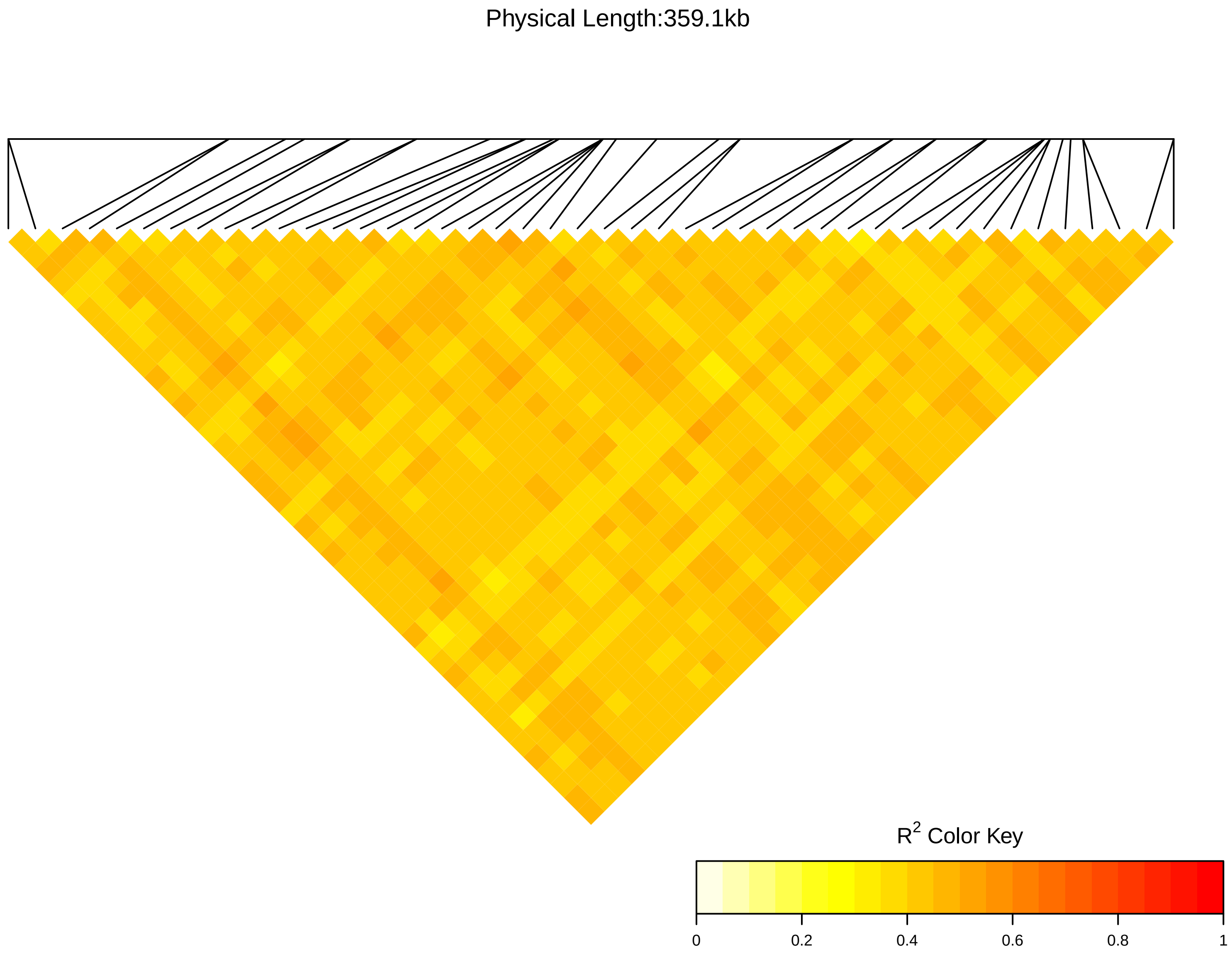

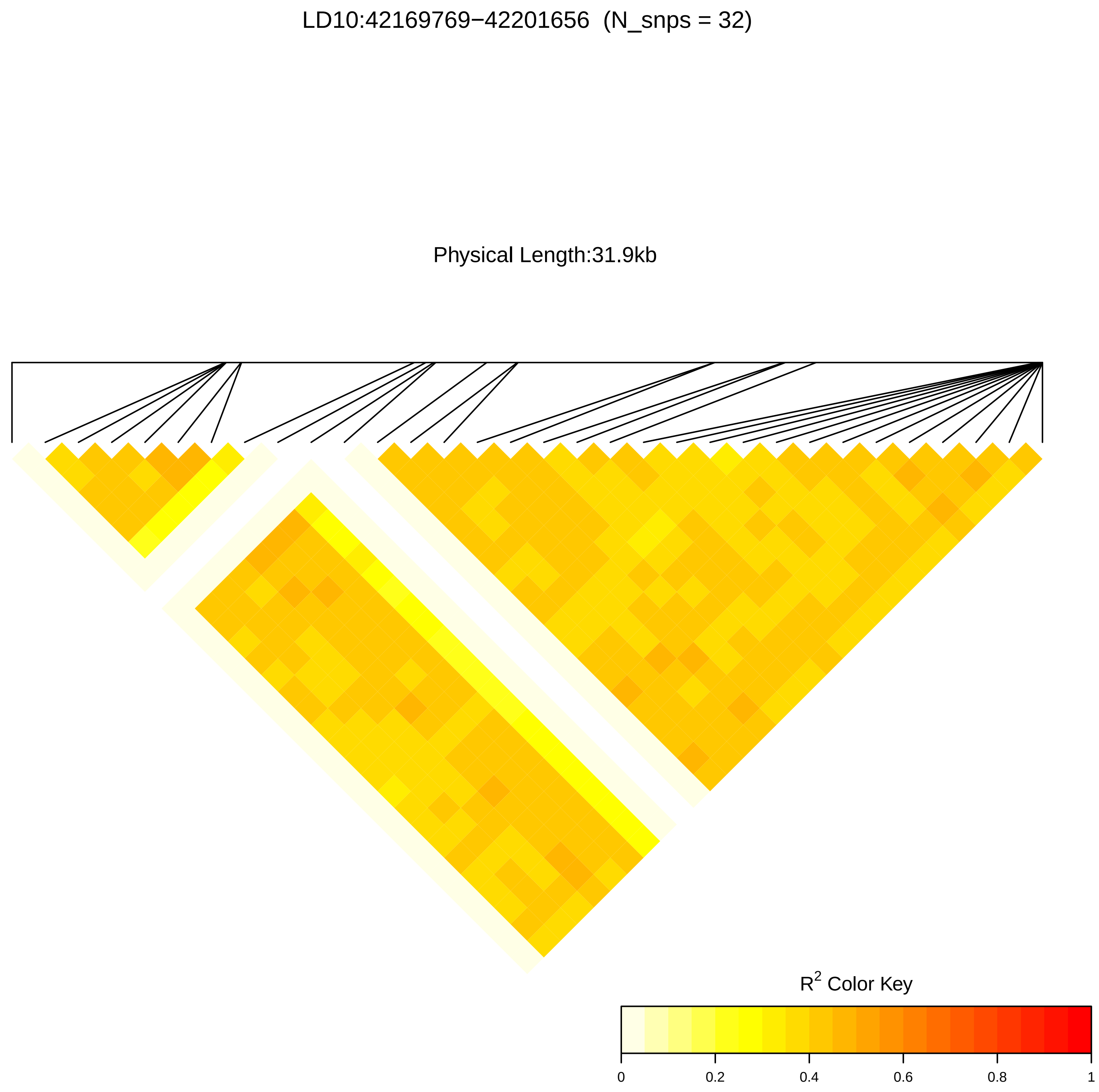
LD10:42169769−42201656 (N_snps = 32)
Physical Length:31.9kb
R^2 Color Key
0
0.2
0.4
0.6
0.8
1

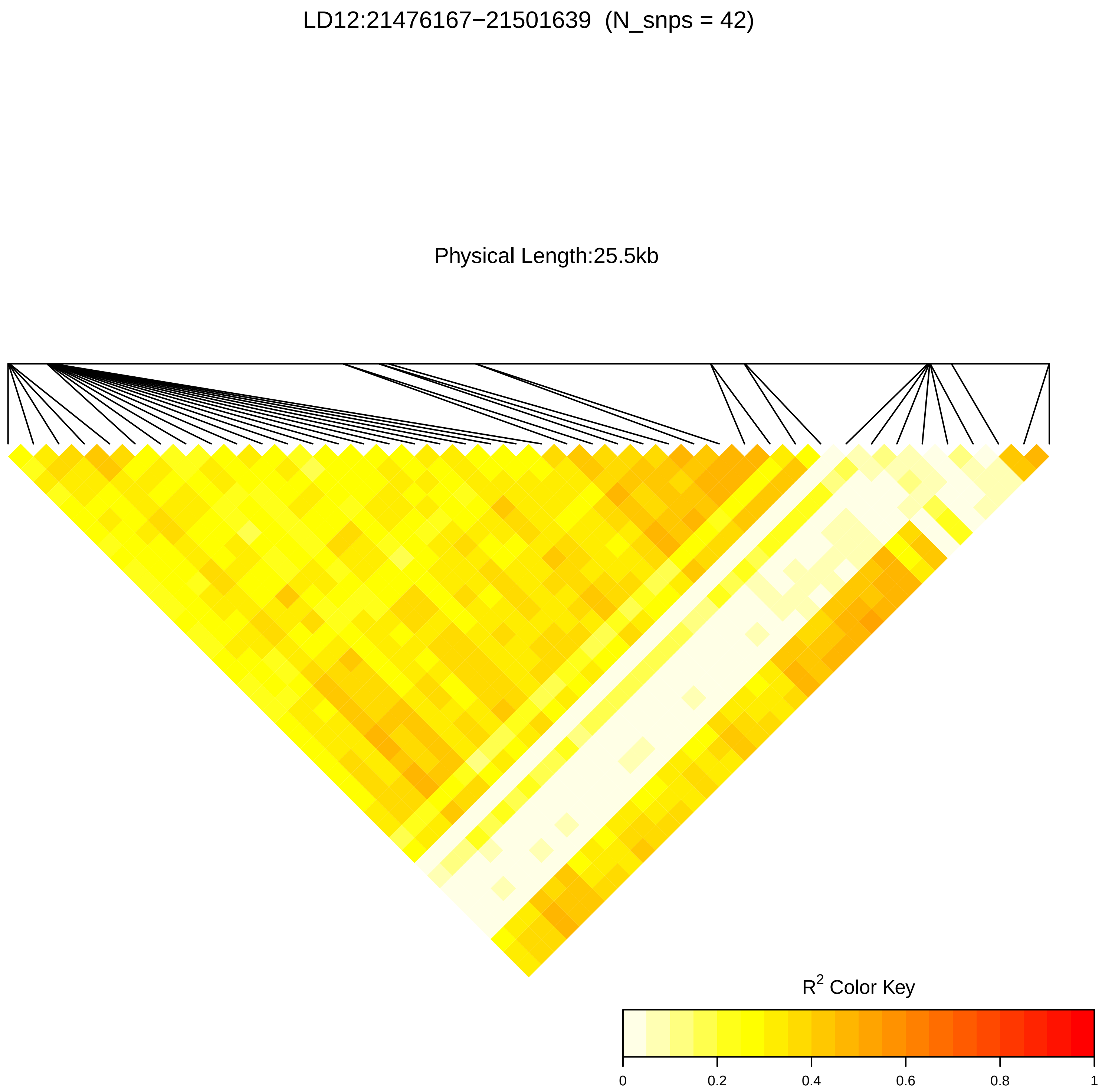

LD12:21476167−21501639 (N_snps = 42)
Physical Length:25.5kb
R2 Color Key
0
0.2
0.4
0.6
0.8
1

LD13:11310352−11334273 (N_snps = 40)

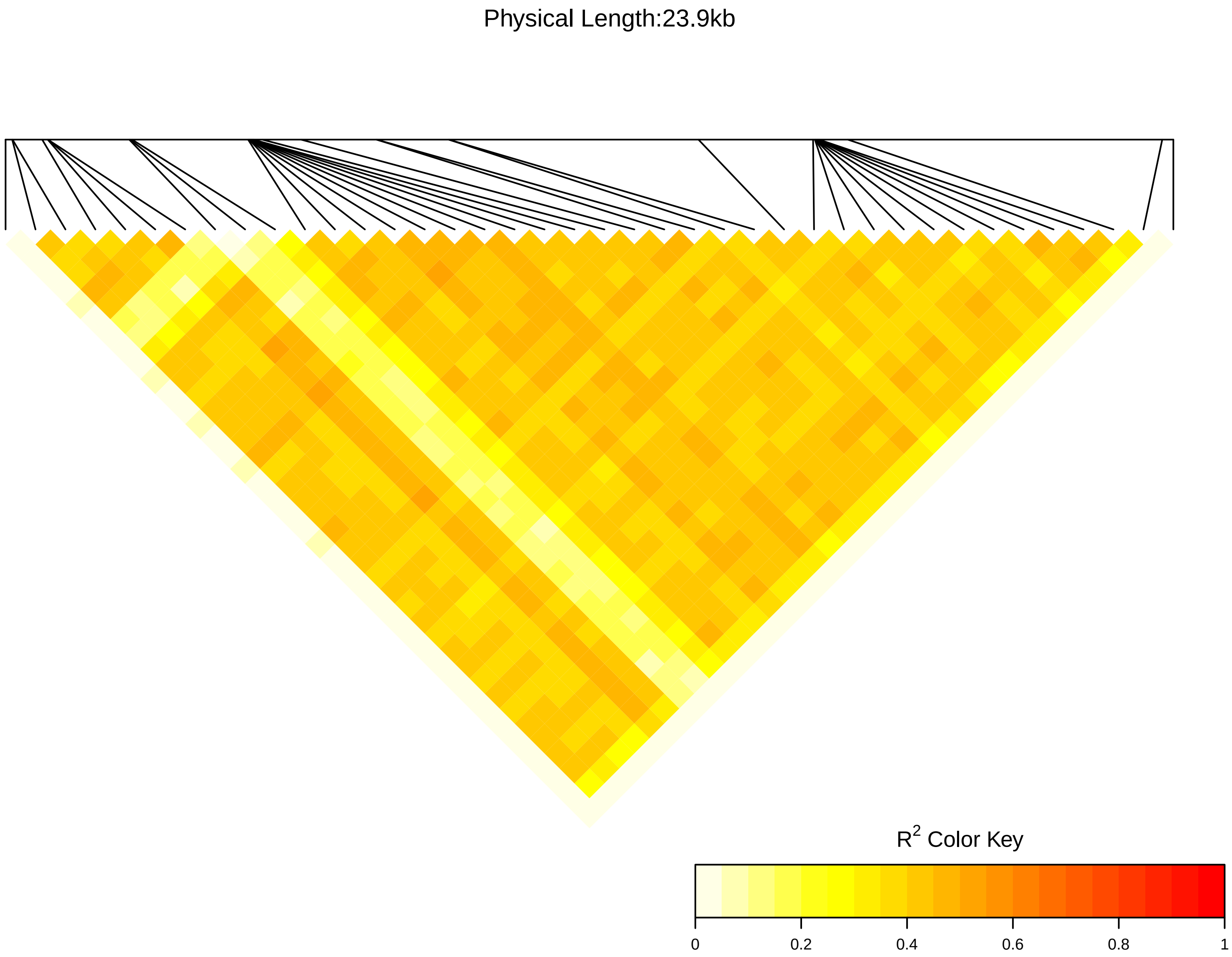

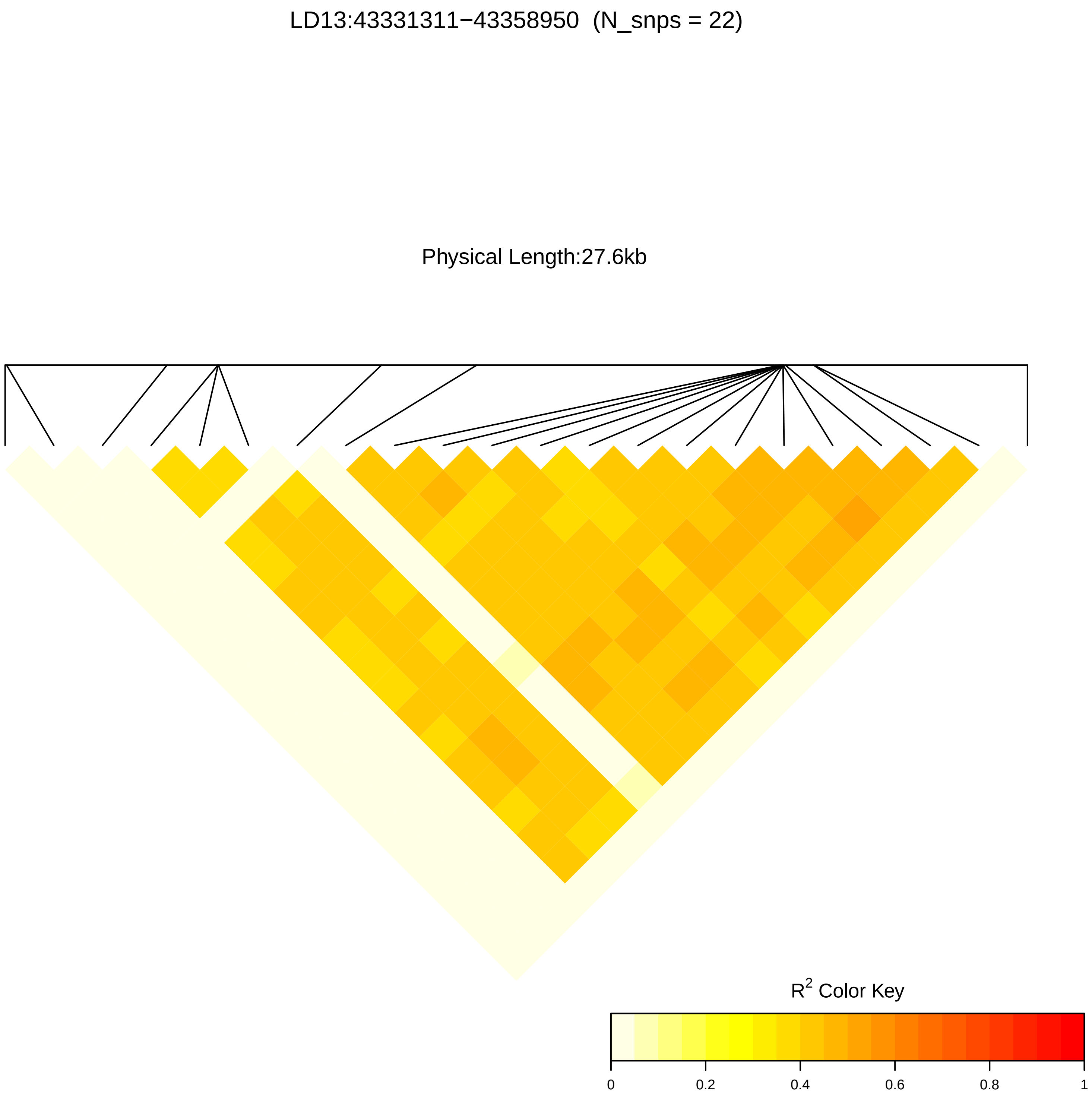
LD13:43331311−43358950 (N_snps = 22)
Physical Length:27.6kb
R2 Color Key
0
0.2
0.4
0.6
0.8
1

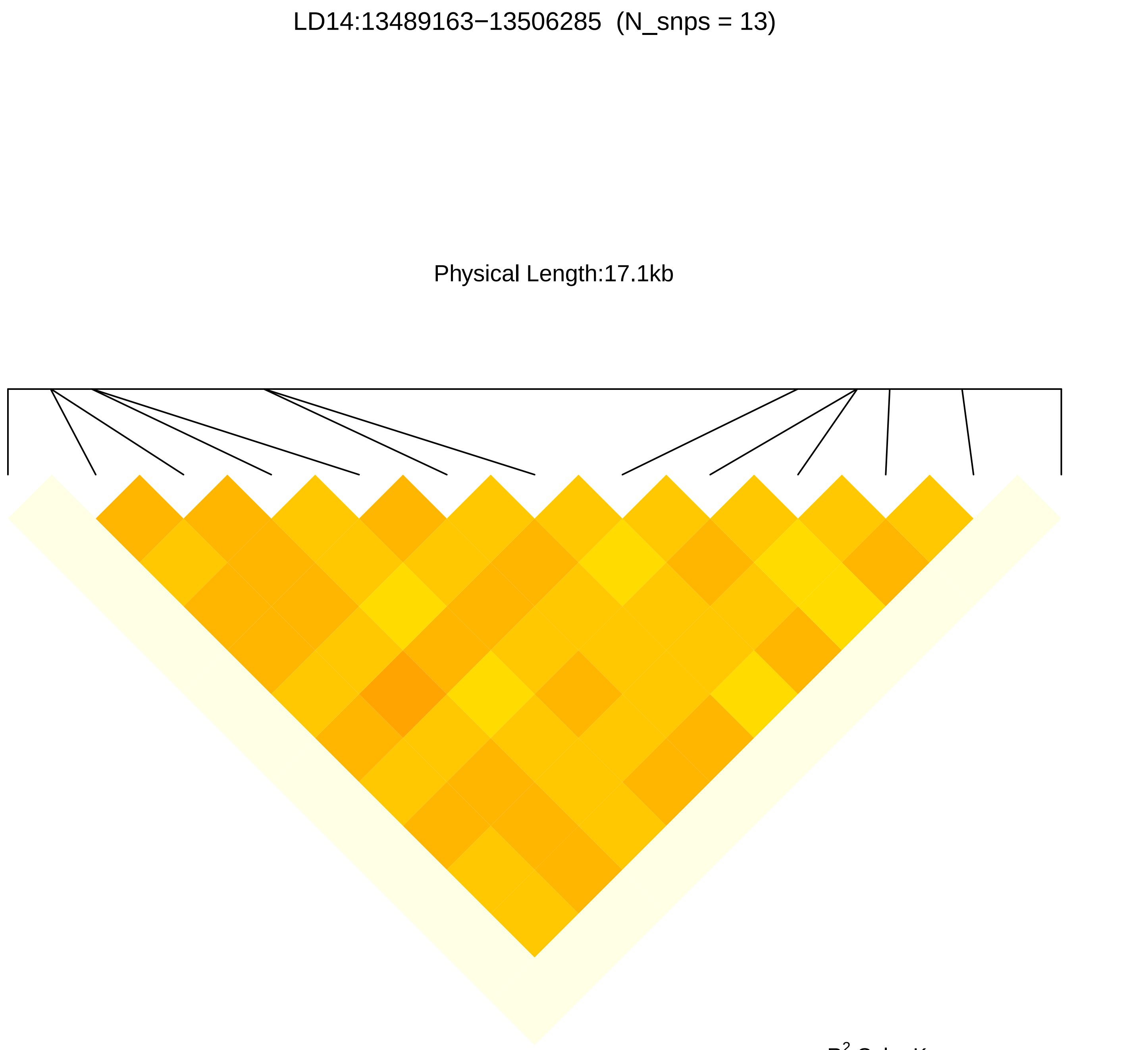
LD14:13489163−13506285 (N_snps = 13)
Physical Length:17.1kb
R2 Color Key
0
0.2
0.4
0.6
0.8
1

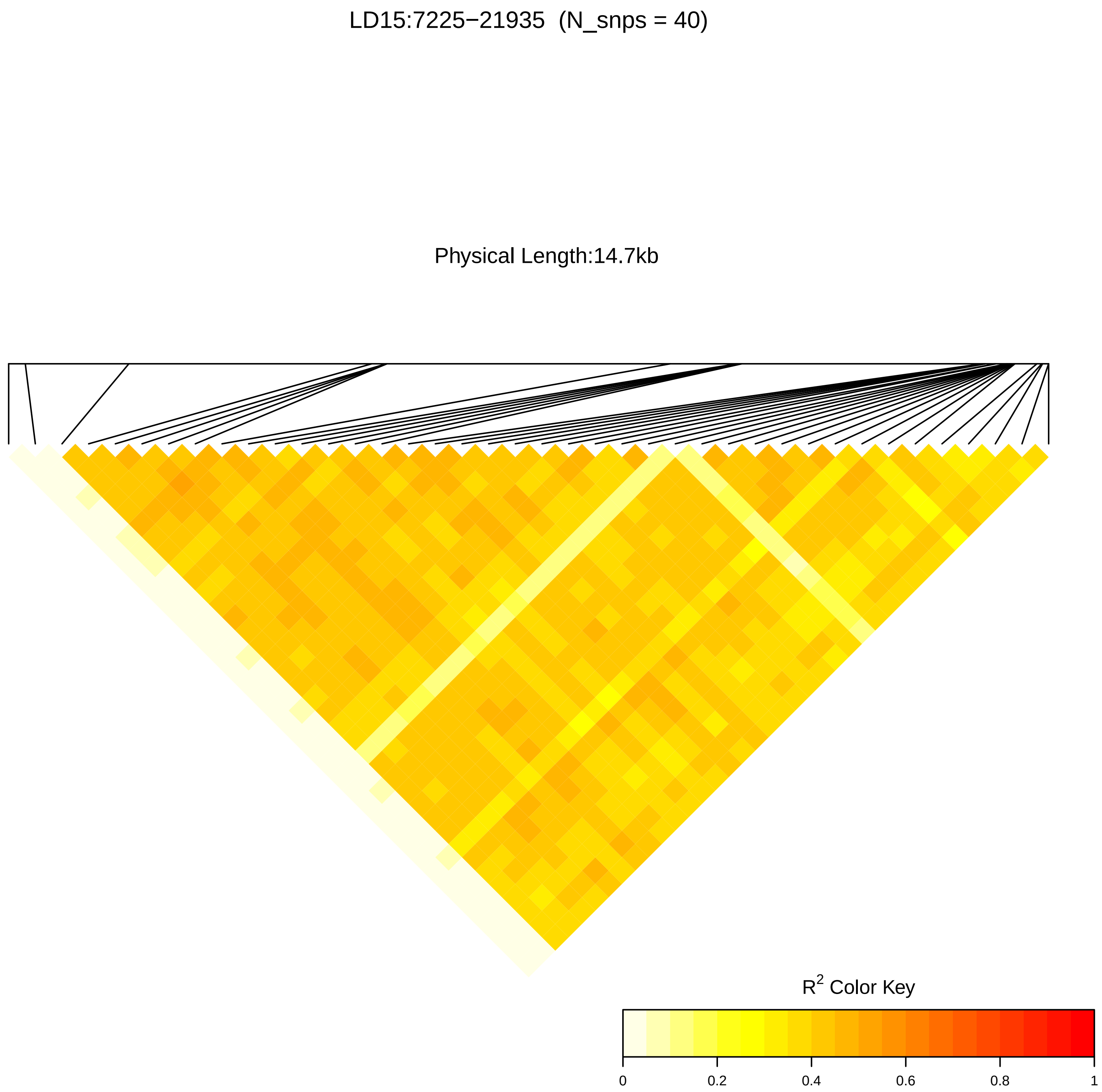

LD15:7225−21935 (N_snps = 40)
Physical Length:14.7kb
R^2 Color Key
0
0.2
0.4
0.6
0.8
1

LD15:12265252−12276097 (N_snps = 11)

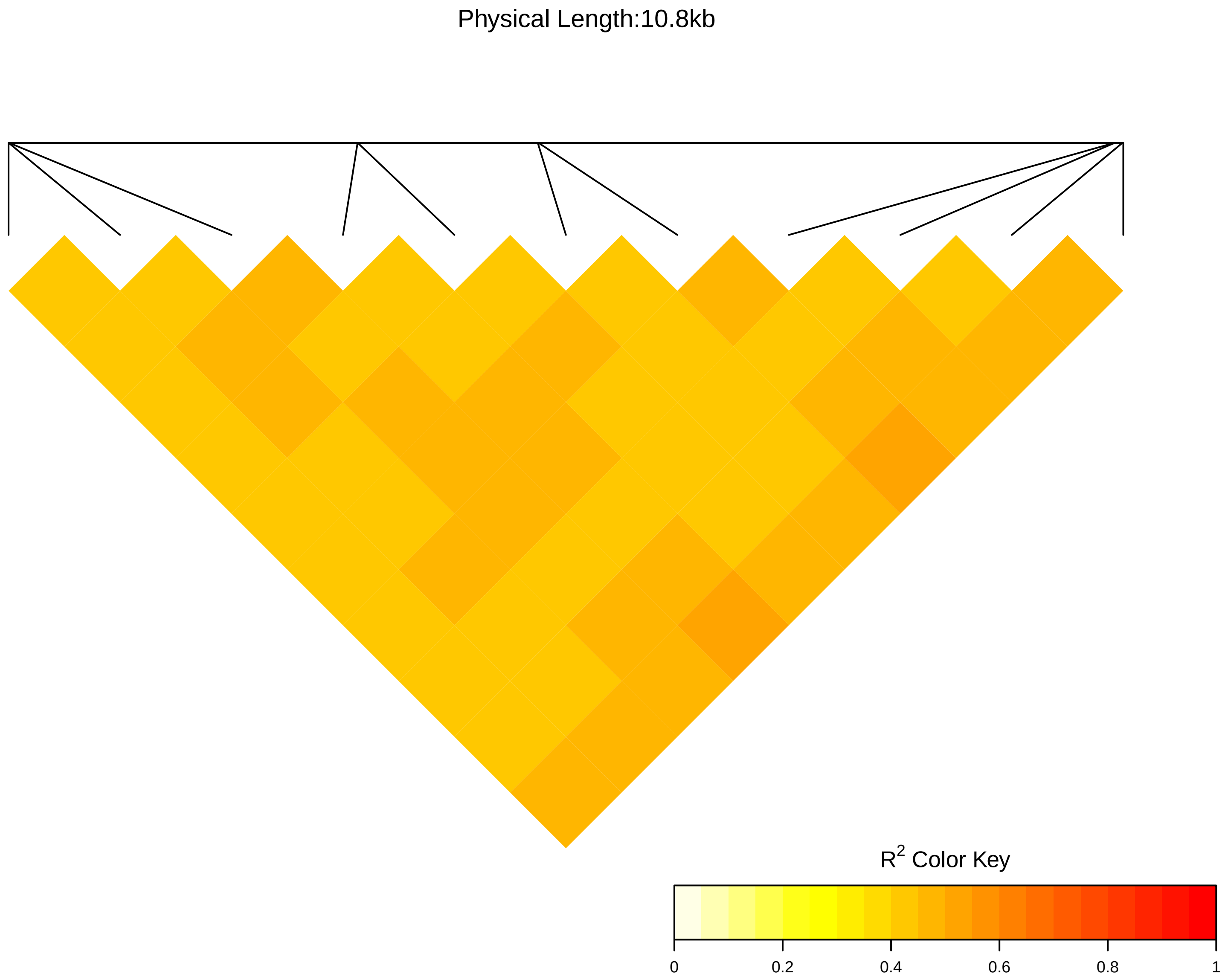

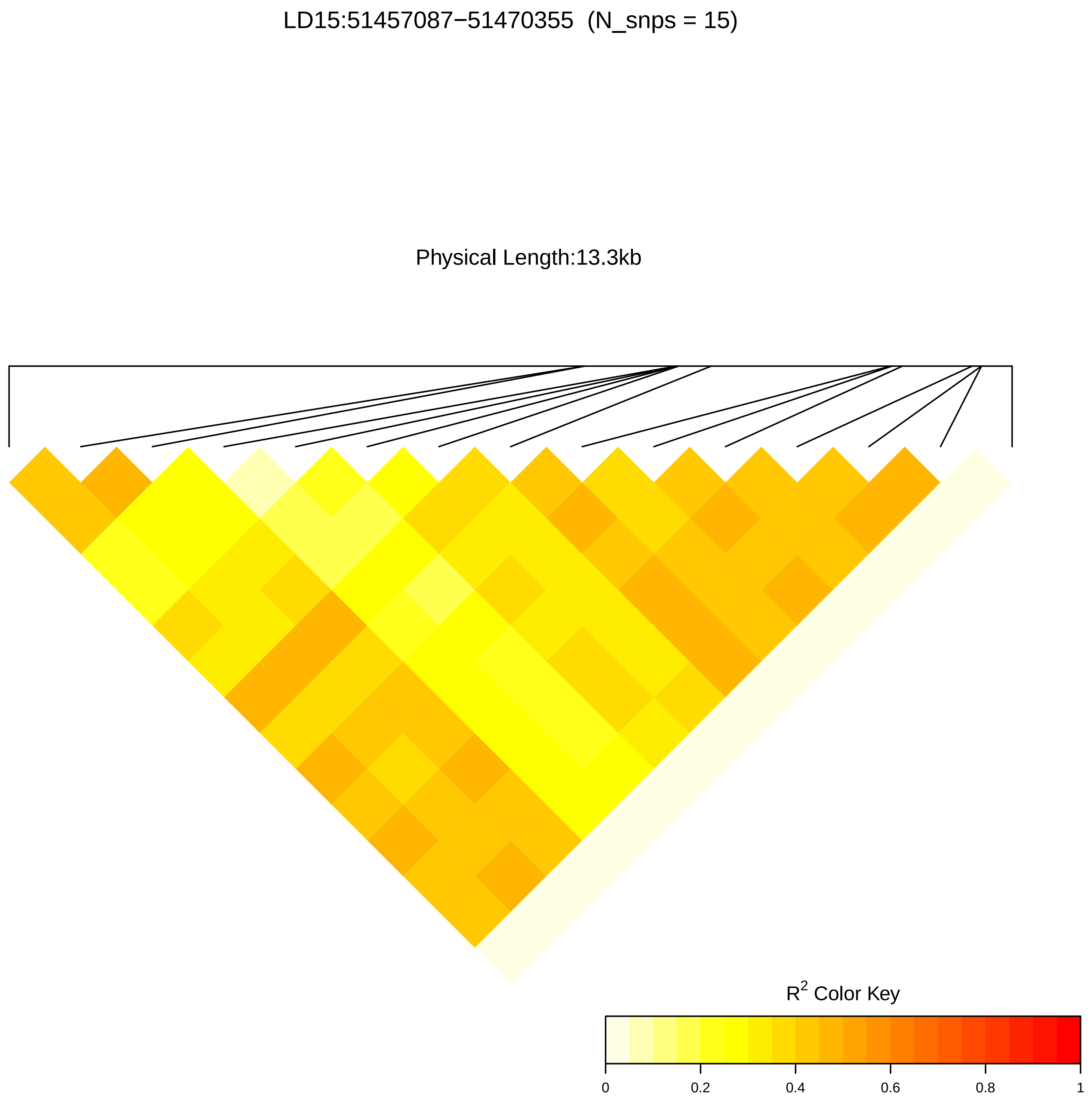
LD15:51457087−51470355 (N_snps = 15)
Physical Length:13.3kb
R2 Color Key
0
0.2
0.4
0.6
0.8
1

LD17:68057715−68070742 (N_snps = 11)

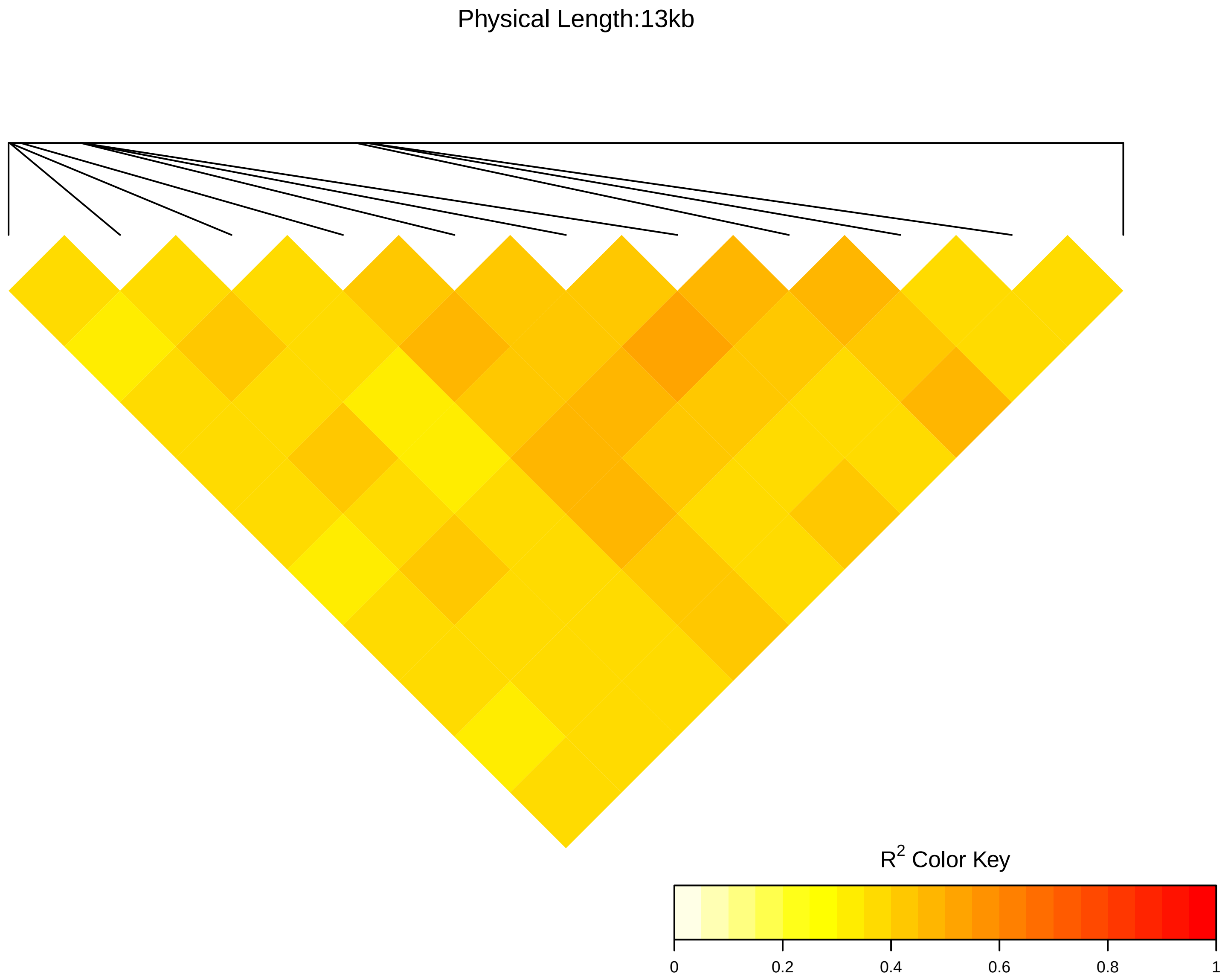

LD17:71143576−71169181 (N_snps = 36)

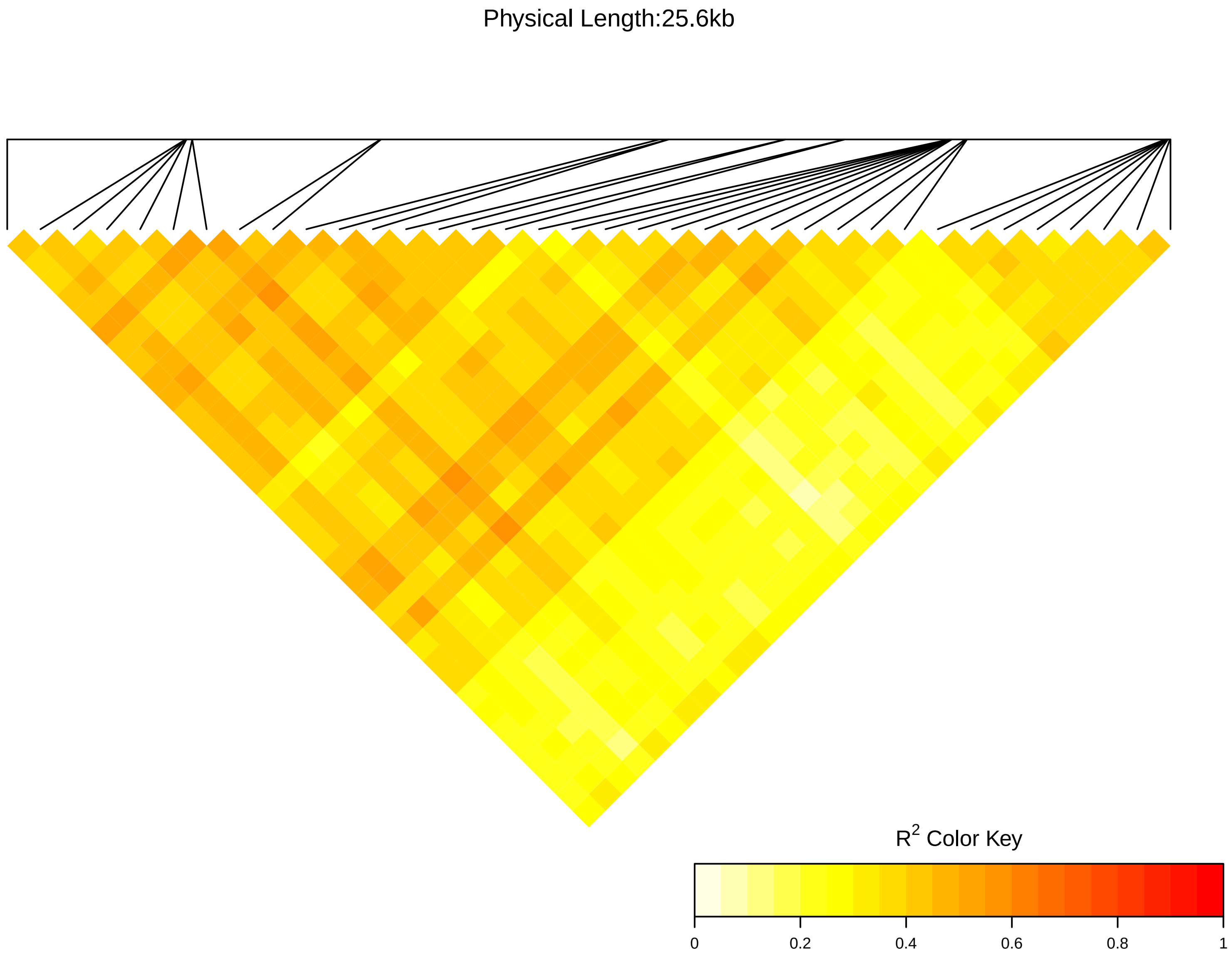

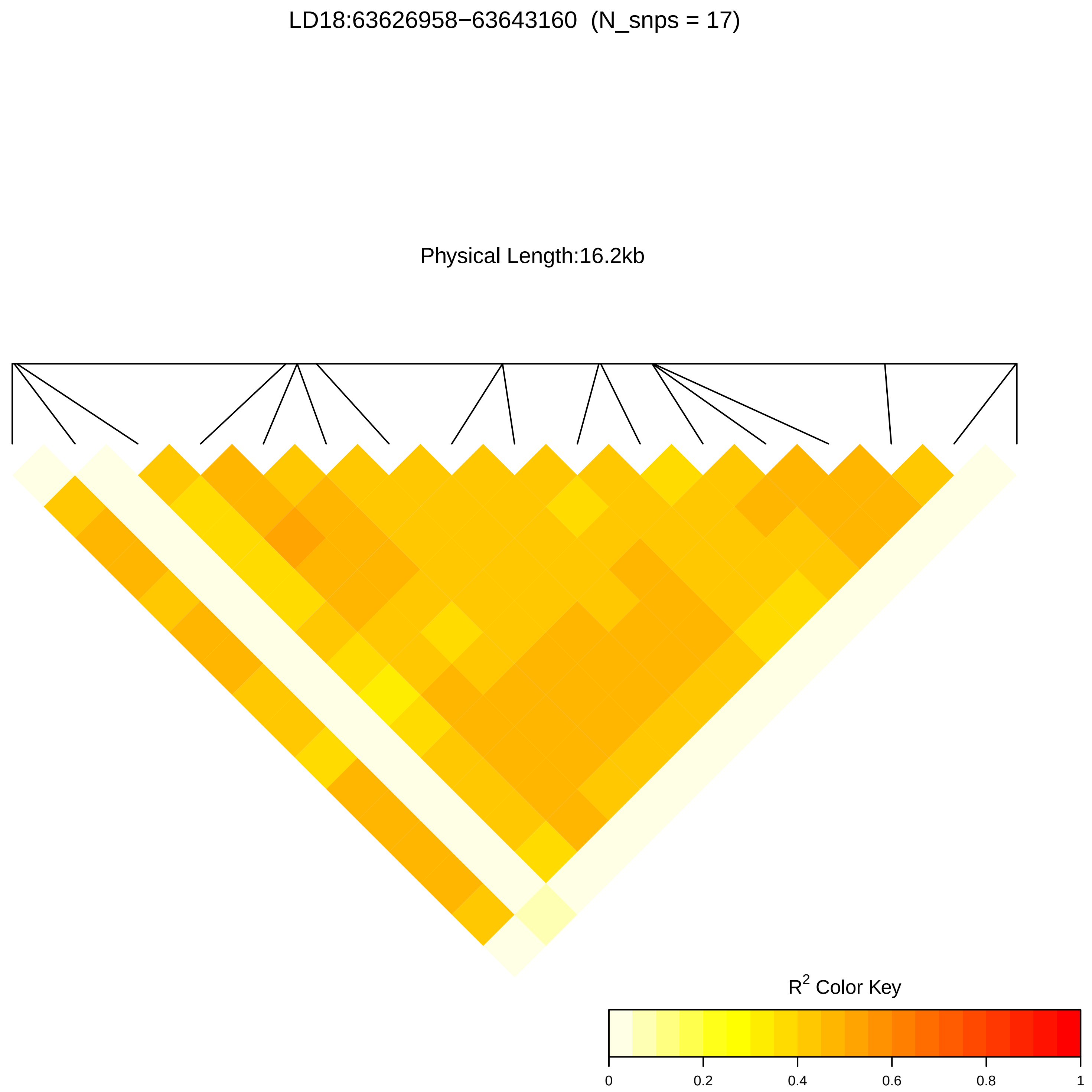
LD18:63626958−63643160 (N_snps = 17)
Physical Length:16.2kb
R^2 Color Key
0
0.2
0.4
0.6
0.8
1

LD19:31258759−31290592 (N_snps = 27)

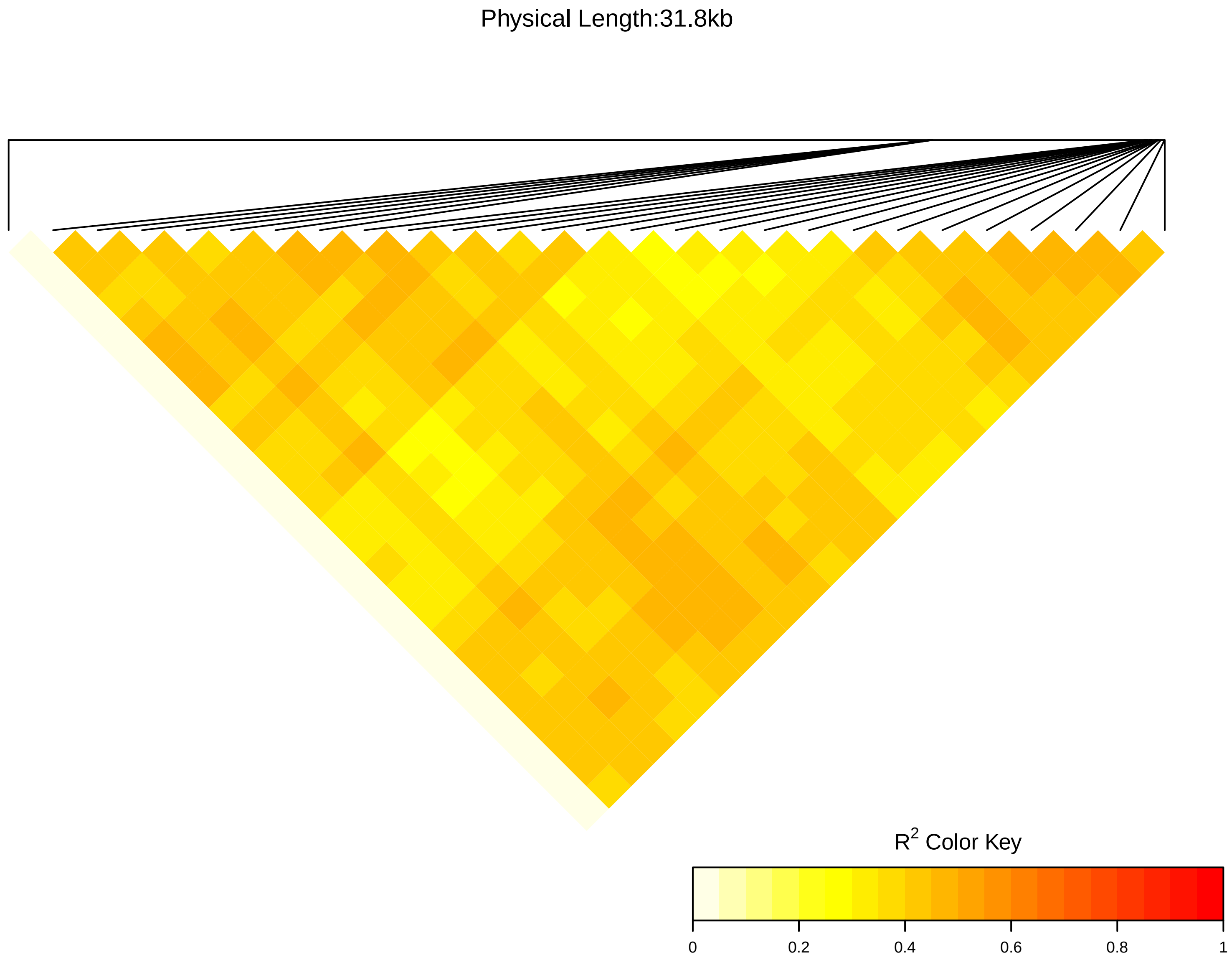

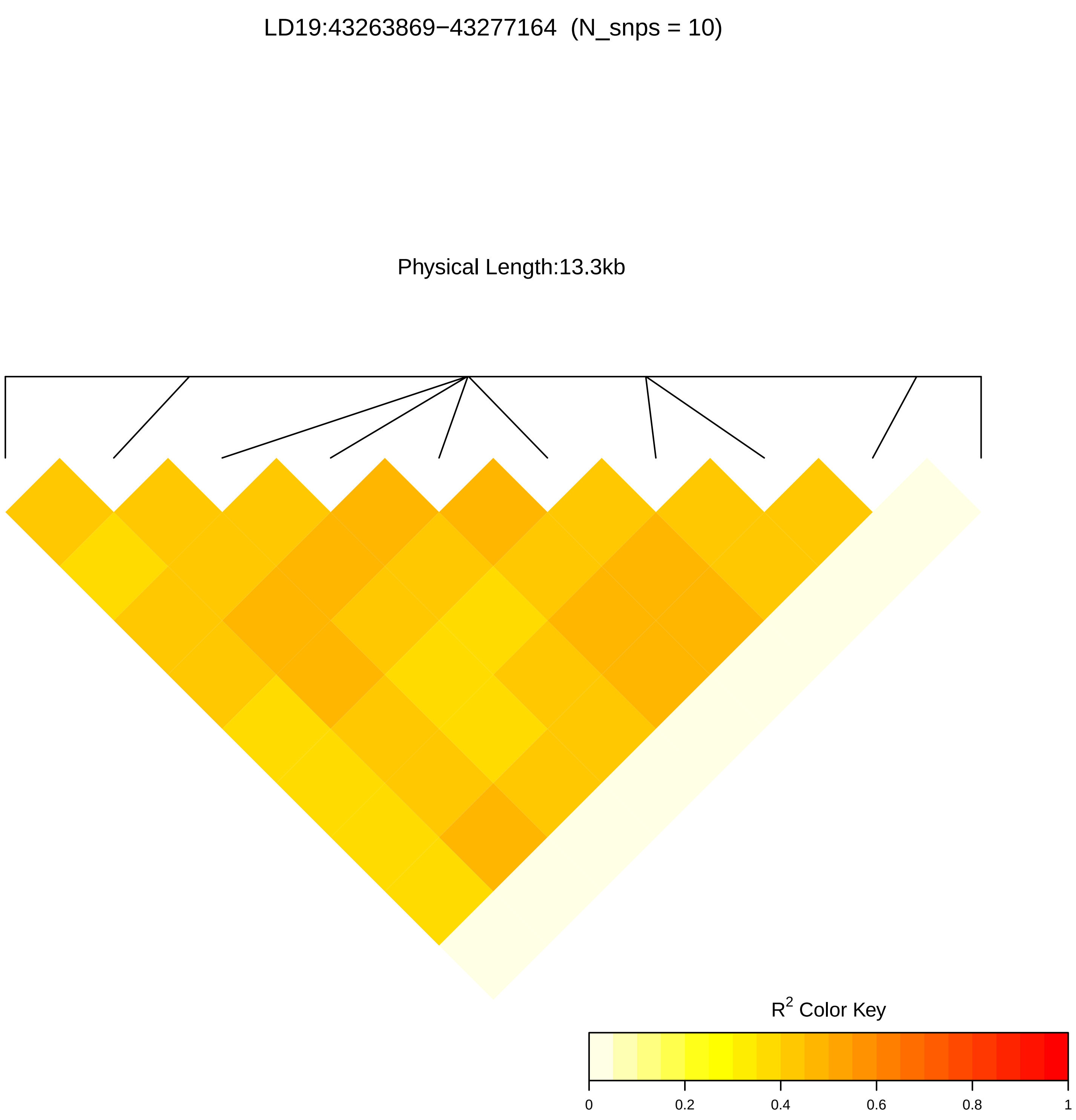
LD19:43263869−43277164 (N_snps = 10)
Physical Length:13.3kb
R2 Color Key
0
0.2
0.4
0.6
0.8
1

LD21:58941106−58962580 (N_snps = 11)

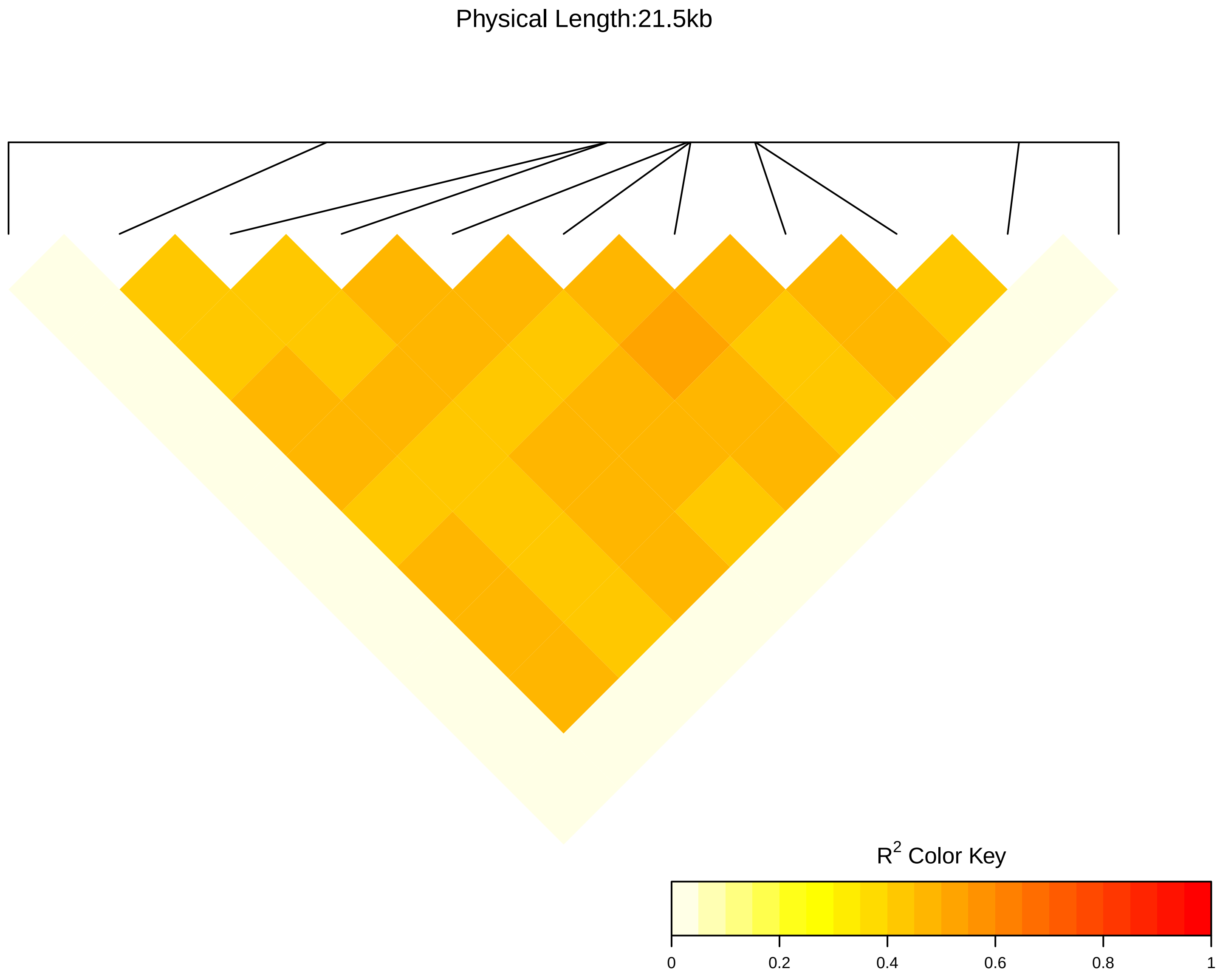

LD21:69831448−69844336 (N_snps = 18)

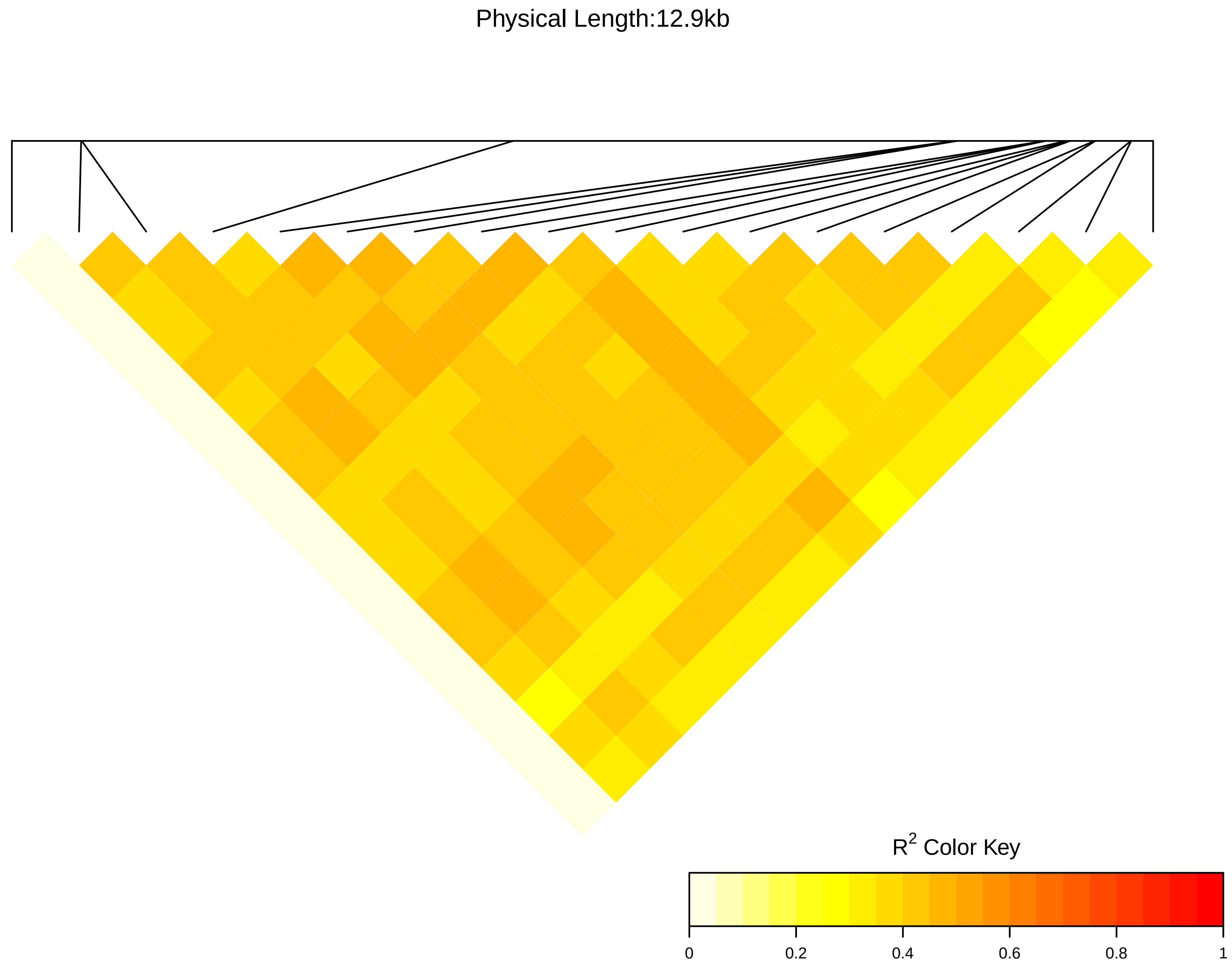

LD22:8699801−8723909 (N_snps = 18)

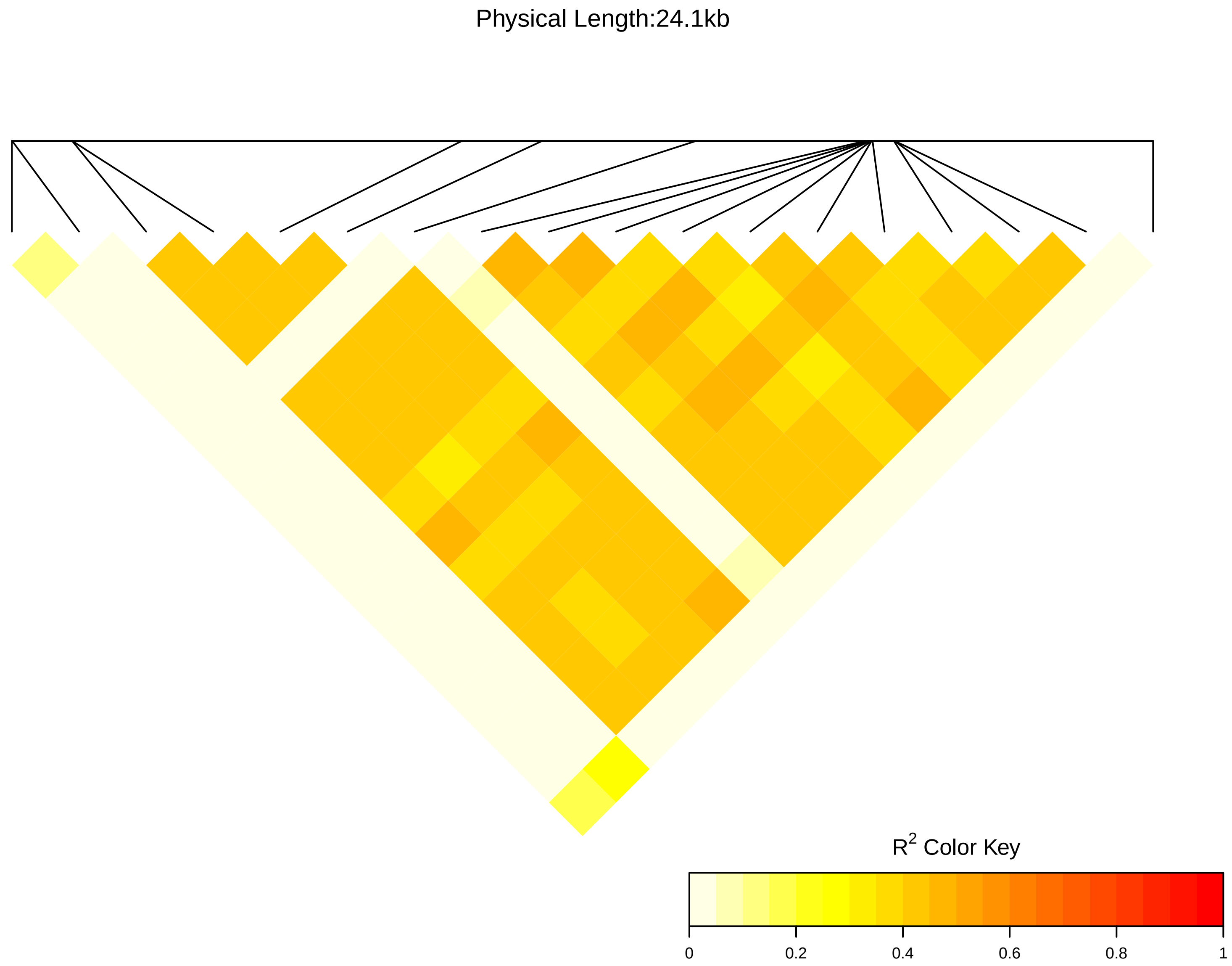

LD23:21755400−21781579 (N_snps = 23)

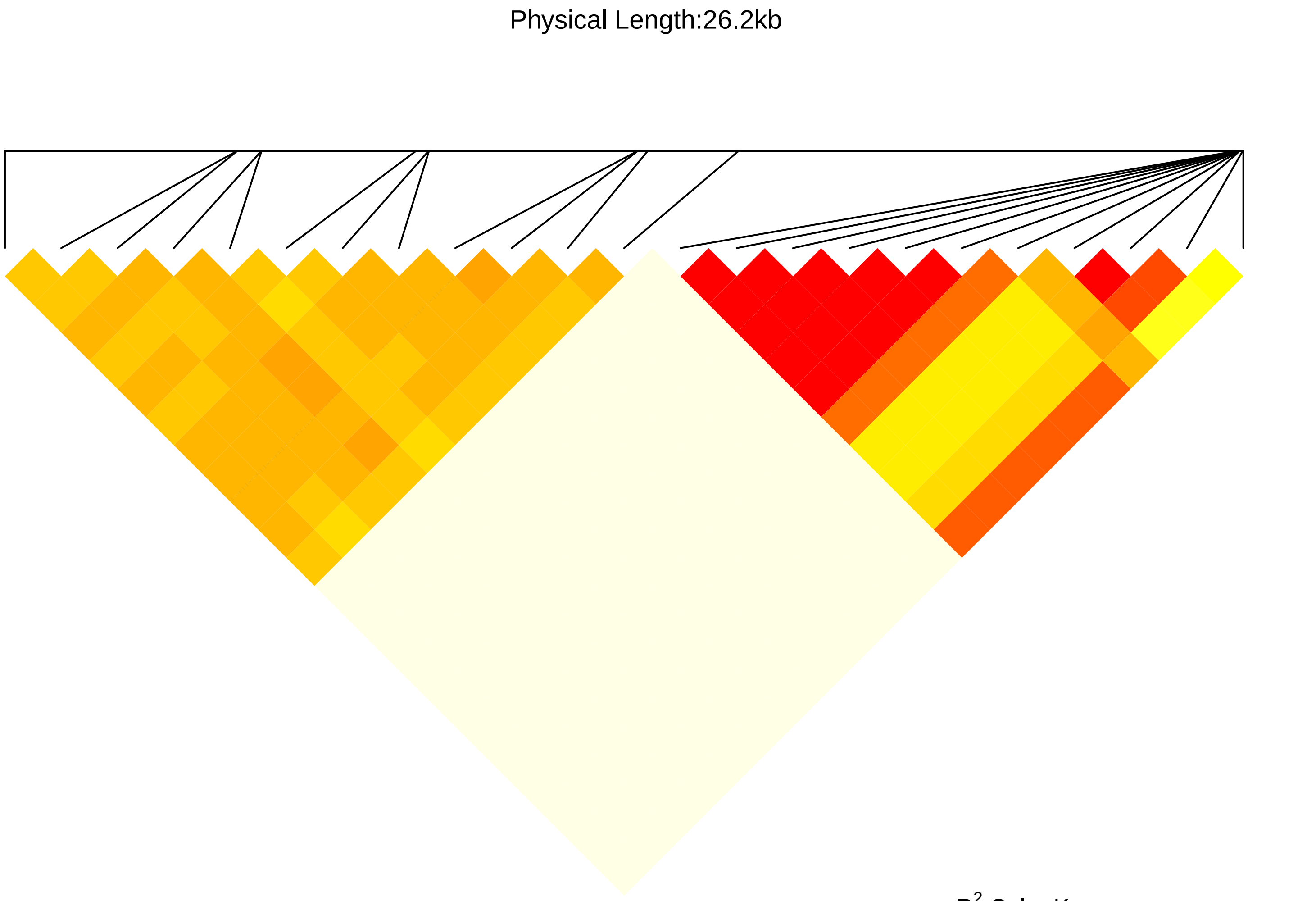

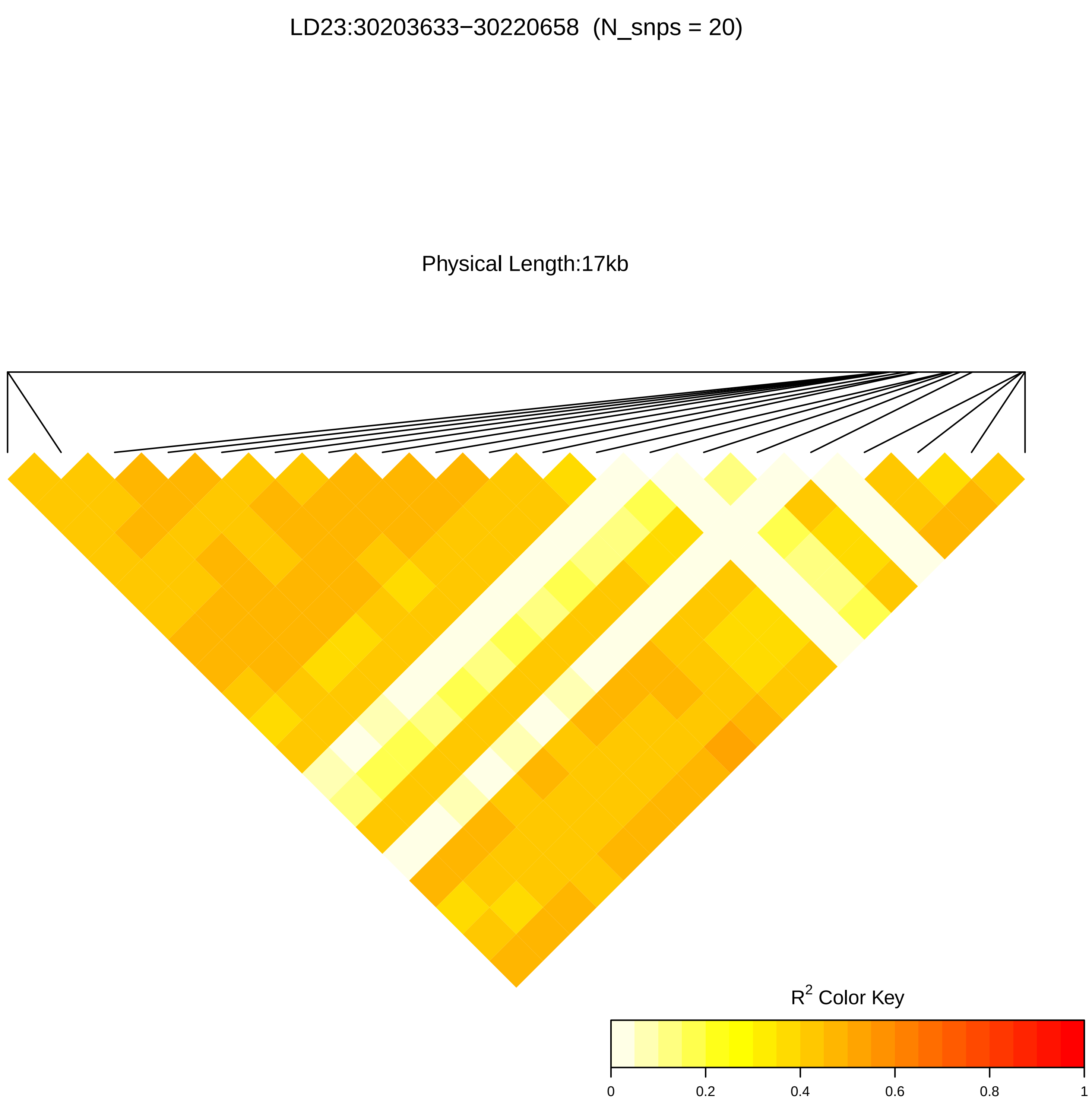
LD23:30203633−30220658 (N_snps = 20)
Physical Length:17kb
R2 Color Key
0
0.2
0.4
0.6
0.8
1

LD23:30303856−30326074 (N_snps = 25)

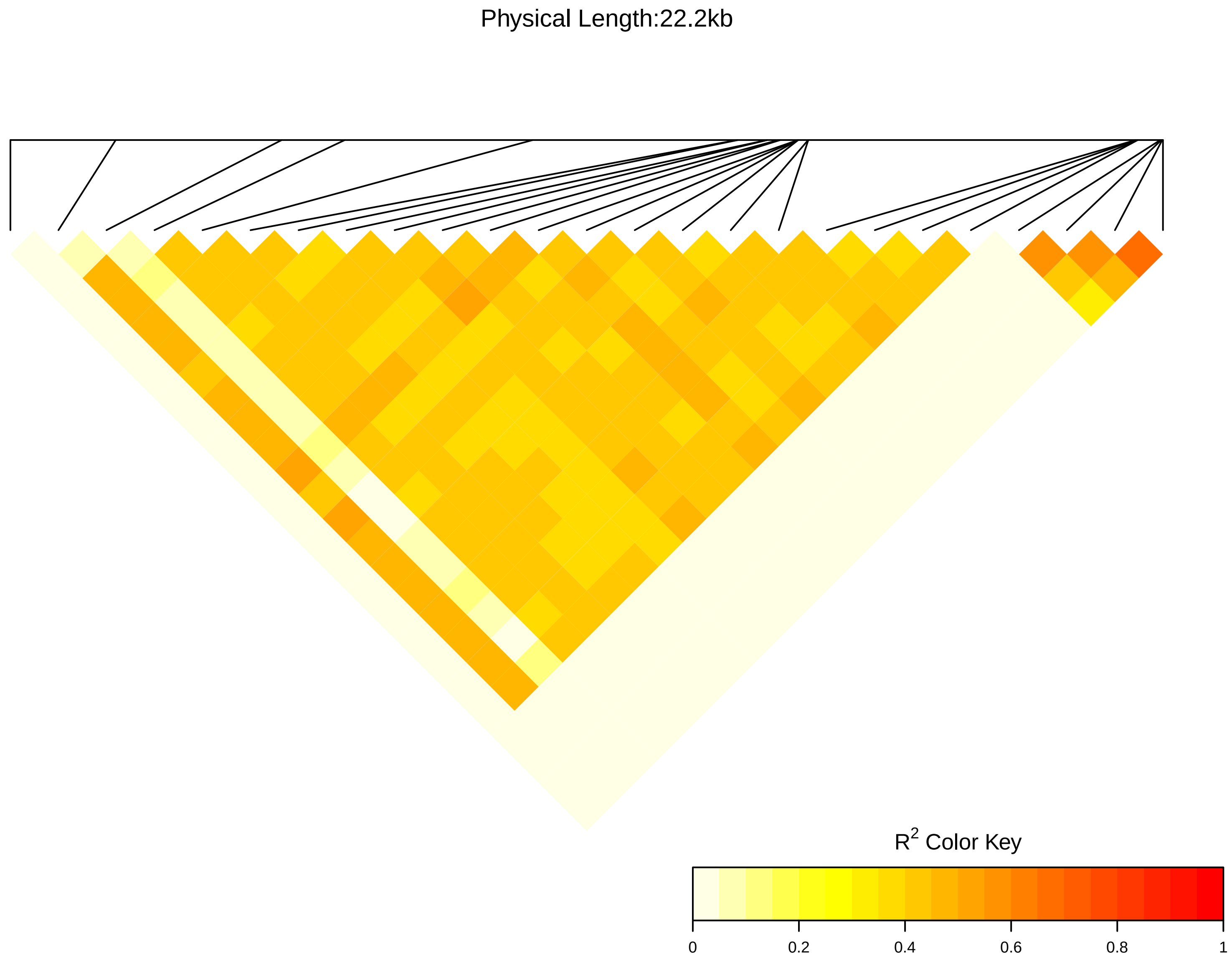

LD24:22868170−22890804 (N_snps = 36)

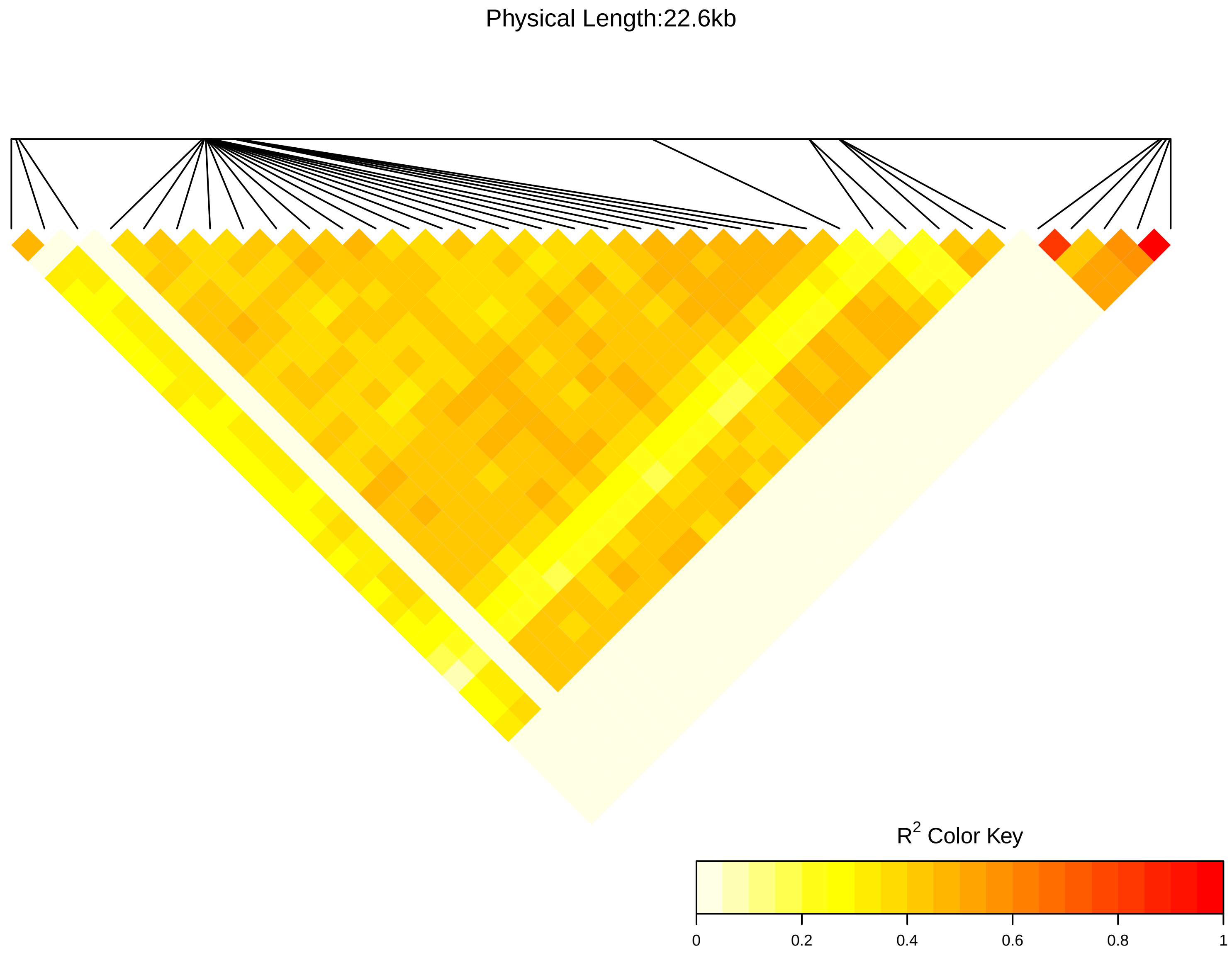

LD26:15215724−15230752 (N_snps = 13)

Physical Length:15kb

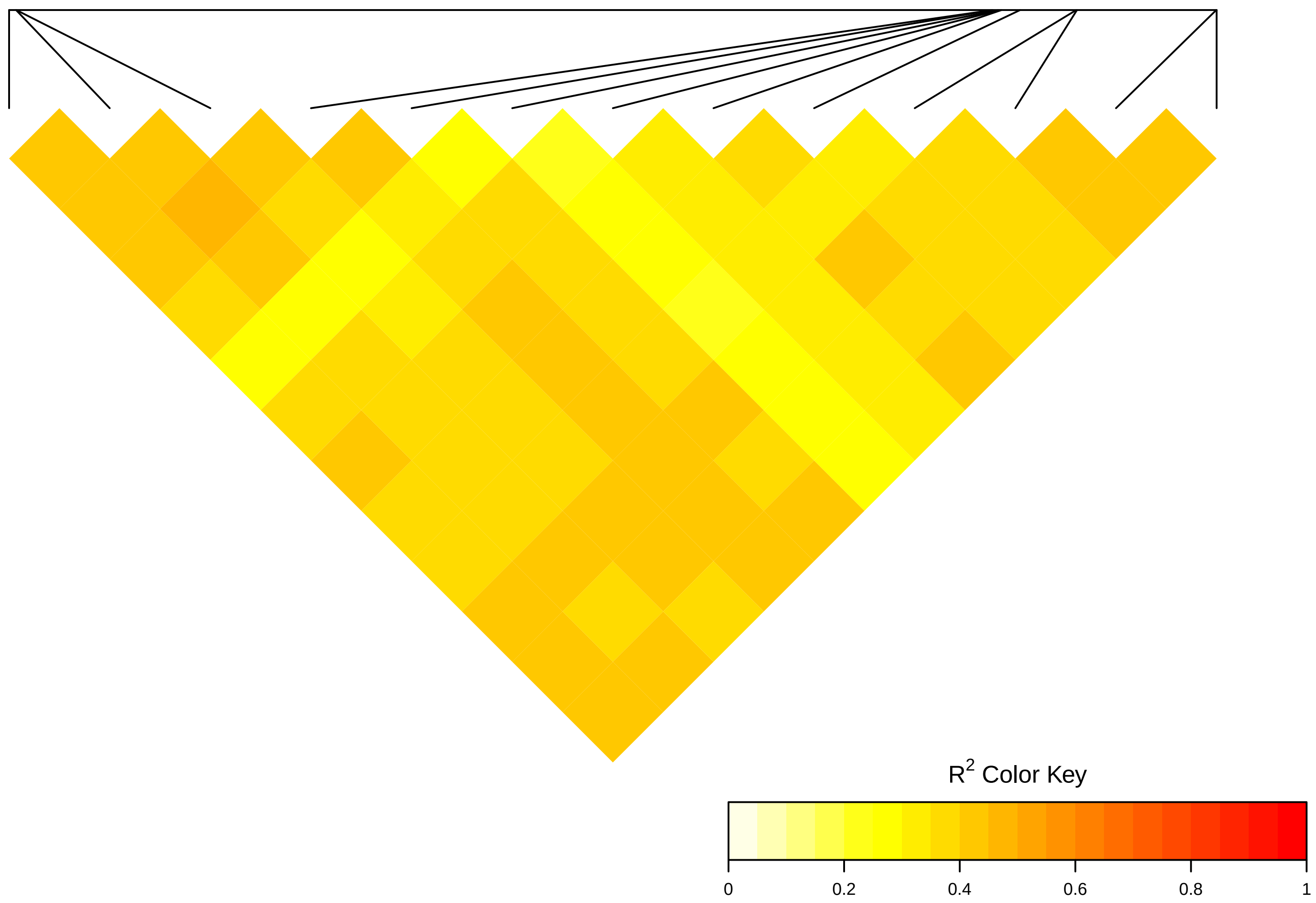

LD26:50115341−50125621 (N_snps = 11)

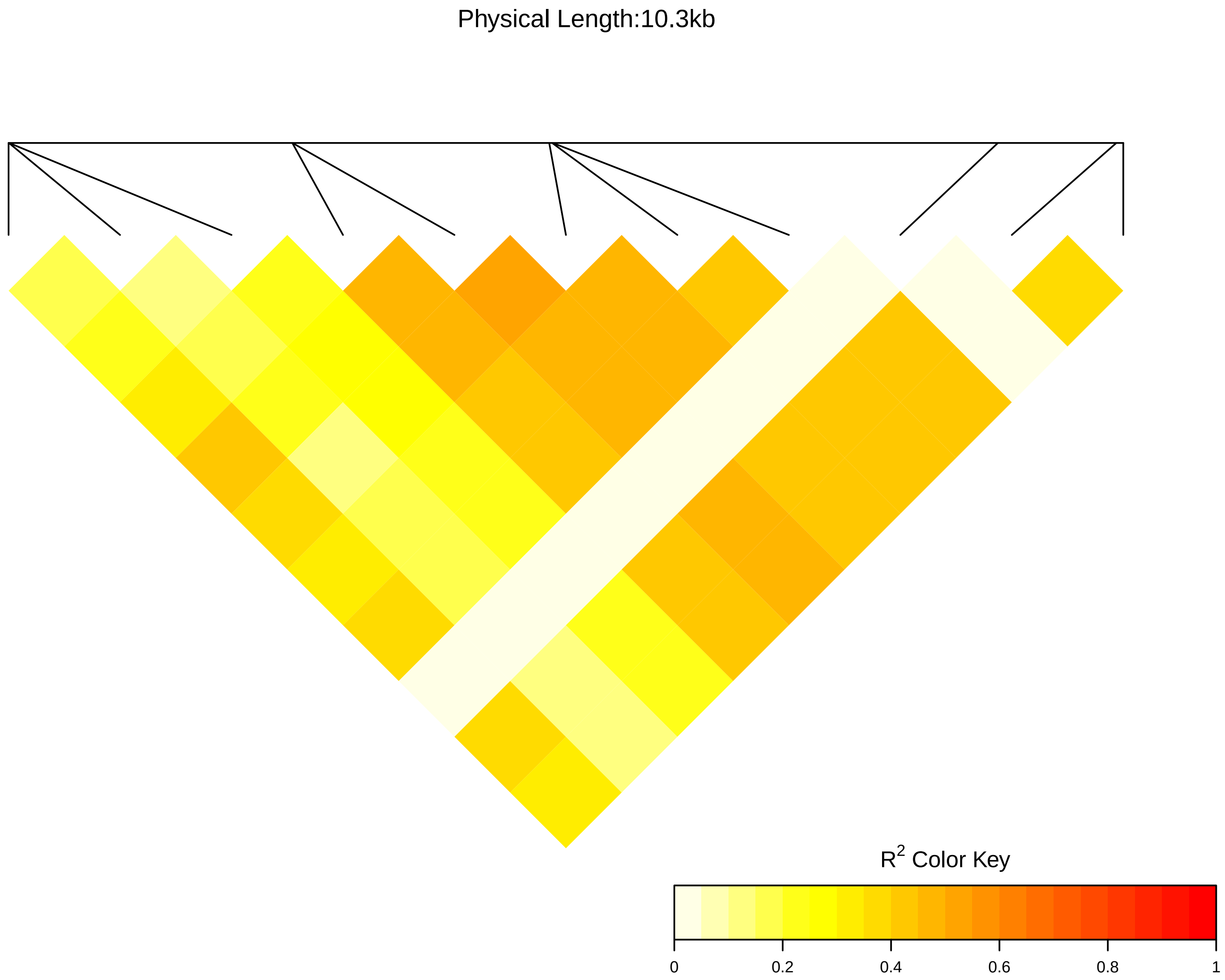

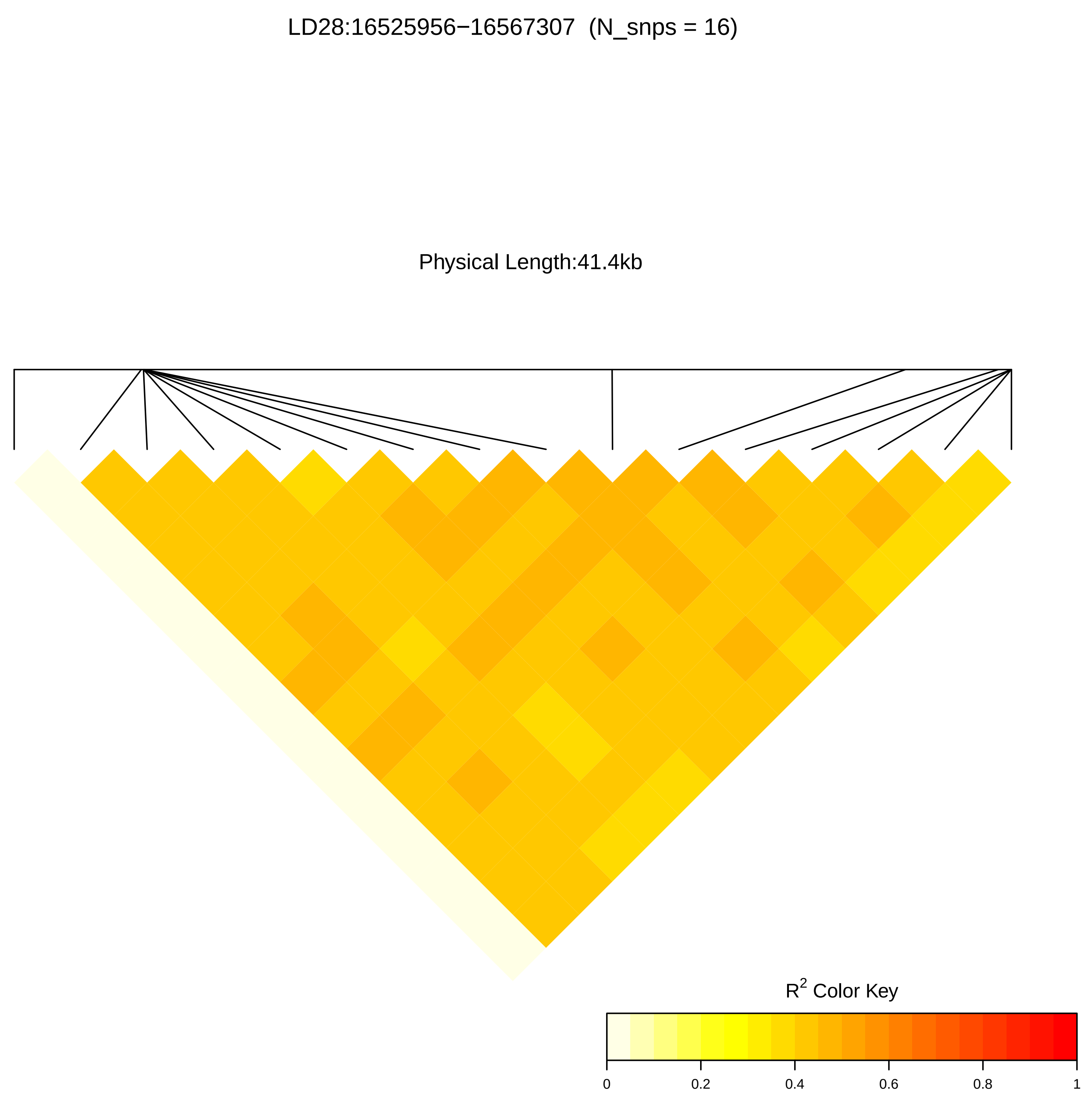
LD28:16525956−16567307 (N_snps = 16)
Physical Length:41.4kb
R^2 Color Key
0
0.2
0.4
0.6
0.8
1
